# Global, regional, and national burden of myelodysplastic syndromes and myeloproliferative neoplasms, 1990-2021: an analysis from the global burden of disease study 2021

**DOI:** 10.3389/fonc.2025.1559382

**Published:** 2025-03-18

**Authors:** Xinyue Gou, Zhuo Chen, Yudi Shangguan

**Affiliations:** ^1^ China Academy of Chinese Medical Sciences, Beijing, China; ^2^ Xiyuan Hospital, China Academy of Chinese Medical Sciences, Beijing, China; ^3^ Shanxi University of Traditional Chinese Medicine, Taiyuan, China

**Keywords:** myelodysplastic syndromes, myeloproliferative neoplasms, global burden of disease, disability-adjusted life years, incidence, deaths

## Abstract

**Objective:**

To analyze the trends and cross-country inequalities in the burden of Myelodysplastic syndromes (MDS) and myeloproliferative neoplasms (MPN) over the past 30 years and forecast potential changes through 2045.

**Methods:**

Estimates and 95% uncertainty intervals (UIs) for incidence, deaths, and disability-adjusted life-years (DALYs) associated with MDS/MPN were obtained from the Global Burden of Diseases (GBD) 2021 database. We described the epidemiology of MDS/MPN at global, regional, and national levels, analyzed trends in the burden of MDS/MPN from 1990 to 2021 through overall, local, and multidimensional perspectives, decomposed the burden based on population size, age structure, and epidemiological changes, quantified cross-country inequalities in MDS/MPN burden using standard health equity methods recommended by the WHO, and predicted changes of MDS/MPN burden to 2045.

**Results:**

The global incidence of MDS/MPN has shown a marked increase, escalating from 171,132 cases in 1990 to 341,017 cases in 2021. Additionally, the burden was found to be significantly greater in men compared to women. The overall global burden of MDS/MPN exhibited a consistent increase from 1990 to 2021, although the growth rate showed a noticeable slowdown between 2018 and 2021. Decomposition analysis identified population growth as a key factor influencing the variations in the burden of MDS/MPN. An inequality analysis across countries indicated that high Socio-demographic Index (SDI) countries bore a disproportionate share of the MDS/MPN burden, with significant SDI-related disparities remaining evident. Interestingly, while the incidence and deaths of MDS/MPN, along with the age-standardized rate (ASR) for DALYs, are projected to decline annually from 2020 to 2045, the absolute number of cases for these indicators is expected to continue rising. By 2045, the projected numbers are estimated to reach 457,320 cases for incidence, 82,047 cases for deaths, and 1,689,518 cases for DALYs.

**Conclusions:**

As a major public health issue, the global burden of MDS/MPN showed an overall increasing trend from 1990 to 2021, which was primarily driven by population growth and aging. The largest share of the MDS/MPN burden was seen primarily in men, with older demographics. Countries with elevated SDI experienced a significantly higher burden of MDS/MPN. While the burden of MDS/MPN was most pronounced in high SDI quintile, the fastest growth was observed in the low-middle SDI quintile, especially in tropical Latin America. This study highlighted great challenges in the control and management of MDS/MPN, including both growing case number and distributive inequalities worldwide. These findings provide valuable insights for developing more effective public health policies and optimizing the allocation of medical resources.

## 1 Introduction

Myelodysplastic syndromes (MDS) and myeloproliferative neoplasms (MPN) are clonal hematopoietic disorders of the bone marrow characterized by hematopoietic defects that lead to impaired hematopoiesis and/or abnormal cellular proliferation, with a risk of transformation into acute myeloid leukemia (AML) (1, 2). Based on the 2016 WHO classification, the category of MPN comprises several well-defined types, including chronic myeloid leukemia (CML), MDS/MPN, chronic neutrophilic leukemia (CNL), polycythemia vera (PV), essential thrombocythemia (ET), primary myelofibrosis (PMF), and chronic eosinophilic leukemia not otherwise specified (CEL-NOS), as well as unclassifiable MPN (MPN-U) (3, 4). MDS is more commonly observed in older men, with a median age of diagnosis of 76 years in the United States (5). The age-adjusted incidence rate in the United States of America for 2015 was reported at 4.0 cases per 100,000 persons (6). The survival rate for MDS patients remains poor, with a reported 3-year survival rate of only 35%, which is significantly lower among male patients and those of older age (6, 7). Hypomethylating agents (HMA), such as azacitidine and decitabine, are regarded as the standard of care for patients with MDS (2, 8, 9). Although the use of these agents may improve survival rates, they are only life-prolonging and not curative (9). Consequently, allogeneic hematopoietic stem cell transplantation (HSCT) is increasingly utilized as a potential curative treatment option (10); however, it is associated with severe complications and requires substantial financial resources, imposing significant health and economic burdens globally.

Similarly, MPN primarily affected middle-aged and older adults between the ages of 50 and 70 (11, 12). In the United States, the incidence rate for CML, PV, and ET was relatively similar, ranging from 1.0 to 2.0 cases per 100,000 persons, whereas PMF is much rarer, with an incidence of 0.3 cases per 100,000 persons (13). Patients with PMF exhibited a lower survival rate compared to those with ET or PV (14), with a five-year mortality rate of 51.0% (13). Furthermore, MPN is associated with an increased risk of thrombotic events and leukemic transformation (15, 16).

Given the global trend of an aging population (17, 18), the burden of disease caused by MDS/MPN has become an urgent concern. Most previous studies have investigated the incidence of MDS/MPN in high-income, developed countries with good health care infrastructures, including Italy (19), Finland (20), Spain (21), the United States (22, 23), Japan (24) and the United Kingdom (25). However, to date, there has been no comprehensive analysis of the global burden of MDS/MPN utilizing the latest Global Burden of Disease (GBD) 2021 data. In this study, we analyzed data from the GBD study spanning from 1990 to 2021 to identify changes in MDS/MPN incidence, deaths, and disability-adjusted life years (DALYs) at global, regional, and national levels, aiming to inform accurate screening and diagnosis efforts. Our analysis was stratified data by sex, age, and Sociodemographic Index (SDI) to identify populations most affected by MDS/MPN and to guide targeted management measures and treatment strategies. Additionally, we projected the global burden of MDS/MPN through to the year 2045.

## 2 Methods

### 2.1 Data source

The GBD 2021 (26) provided a thorough evaluation of health loss attributable 371 diseases, injuries, and impairments, as well as 88 risk factors, taking into account age and sex across 204 countries and territories (27, 28), (**Supplementary Table S1**). For a majority of the diseases and injuries examined, the data was analyzed through spatiotemporal Gaussian process regression to project study populations based on factors such as age, sex, geographical location, and year. The research employed DisMod-MR in conjunction with the Cause of Death Ensemble Model (CODEm) as the main tools for standardization. DisMod-MR functioned as a Bayesian meta-regression method that evaluated incidence, remission, and disease-associated deaths while simultaneously improving consistency in epidemiological metrics. CODEm served as a systematic approach for examining cause-of-death information, utilizing various modeling techniques to assess ratios or cause scores (29).

The information pertinent to this research was gathered from the Global Health Data Exchange (GHDx) and its associated tool (http://ghdx.healthdata.org/gbdresults-tool). The estimates, along with their 95% uncertainty intervals (UIs), regarding incidence, deaths and DALYs, and relevant age-standardized rate (ASR) for MDS/MPN were derived from GBD 2021. All rates were expressed per 100,000 persons. The incidence and deaths related to MDS/MPN were identified according to the International Classification of Diseases, Tenth Revision (ICD-10). Specifically, the MDS/MPN was determined through ICD-10 codes D45, D46, and D47, while the corresponding deaths was established using the same ICD-10 codes. Additionally, the SDI, which served as a composite measure reflecting income, education, and fertility indicators to assess the level of sociodemographic development in a country or region, encompassing five classifications based on SDI quintiles (namely, high, high-middle, middle, low-middle, low), was also utilized (30, 31).

### 2.2 Trend analysis

Initially, we employed the estimated annual percentage change (EAPC) to assess the general trend of the MDS/MPN burden. Standardization was essential when analyzing multiple groups with varying age structures or a specific group whose age profile changes over time. Consequently, the trend of ASR, gauged by EAPC, served as a more reliable metric for tracking shifts in disease patterns. We developed a linear regression model represented by the equation y = α + βx, where y denotes ln (ASR) and x corresponds to the calendar year. The EAPC was subsequently computed using the formula (exp(β)-1) *100%, and the 95% confidence interval (CI) was also derived from this model. If both the EAPC estimate and its lower boundary of the 95% CI were greater than 0, it indicated that the ASR was on an upward trend. Conversely, if both the EAPC estimate and its upper boundary of the 95% CI were less than 0, it suggested that the ASR was on a downward trend. In all other cases, the ASR was considered to be stable.

Subsequently, joinpoint regression analysis was employed to identify the local trend of the MDS/MPN burden, specifically utilizing a log-linear model (ln y = xb). This method calculated the annual percentage change along with its 95% CI to characterize the trend over a defined time frame. An upward trend was considered to be present during a specific period if both the average annual percentage change (AAPC) estimate and its lower boundary of the 95% CI were greater than zero. Conversely, if both the AAPC estimate and its upper boundary of the 95% CI were less than zero, a downward trend was identified for that period. If neither condition was met, the trend was classified as stable (32).

Additionally, we investigated the patterns of MDS/MPN burden linked to age, period, and birth cohort influences (33). In our model, the effects of age signified the alterations in the variable throughout an individual’s life, while period effects illustrated how environmental elements impacted the whole population. Additionally, birth cohort effects referred to the variations in the variable among children who encountered comparable life events because of their shared birth year (26, 34). We utilized an age-period-cohort (APC) model employing the intrinsic estimator (IE) technique, incorporating principal component regression analysis to capture the varying influences across three temporal dimensions and to yield more efficient estimates. The APC model was articulated as follows: In (Refg) = α + Ae + Pf + Cg, where Refg represented the incidence or deaths rate in the cohort g, e denoted the age category, and f referred to the time period, with Ae, Pf, and Cg symbolizing the impacts of age, period, and cohort, respectively. This model was developed to ascertain the relative risk (RR) for incidence and deaths associated with a specific age, time period, or birth cohort when compared to the mean combined level across all ages, periods, or birth cohorts.

### 2.3 Cross-country inequality analysis

In the current research, we employed two established metrics to assess the levels of distributive inequality in the burden of MDS/MPN across different countries. These metrics include the slope index of inequality as well as the concentration index. By utilizing these standard measures of both absolute and relative gradient inequality, we aimed to generate a clearer understanding of how the burden of MDS/MPN is distributed across various nations. The slope index of inequality was derived by regressing national incidence, deaths, and DALYs across the entire population on a scale reflecting sociodemographic development-related relative positions. Meanwhile, the concentration index was determined through the numerical integration of the area under the Lorenz concentration curve, which was constructed based on the cumulative fractions of incidence, deaths, and DALYs along with the cumulative relative distribution of the population ranked according to SDI.

### 2.4 Decomposition analysis

To explore the extent to which the influences of population growth, aging, and epidemiological changes have affected MDS/MPN epidemiology over the past three decades, an analysis of DALYs was conducted considering demographics, age distribution, and epidemiological changes. Epidemiological changes refer to changes in age-and population-adjusted incidence and deaths (35). The impact of each element on the variations in DALYs from 1990 to 2021 was characterized by assessing the effect of modifying a specific factor, while maintaining the other factors unchanged.

### 2.5 Predictive analysis

In order to enhance the formulation of public health policies and the distribution of healthcare resources, an additional forecast regarding the MDS/MPN burden over the upcoming decades was conducted. The Bayesian age-period-cohort (BAPC) analysis model, which showed superior coverage and precision compared to the traditional APC model, was employed to project the global MDS/MPN burden up to 2045. Under the assumption that the effects of age, period, and adjacent cohorts exhibit similarity, the Bayesian inference in age-period-cohort model applies second-order stochastic smoothing as a prior for age, period, and cohort effects and to project posterior mortality rates. This method utilizes integrated nested Laplace approximations to estimate the marginal posterior distributions, effectively addressing the mixing and convergence challenges that are typically encountered with conventional Markov chain Monte Carlo sampling techniques in Bayesian analysis (36).

All data analyses were conducted using R software package (version 4.4.1) and Joinpoint software (version 5.2.0.0), with a significance level set at P < 0.05.

## 3 Results

### 3.1 Descriptive analysis of MDS/MPN burden at global, regional, and national levels

**Figure 1:**
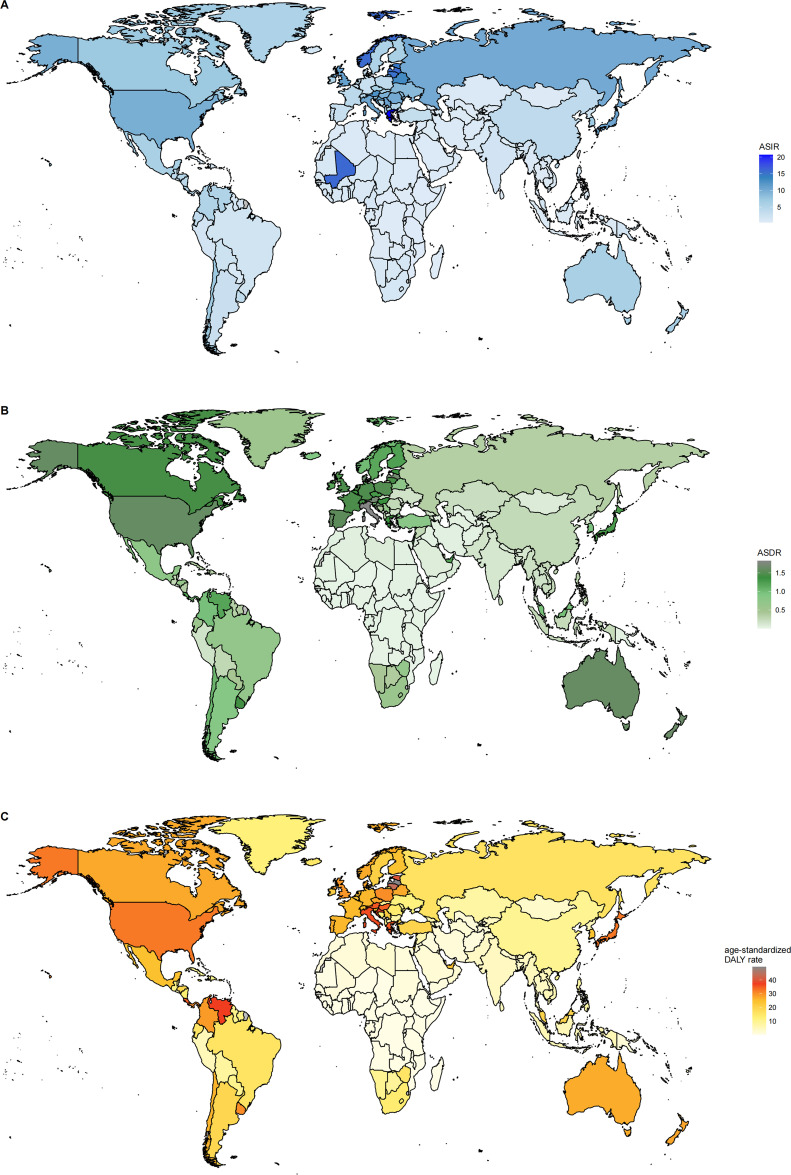
**(A)** The ASR of incidence in 2021; **(B)** The ASR of deaths in 2021; **(C)** The ASR of DALYs in 2021. ASR, age-standardized rate per 100,000 persons; DALYs, disability-adjusted life-years.

**Figure 2:**
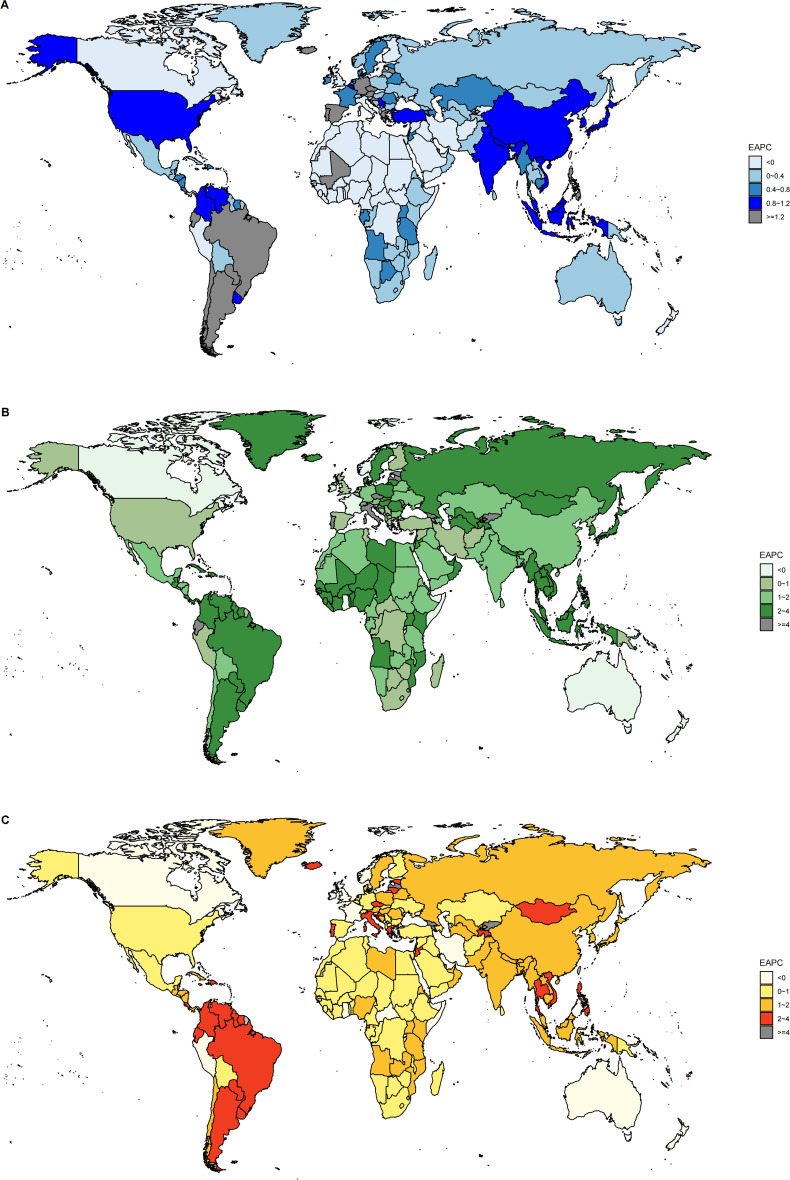
**(A)** The trend in ASR of incidence (EAPC) from 1990 to 2021; **(B)** The trend in ASR of deaths (EAPC) from 1990 to 2021; **(C)** The trend in ASR of DALYs (EAPC) from 1990 to 2021. ASR, age-standardized rate per 100,000 persons; EAPC, estimated annual percentage change; DALYs, disability-adjusted life-years.

**Figure 3:**
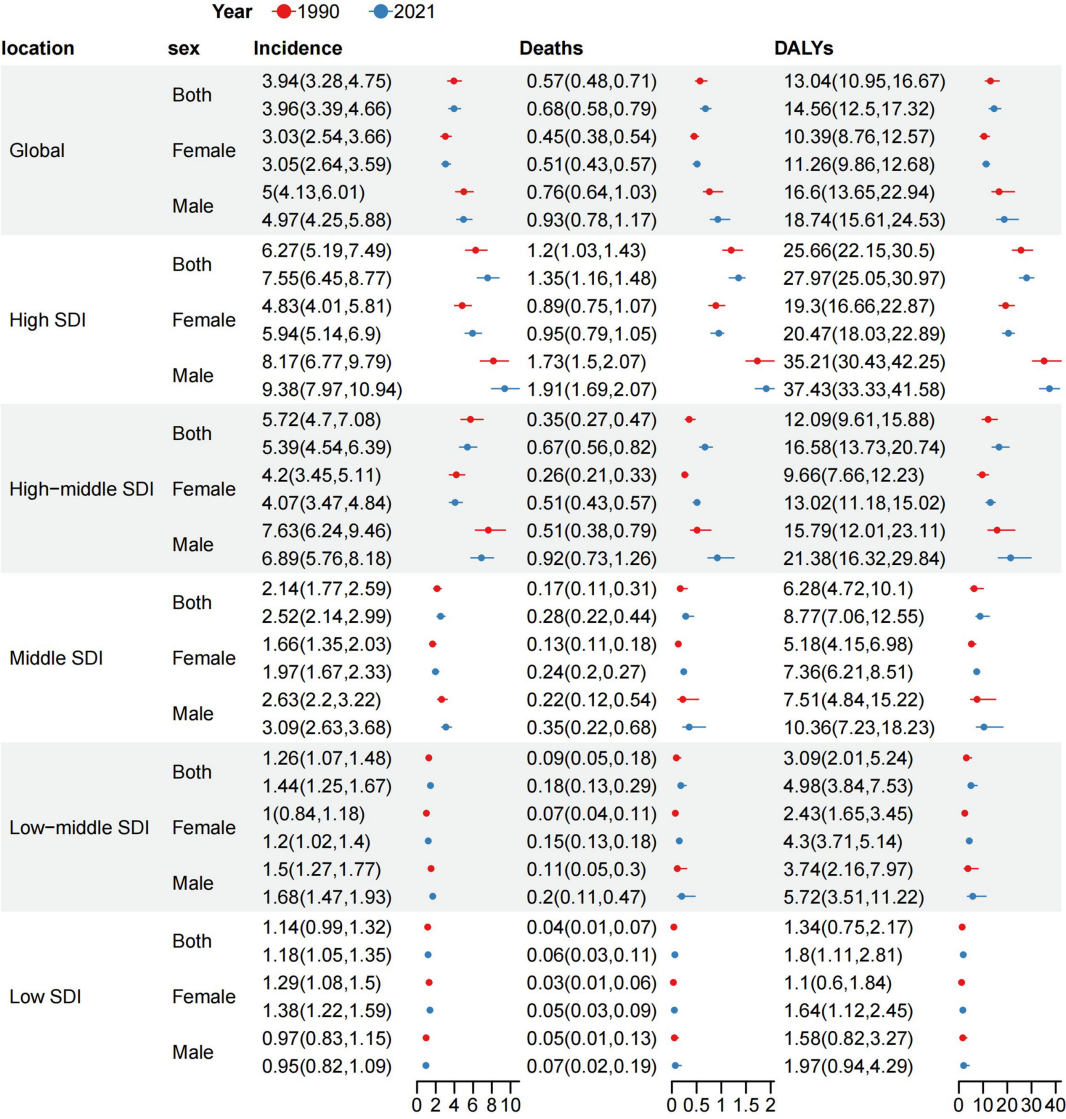
ASR of incidence, deaths and DALYs by SDI quintiles and sex subgroups at the global level in 1990 and 2021. ASR, age-standardized rate per 100,000 persons; DALYs, disability-adjusted life-years; SDI, socio-demographic index.

**Figure 4:**
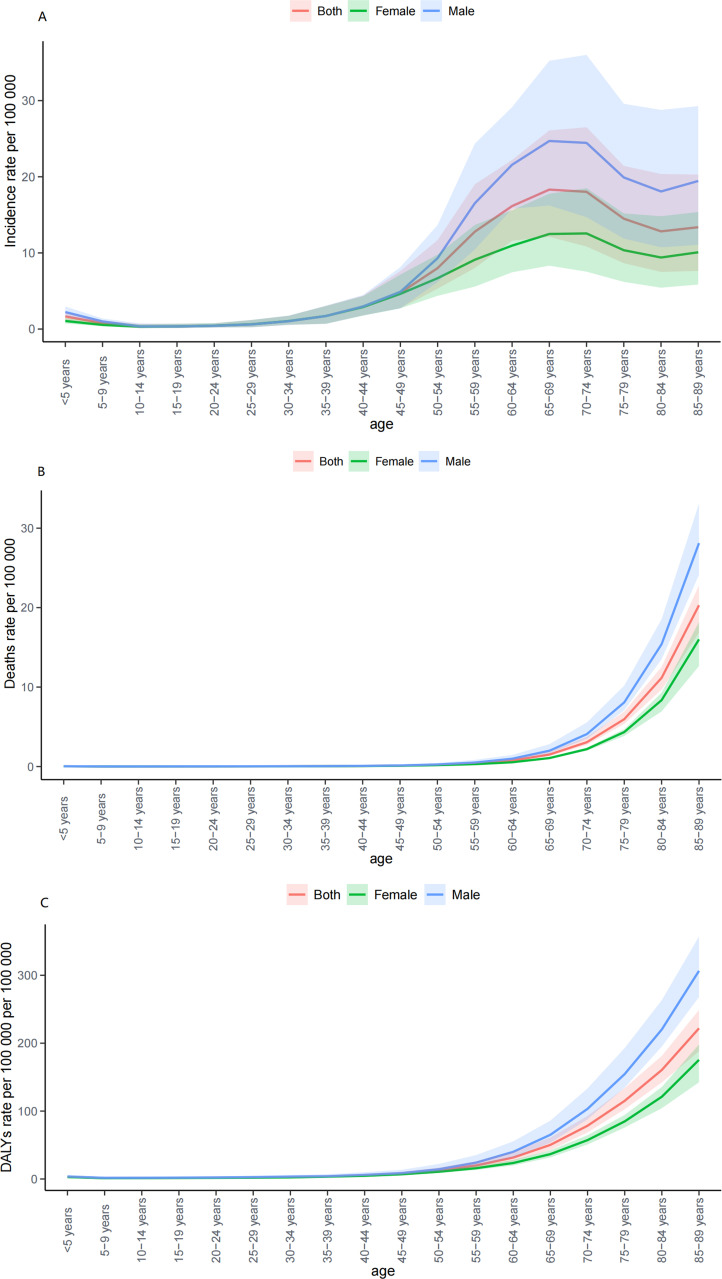
The trend in ASR of incidence **(A)**, deaths **(B)** and DALYs **(C)** by sex and age, 1990-2021. ASR, age-standardized rate per 100,000 persons; DALYs, disability-adjusted life-years; SDI, socio-demographic index.

Globally, the number of cases and the crude rates for incidence, deaths, and DALYs exhibited significantly upward trends whereas the ASR for these three measures displayed modest increases from 1990 to 2021, higher in men than in women (**Tables 1**–**3**, **Figures 1**–**4**). In 2021, the case number of incidence, deaths, and DALYs were 341,017, 55,135, and 1,214,107 per 1000, respectively. According to the SDI quintiles, the high SDI quintile in 2021 reported the highest numbers of cases and ASR for MDS/MPN incidence, deaths, and DALYs. As for ASR, the higher the SDI quintiles, the higher the number of cases and ASR. The highest ASR of incidence, deaths, and DALYs were observed in the high SDI quintile (**Figures 1**, **3**). Regionally, East Asia reported the highest number of cases, high-income North America had the highest number of deaths, and Western Europe experienced the greatest burden cases. Eastern Europe exhibited the highest ASR of incidence, while high-income North America recorded the highest ASR of deaths and DALYs. On a national scale, the incidence, deaths, and DALYs of MDS/MPN showed significant variation globally, with the highest number of incidences cases found in China, while the United States had the highest number of deaths and cases of DALYs. Furthermore, Greece showcased the highest ASR of incidence, Italy reported the highest ASR of mortality, and Latvia documented the highest ASR of DALYs (**Figure 1**; **Supplementary Tables S2–S5**).

**Table 1:** The case number and ASR of incidence of MDS/MPN in 1990 and 2021 by SDI quintiles and by GBD regions, with EAPC from 1990 to 2021.

Location	1990		2021		1990-2021 EAPCs (95% CI)
	Incidence cases (95% CI)	ASIR (95% CI)	Incidence cases (95% CI)	ASIR (95% CI)	
Global	171.13 (143.36,206.9)	3.94 (3.28,4.75)	341.02 (290.88,401.9)	3.96 (3.39,4.66)	0.14 (0.07,0.21)
SDI quintiles					
High SDI	65.47 (53.56,78.93)	6.27 (5.19,7.49)	136.58 (115.1,158.94)	7.55 (6.45,8.77)	0.56 (0.49,0.62)
High-middle SDI	60.13 (49.37,74.89)	5.72 (4.7,7.08)	101.62 (84.43,121.55)	5.39 (4.54,6.39)	-0.04 (-0.14,0.06)
Middle SDI	29.2 (24.46,35.02)	2.14 (1.77,2.59)	69.51 (58.28,83.64)	2.52 (2.14,2.99)	0.85 (0.69,1.01)
Low-middle SDI	12.19 (10.32,14.33)	1.26 (1.07,1.48)	24.45 (21.07,28.6)	1.44 (1.25,1.67)	0.67 (0.58,0.77)
Low SDI	3.94 (3.32,4.61)	1.14 (0.99,1.32)	8.57 (7.4,9.95)	1.18 (1.05,1.35)	0.3 (0.21,0.39)
GBD regions					
Andean Latin America	0.57 (0.48,0.69)	2.1 (1.73,2.56)	1.51 (1.27,1.76)	2.46 (2.07,2.88)	0.57 (0.41,0.74)
Australasia	1.45 (1.16,1.81)	6.03 (4.87,7.43)	3.26 (2.64,3.97)	6.02 (4.96,7.3)	-0.31 (-0.45,-0.17)
Caribbean	0.46 (0.39,0.54)	1.51 (1.29,1.79)	0.85 (0.73,0.99)	1.73 (1.49,2.02)	0.5 (0.46,0.53)
Central Asia	0.37 (0.31,0.45)	0.67 (0.56,0.82)	0.65 (0.54,0.78)	0.7 (0.59,0.84)	0.21 (0.17,0.24)
Central Europe	9.21 (7.56,11.26)	6.27 (5.19,7.6)	12.9 (10.79,15.22)	6.86 (5.84,7.99)	0.42 (0.05,0.79)
Central Latin America	5.47 (4.63,6.54)	4.8 (3.98,5.83)	14.12 (11.86,17.01)	5.56 (4.69,6.66)	0.48 (0.38,0.59)
Central Sub-Saharan Africa	0.2 (0.15,0.25)	0.52 (0.44,0.62)	0.45 (0.36,0.56)	0.53 (0.46,0.61)	0.08 (0.03,0.13)
East Asia	33.37 (27.01,41.33)	3.16 (2.56,3.92)	82.52 (67.4,101.11)	3.82 (3.17,4.63)	0.98 (0.79,1.17)
Eastern Europe	25.53 (20.3,32.53)	9.17 (7.36,11.45)	31.88 (25.29,39.24)	10.14 (8.17,12.48)	0.33 (0.31,0.34)
Eastern Sub-Saharan Africa	0.6 (0.47,0.77)	0.49 (0.41,0.58)	1.27 (1.03,1.58)	0.52 (0.46,0.6)	0.24 (0.23,0.25)
High-income Asia Pacific	14.57 (11.68,18.16)	7.73 (6.31,9.38)	29.08 (23.7,34.73)	9.59 (8.07,11.39)	0.87 (0.78,0.95)
High-income North America	26.05 (20.66,32.4)	7.6 (6.11,9.3)	56.61 (47.1,67.56)	9.46 (8.03,11.08)	0.75 (0.47,1.03)
North Africa and Middle East	3.23 (2.75,3.81)	1.23 (1.03,1.44)	6.81 (5.85,7.9)	1.19 (1.04,1.35)	0.16 (-0.13,0.44)
Oceania	0.08 (0.06,0.09)	1.52 (1.28,1.79)	0.19 (0.17,0.23)	1.69 (1.48,1.95)	0.38 (0.34,0.43)
South Asia	13.88 (11.59,16.57)	1.38 (1.16,1.62)	27.97 (23.55,33.39)	1.63 (1.39,1.92)	0.88 (0.73,1.04)
Southeast Asia	2.43 (1.99,2.91)	0.73 (0.6,0.87)	6.4 (5.36,7.64)	0.92 (0.78,1.07)	0.93 (0.85,1)
Southern Latin America	1.28 (1.05,1.55)	2.69 (2.22,3.28)	3.48 (2.92,3.99)	4.23 (3.56,4.84)	1.71 (1.55,1.87)
Southern Sub-Saharan Africa	0.59 (0.49,0.7)	1.39 (1.17,1.62)	0.98 (0.84,1.14)	1.46 (1.26,1.69)	0.12 (0.08,0.17)
Tropical Latin America	0.64 (0.54,0.77)	0.62 (0.51,0.75)	4.79 (3.9,5.77)	1.89 (1.55,2.26)	4.51 (4.19,4.83)
Western Europe	29.46 (23.71,36.06)	5.46 (4.44,6.68)	51.36 (43.22,60.02)	6.37 (5.42,7.45)	0.33 (0.16,0.5)
Western Sub-Saharan Africa	1.69 (1.39,2.01)	1.63 (1.3,1.96)	3.93 (3.38,4.57)	1.74 (1.48,2.01)	0.27 (0.24,0.31)

Incidence cases: number of cases/thousands; ASIR: age-standardized incidence rate per 100,000 persons;

ASR, age-standardized rate; SDI, socio-demographic index; GBD, global burden of diseases, injuries, and risk factors study; EAPC, estimated annual percentage change; CI, confidence interval; MDS/MPN, myelodysplastic syndromes/myeloproliferative neoplasms.

**Table 2:** The case number and ASR of deaths of MDS/MPN in 1990 and 2021 by SDI quintiles and by GBD regions, with EAPC from 1990 to 2021.

Location	1990		2021		1990-2021
	Deaths cases (95% CI)	ASDR (95% CI)	Deaths cases (95% CI)	ASDR (95% CI)	EAPCs (95% CI)
Global	18.65 (15.97,23.95)	0.57 (0.48,0.71)	55.13 (46.73,64.25)	0.68 (0.58,0.79)	0.7 (0.64,0.76)
SDI quintiles					
High SDI	13.39 (11.56,16.02)	1.2 (1.03,1.43)	32.97 (27.99,36.15)	1.35 (1.16,1.48)	0.45 (0.39,0.5)
High-middle SDI	3.02 (2.36,4.13)	0.35 (0.27,0.47)	12.64 (10.58,15.4)	0.67 (0.56,0.82)	2.27 (1.99,2.55)
Middle SDI	1.66 (1.12,3.03)	0.17 (0.11,0.31)	6.96 (5.46,10.86)	0.28 (0.22,0.44)	1.9 (1.83,1.97)
Low-middle SDI	0.5 (0.26,0.96)	0.09 (0.05,0.18)	2.25 (1.62,3.77)	0.18 (0.13,0.29)	2.48 (2.39,2.58)
Low SDI	0.07 (0.02,0.13)	0.04 (0.01,0.07)	0.25 (0.12,0.45)	0.06 (0.03,0.11)	2 (1.73,2.27)
GBD regions					
Andean Latin America	0.03 (0.02,0.05)	0.15 (0.1,0.25)	0.17 (0.13,0.24)	0.29 (0.23,0.41)	1.94 (1.3,2.59)
Australasia	0.41 (0.33,0.53)	1.8 (1.46,2.33)	1 (0.84,1.12)	1.61 (1.37,1.8)	-0.41 (-0.51,-0.31)
Caribbean	0.08 (0.07,0.11)	0.32 (0.27,0.43)	0.27 (0.22,0.33)	0.5 (0.42,0.62)	1.86 (1.68,2.05)
Central Asia	0.02 (0.02,0.03)	0.04 (0.03,0.06)	0.08 (0.07,0.1)	0.1 (0.08,0.12)	2.72 (2.42,3.01)
Central Europe	0.63 (0.52,0.79)	0.45 (0.36,0.56)	2.29 (2.04,2.53)	0.96 (0.86,1.06)	2.86 (2.65,3.07)
Central Latin America	0.43 (0.37,0.53)	0.46 (0.39,0.56)	1.99 (1.75,2.24)	0.82 (0.72,0.92)	2.26 (2.09,2.43)
Central Sub-Saharan Africa	0 (0,0)	0.01 (0.01,0.02)	0.01 (0,0.01)	0.02 (0.01,0.03)	0.89 (0.6,1.18)
East Asia	1.31 (0.68,2.73)	0.16 (0.08,0.34)	4.96 (2.99,9.05)	0.25 (0.15,0.45)	1.64 (1.5,1.78)
Eastern Europe	0.39 (0.31,0.46)	0.14 (0.11,0.17)	1.28 (1.15,1.41)	0.37 (0.33,0.4)	3.33 (3.06,3.61)
Eastern Sub-Saharan Africa	0.01 (0,0.01)	0.01 (0,0.02)	0.02 (0.01,0.04)	0.02 (0.01,0.03)	1.54 (1.4,1.69)
High-income Asia Pacific	1.27 (1.1,1.57)	0.67 (0.58,0.83)	7.03 (5.79,7.93)	1.21 (1.01,1.36)	2.15 (2.05,2.24)
High-income North America	5.71 (4.94,6.62)	1.54 (1.33,1.78)	11.31 (9.82,12.31)	1.57 (1.38,1.71)	0.1 (0.01,0.19)
North Africa and Middle East	0.26 (0.12,0.59)	0.18 (0.08,0.41)	0.93 (0.55,1.91)	0.25 (0.15,0.51)	1.26 (1.13,1.39)
Oceania	0 (0,0)	0.04 (0.01,0.07)	0 (0,0.01)	0.04 (0.01,0.07)	0.38 (0.15,0.61)
South Asia	0.38 (0.14,0.78)	0.08 (0.03,0.17)	1.74 (1.03,3.15)	0.13 (0.08,0.24)	1.79 (1.64,1.94)
Southeast Asia	0.31 (0.19,0.77)	0.14 (0.08,0.34)	1.5 (1.03,2.92)	0.26 (0.19,0.5)	2.25 (2.2,2.3)
Southern Latin America	0.26 (0.22,0.32)	0.58 (0.49,0.72)	0.86 (0.75,0.95)	0.95 (0.84,1.05)	2.27 (2.06,2.48)
Southern Sub-Saharan Africa	0.13 (0.06,0.22)	0.52 (0.25,0.9)	0.3 (0.19,0.55)	0.6 (0.38,1.06)	0.55 (0.45,0.66)
Tropical Latin America	0.27 (0.25,0.31)	0.32 (0.28,0.37)	1.66 (1.48,1.79)	0.68 (0.6,0.73)	3.16 (2.94,3.37)
Western Europe	6.73 (5.68,8.31)	1.11 (0.93,1.36)	17.71 (15.03,19.62)	1.52 (1.31,1.67)	1.1 (0.99,1.21)
Western Sub-Saharan Africa	0.01 (0,0.01)	0.01 (0.01,0.02)	0.02 (0.01,0.03)	0.02 (0.01,0.02)	2.14 (1.76,2.52)

Deaths cases: number of cases/thousands; ASDR: age-standardized deaths rate per 100,000 persons;

ASR, age-standardized rate; SDI, socio-demographic index; GBD, global burden of diseases, injuries, and risk factors study; EAPC, estimated annual percentage change; CI, confidence interval; MDS/MPN, myelodysplastic syndromes/myeloproliferative neoplasms.

**Table 3:** The case number and ASR of DALYs of MDS/MPN in 1990 and 2021 by SDI quintiles and by GBD regions, with EAPC from 1990 to 2021.

Location	1990		2021		1990-2021
	Dalys cases (95% CI)	ASR of DALYs (95% CI)	Dalys cases (95% CI)	ASR of DALYs (95% CI)	EAPCs (95% CI)
Global	509.16 (422.11,662.98)	13.04 (10.95,16.67)	1214.11 (1041.72,1448.47)	14.56 (12.5,17.32)	0.46 (0.41,0.52)
SDI quintiles					
High SDI	279.94 (241.83,332.77)	25.66 (22.15,30.5)	591.15 (526.26,654.06)	27.97 (25.05,30.97)	0.3 (0.26,0.35)
High-middle SDI	118.85 (93.93,157.26)	12.09 (9.61,15.88)	306.54 (253.8,380.71)	16.58 (13.73,20.74)	1.14 (0.99,1.3)
Middle SDI	81.4 (61.12,130.52)	6.28 (4.72,10.1)	227.33 (182.63,325.79)	8.77 (7.06,12.55)	1.31 (1.22,1.39)
Low-middle SDI	24.32 (16.59,39.71)	3.09 (2.01,5.24)	76.4 (59.49,114.55)	4.98 (3.84,7.53)	1.89 (1.78,1.99)
Low SDI	4.11 (2.51,6.44)	1.34 (0.75,2.17)	11.37 (7.35,17.22)	1.8 (1.11,2.81)	1.28 (1.11,1.45)
GBD regions					
Andean Latin America	1.61 (1.2,2.26)	5.69 (4.2,8.19)	5.29 (4.18,7.17)	8.72 (6.89,11.77)	1.25 (0.76,1.75)
Australasia	7.59 (6.22,9.74)	32.38 (26.59,41.36)	15.48 (13.61,17.1)	27.15 (24.17,29.98)	-0.69 (-0.8,-0.58)
Caribbean	2.59 (2.11,3.54)	8.77 (7.25,11.82)	6.61 (5.6,8.08)	12.86 (10.88,15.82)	1.64 (1.48,1.79)
Central Asia	1.24 (0.91,1.73)	2.06 (1.52,2.78)	3.36 (2.81,3.92)	3.71 (3.11,4.32)	1.9 (1.71,2.1)
Central Europe	20.49 (16.45,25.33)	14.1 (11.31,17.44)	50.54 (45.35,56.96)	23.33 (20.94,26.52)	1.85 (1.63,2.07)
Central Latin America	21.56 (17.54,27.28)	17.11 (14.1,21.14)	63.69 (56.24,72.61)	25.58 (22.55,29.15)	1.61 (1.44,1.77)
Central Sub-Saharan Africa	0.15 (0.11,0.22)	0.46 (0.29,0.67)	0.39 (0.26,0.55)	0.52 (0.33,0.75)	0.43 (0.25,0.6)
East Asia	76.15 (50.12,128.66)	7.51 (4.96,12.62)	190.75 (133.28,297.37)	9.74 (6.81,15.39)	0.99 (0.89,1.09)
Eastern Europe	30.17 (22.02,40.26)	11.02 (8.1,14.66)	55.3 (44.19,68.75)	16.63 (13.34,20.57)	1.45 (1.34,1.57)
Eastern Sub-Saharan Africa	0.41 (0.26,0.61)	0.36 (0.2,0.55)	1.1 (0.7,1.53)	0.47 (0.28,0.67)	0.88 (0.79,0.98)
High-income Asia Pacific	41.88 (34.42,51.7)	21.84 (17.94,26.93)	131.27 (112.74,150.25)	30.35 (25.86,34.99)	1.15 (1.05,1.24)
High-income North America	113.33 (99.58,131.47)	31.86 (28.01,37.01)	207.75 (187.62,228.47)	31.77 (28.6,35.01)	-0.01 (-0.07,0.04)
North Africa and Middle East	10.34 (6.21,19.5)	4.83 (2.81,9.58)	26.83 (17.39,49.96)	5.83 (3.79,10.99)	0.78 (0.69,0.88)
Oceania	0.1 (0.05,0.15)	2.06 (1,3.03)	0.26 (0.12,0.39)	2.29 (1.13,3.33)	0.49 (0.38,0.6)
South Asia	21.49 (13.42,34.75)	2.93 (1.72,5)	67.2 (46.81,103.29)	4.27 (2.95,6.65)	1.46 (1.33,1.58)
Southeast Asia	10.72 (6.95,23.67)	3.6 (2.27,8.14)	41.15 (28.79,76.57)	6.34 (4.45,11.75)	1.98 (1.93,2.03)
Southern Latin America	6.61 (5.72,8.08)	14.17 (12.27,17.33)	18.68 (16.51,20.68)	22 (19.42,24.32)	1.98 (1.8,2.17)
Southern Sub-Saharan Africa	3.7 (2.08,5.95)	12.01 (6.34,19.73)	8.21 (5.25,15.11)	13.6 (8.89,24.63)	0.51 (0.4,0.61)
Tropical Latin America	8.78 (7.82,10.27)	8.03 (7.23,9.34)	38.97 (35.53,41.73)	15.73 (14.35,16.85)	2.8 (2.6,3.01)
Western Europe	129.66 (110.74,156.87)	22.26 (18.98,26.9)	279.78 (249.71,308.94)	27.98 (25.16,30.72)	0.75 (0.62,0.88)
Western Sub-Saharan Africa	0.58 (0.38,0.84)	0.51 (0.34,0.71)	1.47 (1,2.04)	0.62 (0.45,0.8)	0.69 (0.57,0.81)

DALYs cases: number of cases/thousands; ASR of DALYs: age-standardized DALYs rate per 100,000 persons;

DALYs, disability-adjusted life-years; ASR, age-standardized rate; SDI, socio-demographic index; GBD, global burden of diseases, injuries, and risk factors study; EAPC, estimated annual percentage change; CI, confidence interval; MDS/MPN, myelodysplastic syndromes/myeloproliferative neoplasms.

### 3.2 The trend of MDS/MPN burden from 1990 to 2021

From 1990 to 2021, the ASR for the incidence, deaths, and DALYs of MDS/MPN elevated by an average of 0.14% (95%CI: 0.068, 0.212), 0.698% (95%CI: 0.635, 0.76), and 0.464% (95%CI: 0.413, 0.515), respectively (**Tables 1**–**3**). Additionally, considering the range of SDI quintiles, the greatest rises in ASR of incidence were detached in middle SDI quintiles, whereas the most significant increases in deaths and DALYs were found in low-middle SDI quintiles. Throughout the five SDI quintiles from 1990 to 2021, an upward trend was evident in the ASR of incidence, deaths, and DALYs; however, the ASR of incidence within the high-middle SDI quintile seemed to remain stable during this timeframe. Regionally, tropical Latin America experienced the notable increases in both incidence and DALYs, while Eastern Europe saw the most considerable rise in deaths. In contrast, Oceania reported the most substantial decreases in incidence, deaths, and DALYs. At a national level, the overall patterns of disease burden showed considerable variation among 204 countries and territories. Greece reported the highest EAPC for incidence, deaths, and DALYs, while Bahrain had the lowest EAPC for these metrics (**Figure 2**; **Supplementary Tables S2–S5**).

### 3.3 Local trends in MDS/MPN burden using joinpoint regression analysis

**Figure 5:**
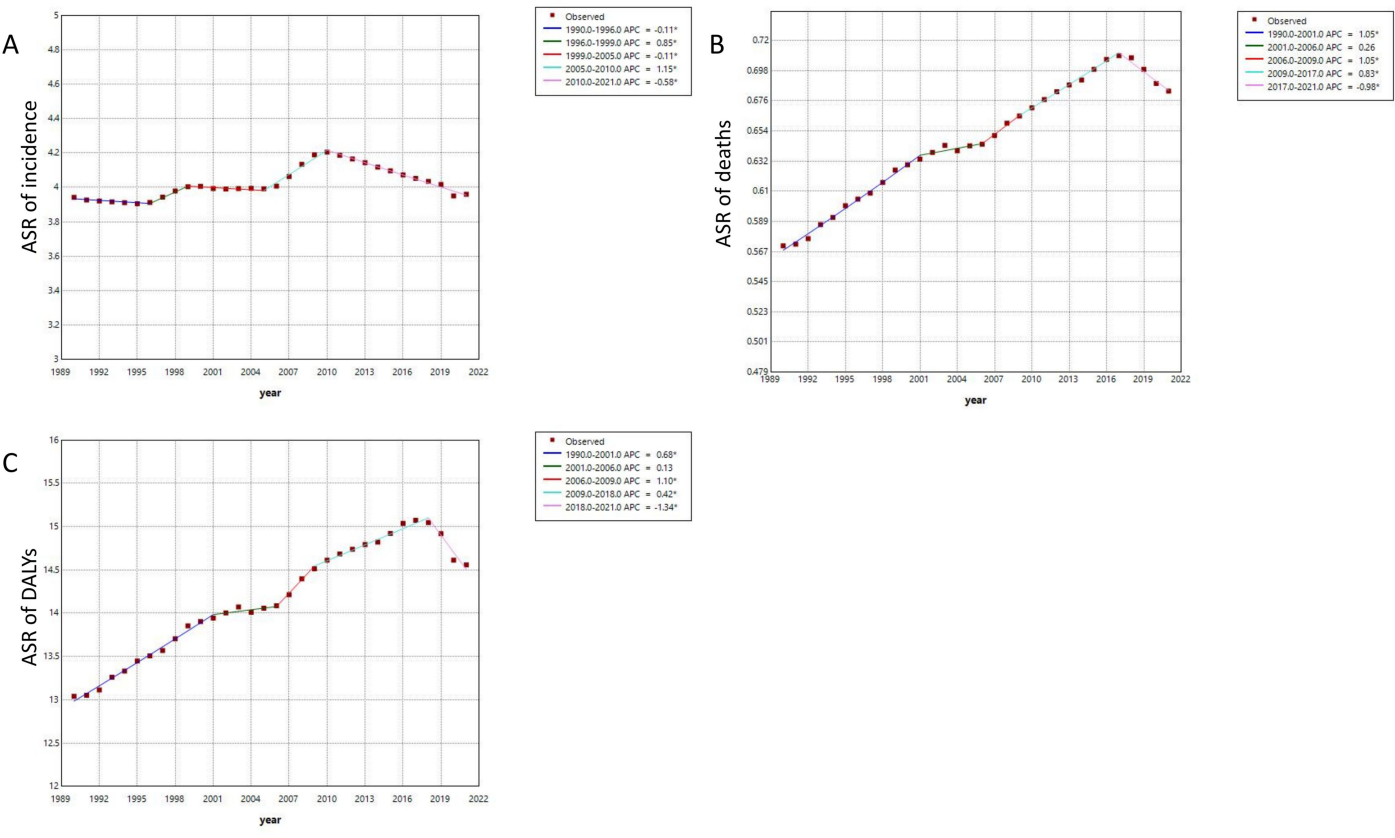
**(A)** The joinpoint regression analysis on the ASR of incidence; **(B)** The joinpoint regression analysis on the ASR of deaths; **(C)** The joinpoint regression analysis on the ASR of DALYs. ASR, age-standardized rate per 100,000 persons; DALYs, disability-adjusted life-years; SDI, socio-demographic index.

The findings from the joinpoint regression analysis regarding the burden of MDS/MPN are presented in **Figure 5**. From 1990 to 2021, the ASR illustrated an overall upward trend; however, local trends at various intervals exhibit inconsistency. Specifically, the trend of the ASR of incidence experienced two notable increases during the periods of 1996-1999 and 2005-2010, with the most rapid growth occurring between 2005 and 2010. Conversely, during the periods of 1990-1996, 1999-2005, and 2010-2021, a slight downward was observed. In terms of the ASR of deaths, from 1999 to 2017, it exhibited continuous growth at varying rates, with the most significant upward occurring between 1990-2001 and 2006-2009, followed by a downward trend from 2017 to 2021. Meanwhile, the trend of the ASR of DALYs also showed continuous growth at different rates from 1999 to 2018, with the fastest upward noted during 2006-2009. Finally, a clear downward trend was observed from 2018 to 2021.

### 3.4 Age-period-cohort analysis

**Figure 6:**
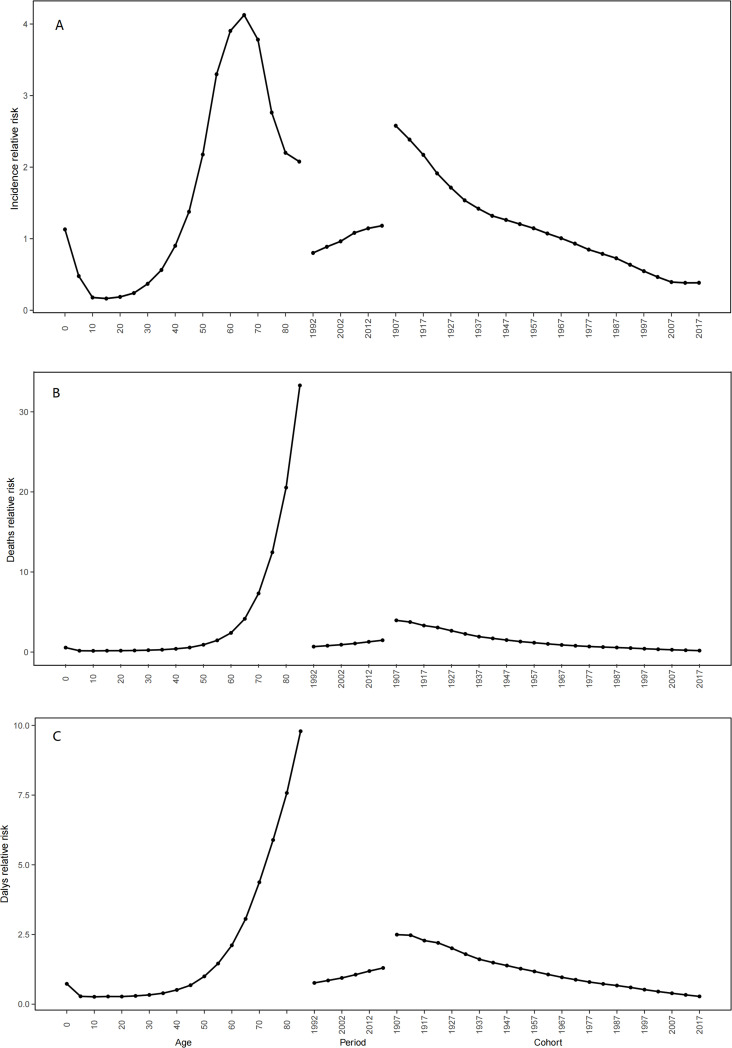
The effects of age, period, and birth cohort on the relative risk of MDS/MPN incidence **(A)**, deaths **(B)** and DALYs **(C)**. DALYs, disability-adjusted life-years; MDS/MPN, myelodysplastic syndromes/myeloproliferative neoplasms.

The findings from the age-period-cohort analysis concerning the incidence, deaths, and DALYs associated with MDS/MPN were depicted in **Figure 6**. Upon adjusting for effects related to period and birth cohort, the age effect displayed a profound influence on the likelihood of developing MDS/MPN. The incidence rate showed a trajectory that started with a decrease, followed by an increase, and then decreased once more, peaking in individuals aged 65 to 69 years. Trends in relative deaths and DALYs initially declined before rising again, with the greatest risk identified among those aged 85 and older. When age and birth cohort effects were accounted for, notable period effects on the risk of MDS/MPN incidence, deaths, and DALYs were detected. The period effect illustrated a modest upward trend in incidence, deaths, and DALYs, reporting RR increases of 1.47, 2.17, and 1.7 from the period of 1992 to 2017 as shown in **Table 4**. With age and period effects controlled, the birth cohort effect significantly influenced the risks associated with MDS/MPN incidence, deaths, and DALYs. This birth cohort effect indicated a higher risk for incidence, deaths, and DALYs in earlier birth cohorts in comparison to their later counterparts, with a continuous decline in RR from the 1907-1911 cohort to the 2017-2021 cohort (**Table 4**; **Supplementary Tables S6–S8**, **Supplementary Figure S1**).

**Table 4:** RR of MDS/MPN incidence, deaths and DALYs due to age, period, and birth cohort effects.

Factor	Incidence		Deaths		DALYs	
	RR (95%CI)	P	RR (95%CI)	P	RR (95%CI)	P
Age (years)						
0-4	1.13 (1.12~1.14)	<0.001	0.55 (0.54~0.57)	<0.001	0.73 (0.73~0.73)	<0.001
5-9	0.48 (0.48~0.48)	<0.001	0.16 (0.16~0.17)	<0.001	0.28 (0.28~0.28)	<0.001
10-14	0.18 (0.18~0.18)	<0.001	0.15 (0.15~0.16)	<0.001	0.26 (0.26~0.27)	<0.001
15-19	0.16 (0.16~0.16)	<0.001	0.17 (0.16~0.17)	<0.001	0.28 (0.27~0.28)	<0.001
20-24	0.19 (0.18~0.19)	<0.001	0.17 (0.17~0.18)	<0.001	0.27 (0.27~0.27)	<0.001
25-29	0.24 (0.24~0.24)	<0.001	0.2 (0.19~0.2)	<0.001	0.3 (0.3~0.3)	<0.001
30-34	0.37 (0.37~0.37)	<0.001	0.23 (0.23~0.24)	<0.001	0.33 (0.33~0.33)	<0.001
35-39	0.56 (0.56~0.57)	<0.001	0.29 (0.28~0.3)	<0.001	0.39 (0.39~0.4)	<0.001
40-44	0.9 (0.9~0.9)	<0.001	0.4 (0.4~0.41)	<0.001	0.51 (0.51~0.51)	<0.001
45-49	1.38 (1.37~1.38)	<0.001	0.57 (0.55~0.58)	<0.001	0.68 (0.68~0.68)	<0.001
50-54	2.18 (2.17~2.18)	<0.001	0.91 (0.9~0.93)	<0.001	0.99 (0.99~1)	0.04
55-59	3.3 (3.29~3.31)	<0.001	1.46 (1.44~1.48)	<0.001	1.46 (1.46~1.46)	<0.001
60-64	3.9 (3.89~3.91)	<0.001	2.39 (2.37~2.42)	<0.001	2.11 (2.11~2.11)	<0.001
65-69	4.13 (4.11~4.14)	<0.001	4.15 (4.11~4.19)	<0.001	3.06 (3.05~3.06)	<0.001
70-74	3.78 (3.77~3.79)	<0.001	7.32 (7.26~7.38)	<0.001	4.37 (4.37~4.38)	<0.001
75-79	2.76 (2.75~2.77)	<0.001	12.46 (12.35~12.57)	<0.001	5.89 (5.88~5.9)	<0.001
80-84	2.2 (2.19~2.21)	<0.001	20.54 (20.32~20.75)	<0.001	7.58 (7.56~7.59)	<0.001
85-89	2.08 (2.06~2.09)	<0.001	33.3 (32.88~33.73)	<0.001	9.79 (9.77~9.81)	<0.001
Period						
1992-1996	0.8 (0.8~0.8)	<0.001	0.68 (0.67~0.68)	<0.001	0.76 (0.76~0.76)	<0.001
1997-2001	0.89 (0.88~0.89)	<0.001	0.79 (0.79~0.8)	<0.001	0.85 (0.85~0.85)	<0.001
2002-2006	0.96 (0.96~0.96)	<0.001	0.92 (0.91~0.92)	<0.001	0.94 (0.94~0.94)	<0.001
2007-2011	1.08 (1.08~1.08)	<0.001	1.08 (1.07~1.08)	<0.001	1.06 (1.06~1.06)	<0.001
2012-2016	1.14 (1.14~1.15)	<0.001	1.28 (1.27~1.29)	<0.001	1.19 (1.19~1.19)	<0.001
2017-2021	1.18 (1.18~1.18)	<0.001	1.47 (1.46~1.49)	<0.001	1.3 (1.3~1.3)	<0.001
Birth cohort						
1907-1911	2.58 (2.54~2.62)	<0.001	3.96 (3.87~4.05)	<0.001	2.49 (2.48~2.51)	<0.001
1912-1916	2.38 (2.36~2.41)	<0.001	3.75 (3.69~3.81)	<0.001	2.47 (2.47~2.48)	<0.001
1917-1921	2.17 (2.16~2.19)	<0.001	3.31 (3.27~3.36)	<0.001	2.28 (2.28~2.29)	<0.001
1922-1926	1.91 (1.9~1.92)	<0.001	3.06 (3.03~3.1)	<0.001	2.2 (2.2~2.2)	<0.001
1927-1931	1.71 (1.71~1.72)	<0.001	2.66 (2.64~2.68)	<0.001	2.01 (2~2.01)	<0.001
1932-1936	1.54 (1.53~1.54)	<0.001	2.27 (2.25~2.28)	<0.001	1.79 (1.79~1.8)	<0.001
1937-1941	1.42 (1.42~1.42)	<0.001	1.92 (1.91~1.94)	<0.001	1.61 (1.61~1.61)	<0.001
1942-1946	1.32 (1.31~1.32)	<0.001	1.71 (1.69~1.72)	<0.001	1.49 (1.49~1.49)	<0.001
1947-1951	1.26 (1.26~1.27)	<0.001	1.5 (1.48~1.52)	<0.001	1.39 (1.38~1.39)	<0.001
1952-1956	1.2 (1.2~1.21)	<0.001	1.31 (1.29~1.33)	<0.001	1.27 (1.27~1.28)	<0.001
1957-1961	1.15 (1.14~1.15)	<0.001	1.17 (1.15~1.19)	<0.001	1.18 (1.17~1.18)	<0.001
1962-1966	1.07 (1.07~1.08)	<0.001	1.02 (1~1.04)	0.1	1.07 (1.06~1.07)	<0.001
1967-1971	1.01 (1~1.01)	<0.001	0.89 (0.87~0.91)	<0.001	0.96 (0.96~0.97)	<0.001
1972-1976	0.93 (0.93~0.94)	<0.001	0.78 (0.76~0.81)	<0.001	0.88 (0.87~0.88)	<0.001
1977-1981	0.85 (0.84~0.85)	<0.001	0.69 (0.67~0.72)	<0.001	0.79 (0.79~0.8)	<0.001
1982-1986	0.79 (0.78~0.79)	<0.001	0.62 (0.6~0.64)	<0.001	0.73 (0.72~0.73)	<0.001
1987-1991	0.73 (0.72~0.73)	<0.001	0.57 (0.55~0.59)	<0.001	0.67 (0.67~0.67)	<0.001
1992-1996	0.64 (0.63~0.64)	<0.001	0.5 (0.48~0.51)	<0.001	0.6 (0.6~0.6)	<0.001
1997-2001	0.55 (0.54~0.55)	<0.001	0.42 (0.4~0.43)	<0.001	0.52 (0.52~0.52)	<0.001
2002-2006	0.46 (0.46~0.47)	<0.001	0.35 (0.33~0.36)	<0.001	0.45 (0.45~0.46)	<0.001
2007-2011	0.39 (0.39~0.4)	<0.001	0.29 (0.27~0.3)	<0.001	0.39 (0.39~0.39)	<0.001
2012-2016	0.38 (0.38~0.39)	<0.001	0.23 (0.22~0.24)	<0.001	0.33 (0.33~0.34)	<0.001
2017-2021	0.38 (0.38~0.39)	<0.001	0.18 (0.17~0.19)	<0.001	0.28 (0.28~0.28)	<0.001

DALYs, disability-adjusted life-years; RR, relative risk; CI, confidence interval; MDS/MPN, myelodysplastic syndromes/myeloproliferative neoplasms.

### 3.5 Cross-country inequality analysis

**Figure 7:**
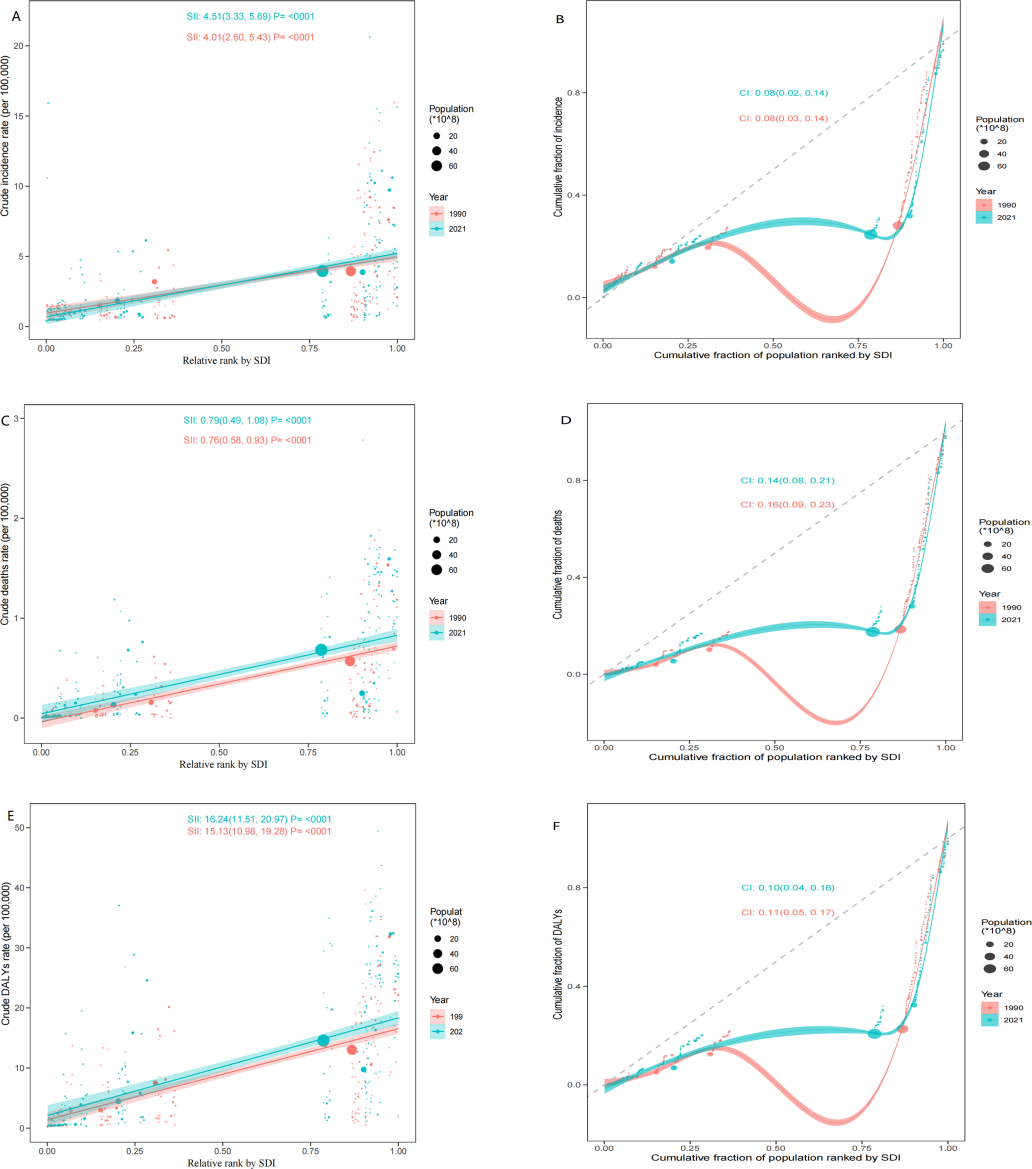
SDI-related health inequality regression analyses and concentration curves for the incidence **(A, D)**, deaths **(B, E)**, and DALYs **(C, F)** of MDS/MPN globally in 1990 and 2021. SDI, socio-demographic index; DALYs, disability-adjusted life-years; MDS/MPN, myelodysplastic syndromes/myeloproliferative neoplasms.

Significant absolute and relative SDI-associated inequalities associated with SDI in MDS/MPN burden were depicted in **Figure 7**. These inequalities have persisted over time and are notably concentrated in countries with higher levels of socio-demographic development. The inequality slope index indicated that the DALYs per 100,000 persons in individuals in countries at both the highest and lowest SDI exceeded 15.13 (95%CI: 10.98, 19.28) in 1990, rising to 16.24 (95%CI: 11.51, 20.97) by 2021. Additionally, the concentration index, which assessed relative gradient inequality, was recorded at 0.11 (95%CI: 0.05, 0.17) in 1990 and 0.10 (95%CI: 0.04, 0.16) in 2021, reflecting an uneven distribution of the burden across countries with varying SDI levels.

### 3.6 Decomposition analysis on MDS/MPN DALYs

**Figure 8:**
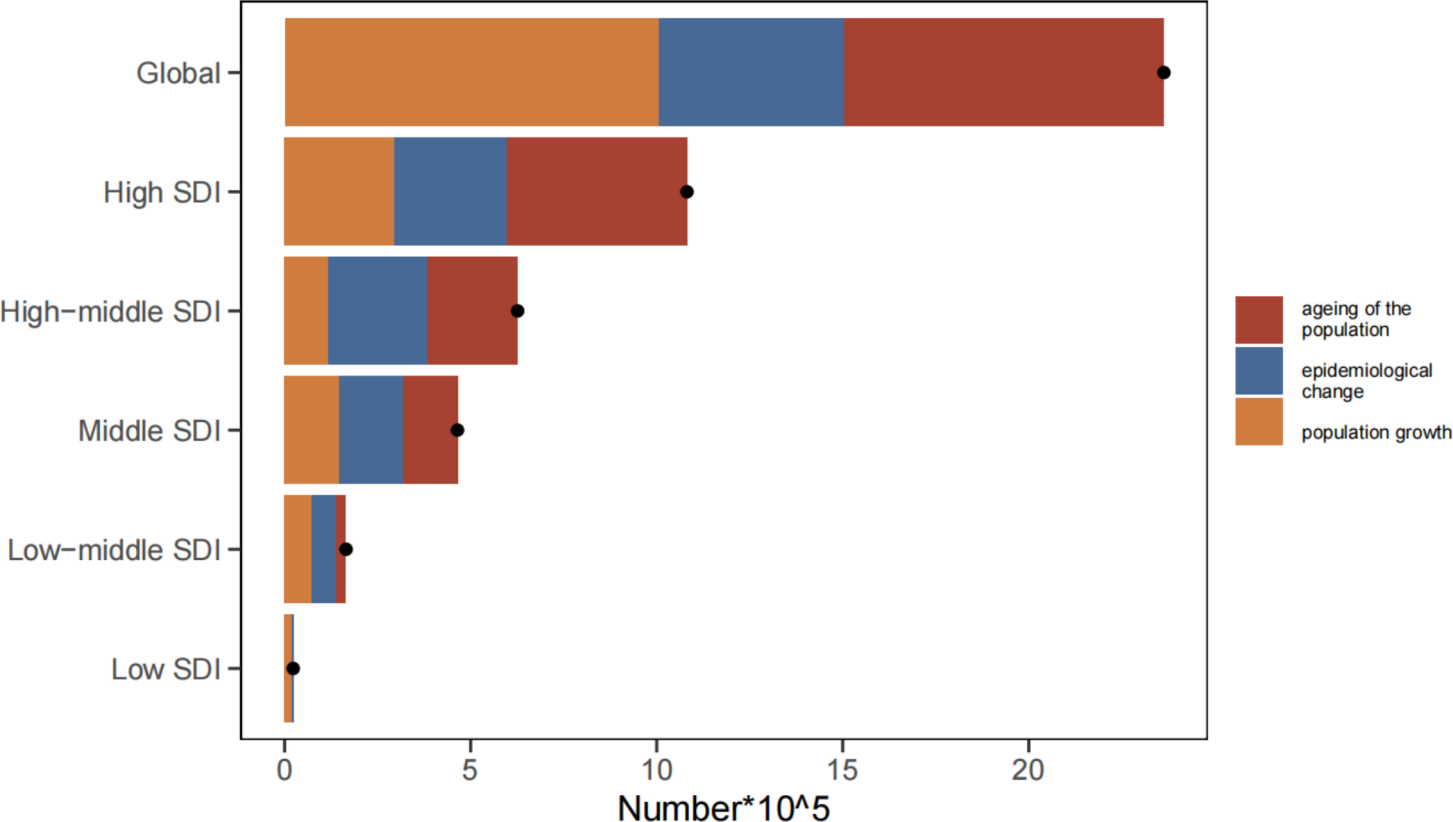
Changes in DALYs of MDS/MPN according to aging, population growth and epidemiological change from 1990 to 2021 at global level by SDI quintile. The black dot denotes the overall value of the change resulting from all three components. For each component, the magnitude of a positive value suggests a corresponding increase in MDS/MPN DALYs attributed to the component; the magnitude of a negative value suggests a corresponding decrease in MDS/MPN DALYs attributed to the component. DALYs, disability-adjusted life-years; SDI, socio-demographic index; MDS/MPN: myelodysplastic syndromes/myeloproliferative neoplasms; age, ageing of the population; asr: epidemiological change; population, population growth.

Over the past 30 years, DALYs have increased dramatically on a global scale, with the most substantial increases observed in high SDI quintiles (**Figure 8**, **Supplementary Table S9**). The global increase in DALYs can be attributed to aging (36.39%), population growth (42.61%), and epidemiological changes (21%). Notably, the most significant contributions from these factors were seen in the high SDI quintile (44.91%), low-middle SDI quintile (78.68%), and high-middle SDI quintile (42.38%), respectively.

### 3.7 Predictive analysis on MDS/MPN burden to 2045

**Figure 9:**
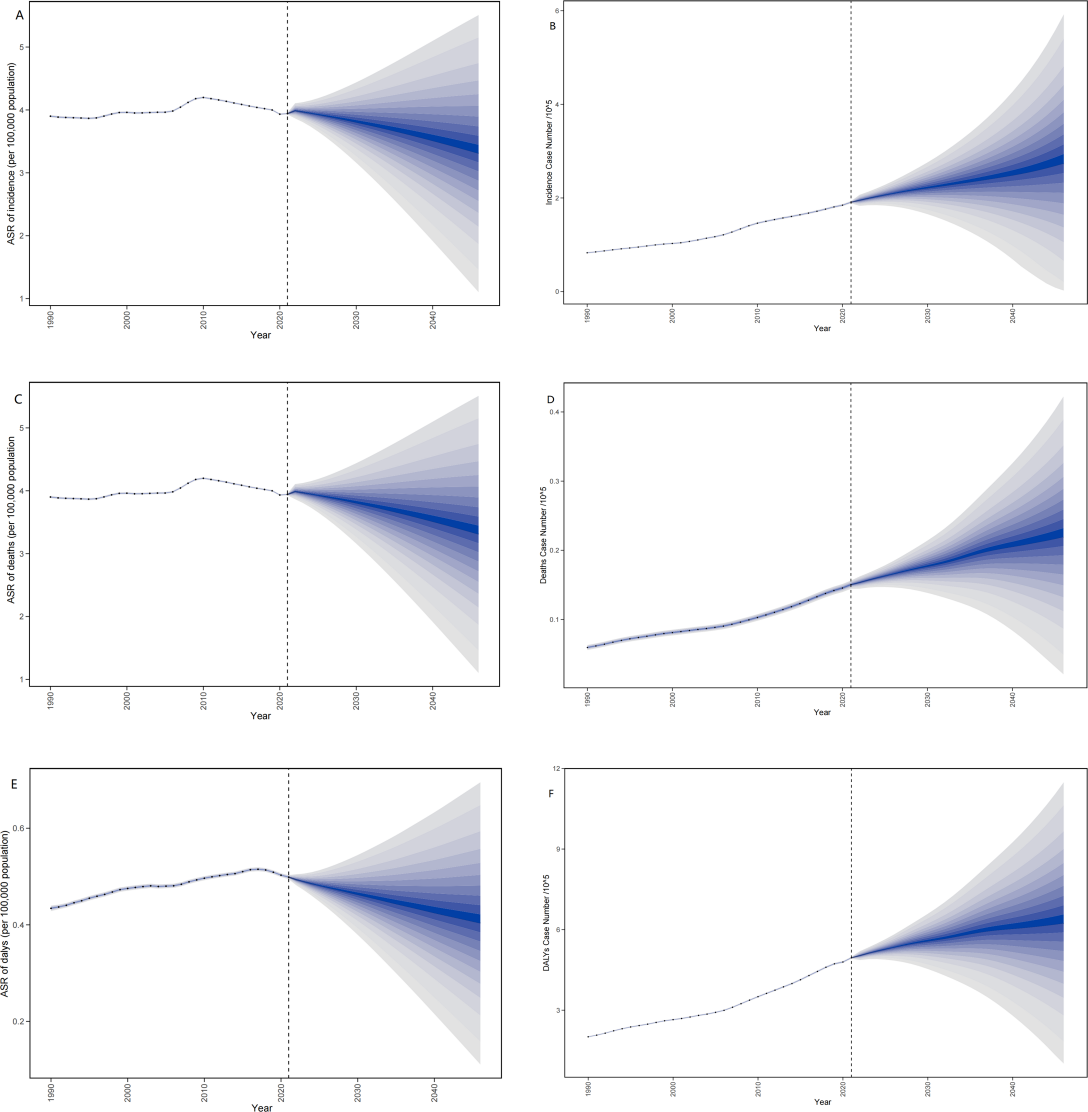
**(A)** The predicted case number of incidence to 2045; **(B)** The predicted ASR of incidence to 2045; **(C)** The predicted case number of deaths to 2045; **(D)** The predicted ASR of deaths to 2045; **(E)** The predicted case number of DALYs to 2045; **(F)** The predicted ASR of DALYs to 2045. ASR, age-standardized rate per 100,000 persons; DALYs, disability-adjusted life-years; MDS/MPN, myelodysplastic syndromes/myeloproliferative neoplasms.

**Figure 9** presented the projected number of cases, as well as the incidence, deaths, and DALYs associated with ASR up to the year 2045. It is anticipated that the global numbers for cases, incidence, deaths, and DALYs will increase. Conversely, the ASR for incidence, deaths, and DALYs is expected to decline by 2045. For more specific information about case numbers and the ASR related to incidence, deaths, and DALYs, refer to **Supplementary Table S10**.

## 4 Discussion

This research delivered the latest information regarding the incidence, deaths, and DALYs associated with MDS/MPN on a global, regional, and national scale from 1990 to 2021, and also provided an extensive evaluation that focused on inequality and predictive analyses. While discrepancies were observed in the incidence, deaths rates, and DALYs of MDS/MPN among different nations, there was an overall increase in the global burden of MDS/MPN from 1990 to 2021, with a notably slower rate of growth after 2018. Additionally, the burden was found to be significantly greater in men compared to women. Decomposition analysis identified population growth as a key factor influencing the variations in the burden of MDS/MPN. An inequality analysis across countries indicated that high SDI countries bore a disproportionate share of the MDS/MPN burden, with SDI-related disparities persisting. Importantly, although the ASR for incidence, deaths, and DALYs were forecasted to have a slight annual decline from 2020 to 2045, the absolute number of these metrics is anticipated to continue rising, pointing to a significant challenge in the management and control of MDS/MPN in the upcoming decades.

From 1990 to 2021, the number of incidence cases of MDS/MPN increased significantly, rising from 171,132 to 341,017 globally. Furthermore, our research identified overall increasing trends in the incidence, deaths, and DALYs associated with MDS/MPN from 1990 to 2021, indicating that the global burden of MDS/MPN has escalated over the years. Notably, between 1990 and 2021, only Oceania exhibited a decreasing trend in incidence, deaths, and DALYs, while other regions experienced stability or increases. This trend may be attributed to advancements in medical policies and heightened health awareness in Oceania. One previous study (37) indicated a 24.6% reduction in all-cause DALYs in Australia from 1990 to 2021, which serves as a valuable reference for other countries and regions. Furthermore, a statistical study conducted between 2005 and 2007 reported age-standardized incidence rates of MDS in Australia and New Zealand at 3.2 and 3.7 cases per 100,000 persons, respectively (38). Our data exceed these cases, as our study also encompassed bone marrow diseases beyond MDS.

In 2021, high-income North America had the highest burden of MDS/MPN disease; however, this burden has exhibited a decreasing trend since 1990. The United States, as a main country in this context, has maintained stable DALYs between 1990 and 2021, despite high levels ASR of incidence, deaths and DALYs. This may be attributed to the advanced medical technology and ample research funding available in the United States, which offers valuable insights for global health, particularly for developing countries with limited resources. Similarly, previous studies have indicated that the incidence of MDS in Asia is lower than that in Western countries (20). Notably, China, as a middle SDI country, reported the highest number of MDS/MPN cases, likely due to its large population. Furthermore, the EAPC reflected an increasing trend in incidence, deaths, and DALYs. An epidemiological study of MDS in Asia has revealed that Asian populations are more prone to developing high-risk MDS, with a higher proportion of Asian MDS patients exhibiting high-risk cytogenetic aberrations. Collectively, these factors present an unavoidable and formidable challenge to the management of MDS. Tropical Latin America has experienced the fastest growing incidence and DALYs from 1990 to 2021, indicating a need for improved management through effective programs for the early diagnosis, management, and treatment of MDS/MPN. Furthermore, men globally exhibited significantly higher incidence, deaths and DALYs compared to women, which may be associated with harmful exposures such as smoking (39) and various occupational factors (40–43). A study conducted in China reported a higher prevalence of women (44); however, contrary to previous findings, our research indicated Chinese men also experienced significantly higher incidence, deaths and DALYs than their women counterparts. The disparities in the burden of MDS/MPN and its trends across different countries suggest that flexible health policies should be tailored to the specific conditions of each country and region.

When dividing the overall trend into multiple time periods, we were surprised to find that the incidence rates exhibited fluctuations and slight increases from 1990 to 2021, peaking in 2010. This peak may be associated with the evolving diagnostic criteria for MDS/MPN. The 2008 WHO classification provided a more detailed categorization of MDS/MPN (45), emphasizing the importance of morphological features and incorporating molecular genetic data into the diagnostic criteria, thereby rendering the reported incidence of the disease a dynamic and evolving target (6). The increase in MDS/MPN incidence observed from 2005 to 2010 may be attributed to enhanced awareness of reporting practices and improved care for older adults, rather than a genuine rise in disease risk. Furthermore, the increasing trend in deaths and burden from 1990 to 2017, along with the deceleration of this increase from 2018 to 2021, was likely related to advancements in medical technology and the ongoing enhancement of MDS/MPN treatments (46), such as the discovery and utilization of new methylating agents and the continuous introduction of novel drugs (47), such as luspatercept (48), eltanexor (49), the JAK inhibitor Ruxolitinib (50) and Jaktinib (51).

Previous descriptive studies have primarily demonstrated trends in risk associated with age in a cross-sectional manner, without controlling for other confounding factors (52). Conversely, the current research utilized an age-period-cohort framework to examine the distinct impact of age on risk. Regarding the age effect, an increase in the relative risk of incidence was observed to increase with age, reaching its highest point in the 65-69 years age cohort, after which a decrease occurred. Consequently, it is typically advised that people in this age category be provided with more focused early diagnosis, management, and treatment strategies. Similarly, a previous study using the data from SEER (22) reported an age-adjusted incidence rate of MDS of 4.8 cases per 100,000 persons, with a significantly higher annual incidence rate of 55.5 cases per 100,000 persons among patients aged over 80 years. Furthermore, the relative risk of incidence for individuals over 40 years of age exceeded 1 for both men and women, indicating that there may be a need to intensify health education programs related to MDS/MPN starting from the age of 40. Additionally, both relative deaths risk and disease burden increased with age, reaching their highest levels in the 85+ age group, thereby reinforcing the role of aging as a significant risk factor for MDS/MPN (53). As for the period effect, relative incidence, deaths, and risk of burden were found to increase as the period progressed, which may be tightly related to the gradually increased risk factors for MDS/MPN, particularly within the aging population (17). Additionally, since the estimates in GBD 2021 were derived from the most recent epidemiological data, it is plausible that a progressively more sophisticated health registry system over time may also partly account for the observed increase in MDS/MPN burden. In relation to the birth cohort effect, a trend of decreasing relative incidence, deaths, and DALY risks was noted as cohorts age, suggesting that individuals born earlier are at a greater risk for MDS/MPN compared to those born later. Research indicated that specific populations exposed to chemical agents, radiation, or particular medications had a notably elevated risk of MDS when exposure occurred at younger ages, pointing to the existence of a cohort effect (54). Additionally, individuals born more recently seem to have enhanced access to health education and improved healthcare resources relative to those born in earlier generations, which supported findings from a previous GBD study in Spain (21).

We examined the relationship between incidence, deaths, and DALYs for MDS/MPN and SDI. It is generally assumed that countries with high levels of SDI have greater access to and better-performing healthcare systems, which may result in lower disease burdens. However, a disproportionate burden of MDS/MPN has been observed in countries with high levels of socio-demographic development, where the highest number of cases and ASR for incidence, deaths, and DALYs were found in quintiles with elevated SDI. The inequality of MDS/MPN burden concentrating in high SDI countries may be mainly attributed to two aspects. For one, these countries possessed advanced medical technology and diagnostic capabilities, leading to higher rates of definitive MDS/MPN diagnoses. For instance, the United States has implemented a comprehensive diagnostic approach for MDS/MPN, incorporating advanced techniques such as cytomorphological analysis, cytogenetic testing, and molecular biological assessments. For another, high SDI levels often correlated with an aging population, which represented another significant risk factor for MDS/MPN and contributes to its increased incidence. Notably, these inequalities have persisted over time, indicating that more resources should be allocated to the early diagnosis, management, and treatment of MDS/MPN as socio-demographic development levels rise. There are significant differences in healthcare infrastructure across the globe. High-income countries possess a well-developed healthcare infrastructure, characterized by advanced medical equipment and a sufficient number of medical professionals, allowing patients better access to sophisticated diagnostic and therapeutic tools. In Oceania, the Healthcare Acquisition and Quality (HAQ) Index, as reported by the Lancet, indicates that the region’s healthcare infrastructure is highly developed, with a HAQ value reaching as high as 90 (55). Concurrently, the disease burden associated with MDS/MPN has decreased over recent years. In contrast, the HAQ index in tropical Latin America is approximately 60, suggesting that access to and quality of health care in the region are at a moderate level. However, it is concerning that the burden of MDS/MPN has increased the most rapidly in this region over the past period. This trend underscores the challenges associated with managing MDS/MPN diseases in the region, indicating a need for heightened attention and targeted interventions. To mitigate these disparities, targeted policies should be implemented in different countries to rationalize the allocation of healthcare resources.

Our study identified the most rapid increase in burden occurring in low-middle SDI regions. The decomposition analysis of DALYs indicated that the global rise in burden was predominantly influenced by population growth and aging. Specifically, the increase in burden within high SDI regions was primarily attributed to population aging, whereas population growth significantly impacted low SDI regions. In low-middle SDI regions, the rise in burden was chiefly driven by population growth. For instance, according to the World Bank (56), India’s total population in 1990 was 870.45 million, and by 2021, it had increased to 1,407.56 lakhs. This represented an approximate 61.67% increase in India’s population from 1990 to 2021, which partially accounted for the rising burden. These findings suggested that varying demographic factors contribute to distinct patterns of change in MDS/MPN burden relative to SDI. Consequently, there was an urgent need to enhance efforts for the early diagnosis, and treatment of MDS/MPN in low-middle SDI regions moving forward.

Importantly, while the ASR for incidence, deaths, and DALYs associated with MDS/MPN are projected to decline annually until 2045, the absolute number of cases for these three indicators is expected to rise. This trend indicates a substantial disease burden and forthcoming challenges in the management and control of MDS/MPN. Considering the persistent high global burden of MDS/MPN, which currently lacks a specific cure, combined with the anticipated increase in case numbers worldwide, the need for effective prevention and management programs is urgent. Immediate action should focus on identifying high-risk groups, such as individuals aged 65 to 69, as highlighted in both current and prior research, with a particular emphasis on addressable risk factors, notably among smokers. To effectively reduce the global impact of MDS/MPN and enhance patient survival and quality of life, several strategies should be implemented. First, the promotion of precise diagnostic methods based on molecular typing, including genetic testing and next-generation sequencing technologies, is essential to improve the diagnostic accuracy of MDS/MPN and to facilitate individualized therapeutic effects. Second, international multicenter clinical trials should be encouraged to collect real-world data from diverse countries and regions, thereby evaluating the treatment patterns and outcomes associated with MDS/MPN (57). Finally, public health initiatives should aim to raise awareness of MDS/MPN and to strengthen the disease management capabilities of patients and their families.

This study has several limitations. Firstly, while the GBD database encompasses data from numerous countries and regions worldwide, certain areas still experience issues with missing data, which may introduce bias into the analysis results. Secondly, due to the scarcity of reliable global survival data, survival rates must be estimated using the deaths-to-incidence ratio, which serves as an alternative indicator of survival. However, this approach results in variations in data quality, leading to potential bias in the survival rates represented in the database, particularly favoring patients with longer survival. Consequently, the disease burden derived from GBD data may be underestimated. Thirdly, considering that MDS/MPN are relatively rare hematologic diseases, the GBD database does not categorize these diseases separately, thus preventing a distinct analysis of these two groups. Moreover, the GBD database lacks detailed molecular-level information, which restricts the potential for in-depth analysis of disease burden. Nevertheless, in comparison to other sources, the data provided by GBD remains the most comprehensive and standardized, establishing it as a crucial resource for investigating global health issues and an essential tool for global health research and policy development. To address the limitations of the GBD databases, we advocate for the development and implementation of diverse analytical methods to enhance and validate the findings of this study.

## 5 Conclusion

As a major public health concern, the global burden of MDS/MPN showed a general upward trend from 1990 to 2021, primarily attributed to population growth and aging. The greatest proportion of the MDS/MPN burden was observed predominantly among men, especially within older age groups. Nations with higher SDI levels experienced a substantially greater burden of MDS/MPN. While the burden of MDS/MPN was most pronounced in nations within the high SDI quintile, it is increasing most rapidly in low-middle SDI quintile, notably in tropical Latin America. Therefore, enhance attention to this disease is warranted, alongside the implementation of timely early diagnosis and control measures. This study highlights substantial challenges in the management and control of MDS/MPN, including a rising incidence of cases and global disparities. These findings may offer crucial insights for improving public health policies and ensuring the equitable distribution of medical resources.

## Data Availability

The original contributions presented in the study are included in the article/**Supplementary Material**. Further inquiries can be directed to the corresponding author. Data used for the analyses are publicly available from the Institute of Health Metrics and Evaluation (http://www.healthdata.org/; http://ghdx.healthdata.org/gbd-results-tool).
