# Supplementary material for: Global, regional, and national burden of myelodysplastic syndromes and myeloproliferative neoplasms, 1990-2021: an analysis from the global burden of disease study 2021
Source: Front Oncol. 2025 Mar 18;15:1559382. doi: 10.3389/fonc.2025.1559382 (PMC11958949; doi:10.3389/fonc.2025.1559382)
Supplement: Supplementary file 1 [file Table1.docx]

**Supplementary Material**

**Contents**

**Table S1……………………………………………………………………………………. 2**

**Table S2……………………………………………………………………………………. 3**

**Table S3……………………………………………………………………………………. 11**

**Table S4……………………………………………………………………………………. 19**

**Figure S1……………………………………………………………………………………29**

**Table S5……………………………………………………………………………………. 30**

**Table S6……………………………………………………………………………………. 31**

**Table S7……………………………………………………………………………………. 33**

**Table S8……………………………………………………………………………………. 35**

**Table S9……………………………………………………………………………………. 37**

**Table S10……………………………………………………………………………………38**

**Table S1 GBD 21 geographic regions corresponding to 7 continents**

| **7 Continents** | **GBD 21 geographic regions** |
| --- | --- |
| Asia | Central Asia, South Asia, Southeast Asia, East Asia, High-Income Asia Pacific (5) |
| Africa | Sub-Saharan Africa's Central, Eastern, Southern, and Western parts, along with North Africa and the Middle East (5) |
| Europe | Western Europe, Central Europe, Eastern Europe (3) |
| North America | High-Income North America, the Caribbean (2) |
| South America | Andean Latin America, Tropical Latin America, Central Latin America, Southern Latin America (4) |
| Oceania | Oceania, Australasia (2) |
| Antarctica | none |

**Table S2 The case number and ASR of incidence of MDS/MPN in 1990 and 2021 by 204 countries, with EAPC from 1990 to 2021**

|  | **1990** | | **2021** | | **1990-2021** | |  |
| --- | --- | --- | --- | --- | --- | --- | --- |
| **location** | **Incidence cases (95% CI)** | **ASIR(95% CI)** | **Incidence cases (95% CI)** | **ASIR (95% CI)** | **EAPCs(95% CI)** | |  |
| American Samoa | 0(0,0) | 1.73(1.49,2.03) | 0(0,0) | 2.06(1.8,2.37) | | 0.67(0.52,0.83) | |
| Antigua and Barbuda | 0(0,0) | 1.19(1,1.4) | 0(0,0) | 1.38(1.18,1.63) | | 0.57(0.53,0.61) | |
| Arab Republic of Egypt | 0.38(0.31,0.46) | 0.86(0.71,1.01) | 0.76(0.64,0.89) | 0.83(0.72,0.96) | | -0.07(-0.12,-0.03) | |
| Argentine Republic | 0.73(0.6,0.89) | 2.22(1.81,2.7) | 1.5(1.25,1.75) | 2.81(2.34,3.28) | | 1.3(0.92,1.68) | |
| Australia | 1.16(0.92,1.45) | 5.77(4.62,7.15) | 2.73(2.19,3.37) | 5.99(4.88,7.36) | | 0.1(0.06,0.14) | |
| Barbados | 0(0,0) | 1.58(1.32,1.91) | 0.01(0.01,0.01) | 1.81(1.53,2.16) | | 0.49(0.46,0.52) | |
| Belize | 0(0,0) | 1.36(1.15,1.63) | 0.01(0,0.01) | 1.6(1.35,1.91) | | 0.56(0.54,0.59) | |
| Bermuda | 0(0,0) | 2.05(1.7,2.47) | 0(0,0) | 2.81(2.39,3.32) | | 1.14(1.07,1.21) | |
| Bolivarian Republic of Venezuela | 0.53(0.45,0.64) | 4.37(3.63,5.33) | 1.65(1.38,1.95) | 5.35(4.52,6.26) | | 0.83(0.77,0.89) | |
| Bosnia and Herzegovina | 0.23(0.19,0.29) | 5.17(4.23,6.23) | 0.3(0.24,0.36) | 5.7(4.64,6.77) | | 0.38(0.34,0.41) | |
| Brunei Darussalam | 0.01(0.01,0.02) | 9.48(7.69,11.64) | 0.04(0.03,0.05) | 9.42(7.78,11.41) | | 0.03(0.01,0.05) | |
| Burkina Faso | 0.08(0.06,0.1) | 1.54(1.06,1.9) | 0.15(0.12,0.18) | 1.27(0.97,1.53) | | -0.61(-0.78,-0.43) | |
| Canada | 2.35(1.92,2.87) | 7.41(6.09,9.04) | 4.65(3.76,5.63) | 7.27(5.99,8.73) | | -0.08(-0.08,-0.07) | |
| Central African Republic | 0.01(0.01,0.01) | 0.55(0.45,0.65) | 0.02(0.01,0.02) | 0.51(0.44,0.61) | | -0.24(-0.28,-0.2) | |
| Commonwealth of Dominica | 0(0,0) | 1.87(1.58,2.21) | 0(0,0) | 2.5(2.16,2.9) | | 1.05(0.94,1.17) | |
| Commonwealth of the Bahamas | 0(0,0) | 1.93(1.63,2.3) | 0.01(0.01,0.01) | 2.33(1.99,2.72) | | 0.69(0.61,0.77) | |
| Cook Islands | 0(0,0) | 1.74(1.48,2.05) | 0(0,0) | 1.84(1.6,2.11) | | 0.18(0.11,0.25) | |
| Czech Republic | 0.6(0.48,0.72) | 4.65(3.81,5.61) | 1.11(0.94,1.29) | 6.17(5.23,7.09) | | 1.42(1.02,1.82) | |
| Democratic People's Republic of Korea | 0.38(0.3,0.48) | 1.89(1.51,2.36) | 0.63(0.5,0.79) | 1.88(1.5,2.33) | | -0.02(-0.04,0.01) | |
| Democratic Republic of Sao Tome and Principe | 0(0,0) | 1.57(1.04,1.96) | 0(0,0) | 1.27(0.99,1.56) | | -0.56(-0.71,-0.42) | |
| Democratic Republic of the Congo | 0.14(0.1,0.18) | 0.51(0.42,0.6) | 0.28(0.22,0.35) | 0.49(0.42,0.57) | | -0.11(-0.15,-0.06) | |
| Democratic Republic of Timor-Leste | 0(0,0) | 0.71(0.59,0.84) | 0.01(0.01,0.01) | 0.84(0.72,0.97) | | 0.65(0.6,0.69) | |
| Democratic Socialist Republic of Sri Lanka | 0.09(0.07,0.11) | 0.7(0.59,0.83) | 0.2(0.17,0.24) | 0.75(0.64,0.88) | | 0.27(0.24,0.31) | |
| Dominican Republic | 0.08(0.07,0.1) | 1.49(1.25,1.76) | 0.17(0.15,0.21) | 1.68(1.43,2) | | 0.46(0.43,0.48) | |
| Eastern Republic of Uruguay | 0.11(0.09,0.13) | 2.82(2.37,3.4) | 0.18(0.15,0.22) | 3.78(3.2,4.44) | | 1.15(1.08,1.21) | |
| Federal Democratic Republic of Ethiopia | 0.17(0.13,0.23) | 0.5(0.41,0.61) | 0.33(0.27,0.41) | 0.51(0.43,0.6) | | 0.1(0.07,0.13) | |
| Federal Democratic Republic of Nepal | 0.09(0.07,0.11) | 0.57(0.48,0.68) | 0.18(0.16,0.21) | 0.71(0.62,0.81) | | 1(0.86,1.13) | |
| Federal Republic of Germany | 2.53(2,3.48) | 2.09(1.65,2.87) | 4.85(3.93,5.93) | 2.78(2.26,3.41) | | 1.77(1.25,2.3) | |
| Federal Republic of Nigeria | 0.6(0.48,0.72) | 1.09(0.86,1.33) | 1.14(0.96,1.35) | 0.97(0.83,1.12) | | -0.29(-0.5,-0.08) | |
| Federal Republic of Somalia | 0.02(0.01,0.03) | 0.43(0.35,0.53) | 0.05(0.04,0.07) | 0.4(0.34,0.48) | | -0.2(-0.25,-0.15) | |
| Federated States of Micronesia | 0(0,0) | 1.71(1.46,2.03) | 0(0,0) | 1.98(1.73,2.27) | | 0.48(0.38,0.58) | |
| Federative Republic of Brazil | 0.62(0.52,0.75) | 0.62(0.5,0.75) | 4.73(3.84,5.7) | 1.91(1.57,2.29) | | 4.55(4.23,4.88) | |
| French Republic | 2.84(2.23,3.61) | 3.63(2.87,4.59) | 5.42(4.22,6.69) | 4.28(3.36,5.26) | | 0.45(0.34,0.55) | |
| Gabonese Republic | 0(0,0.01) | 0.63(0.54,0.74) | 0.01(0.01,0.01) | 0.71(0.63,0.8) | | 0.4(0.37,0.43) | |
| Georgia | 0.04(0.03,0.05) | 0.64(0.54,0.77) | 0.03(0.03,0.04) | 0.63(0.54,0.73) | | 0.08(-0.05,0.2) | |
| Grand Duchy of Luxembourg | 0.02(0.02,0.02) | 3.71(3.06,4.49) | 0.04(0.04,0.05) | 4.48(3.71,5.43) | | 0.38(-0.14,0.9) | |
| Greenland | 0(0,0) | 5.02(4.17,6.05) | 0(0,0) | 5.62(4.65,6.61) | | 0.38(0.31,0.46) | |
| Grenada | 0(0,0) | 1.97(1.65,2.37) | 0(0,0) | 2.66(2.33,3.01) | | 1(0.91,1.1) | |
| Guam | 0(0,0) | 1.71(1.48,2.02) | 0(0,0) | 1.81(1.58,2.09) | | 0.17(0.02,0.32) | |
| Hashemite Kingdom of Jordan | 0.01(0.01,0.01) | 0.4(0.33,0.49) | 0.04(0.04,0.05) | 0.46(0.39,0.55) | | 0.6(0.52,0.68) | |
| Hellenic Republic | 1.28(0.99,1.6) | 8.48(6.55,10.58) | 3.58(2.97,4.27) | 20.62(16.9,24.65) | | 4.79(4,5.59) | |
| Hungary | 1.23(1.02,1.48) | 8.86(7.38,10.58) | 1.56(1.29,1.86) | 9.64(8.1,11.35) | | 0.29(0.27,0.31) | |
| Independent State of Papua New Guinea | 0.05(0.04,0.06) | 1.49(1.26,1.76) | 0.14(0.12,0.16) | 1.64(1.44,1.9) | | 0.33(0.3,0.36) | |
| Independent State of Samoa | 0(0,0) | 1.74(1.48,2.06) | 0(0,0) | 2.08(1.83,2.38) | | 0.62(0.53,0.72) | |
| Ireland | 0.18(0.15,0.22) | 4.35(3.64,5.17) | 0.41(0.34,0.47) | 5.25(4.43,6.01) | | 0.6(0.57,0.62) | |
| Islamic Republic of Afghanistan | 0.08(0.07,0.1) | 0.9(0.75,1.09) | 0.18(0.14,0.22) | 0.74(0.62,0.86) | | -0.65(-0.71,-0.58) | |
| Islamic Republic of Iran | 0.47(0.39,0.56) | 0.98(0.81,1.17) | 0.76(0.61,0.93) | 0.85(0.72,1.02) | | -0.66(-1.03,-0.28) | |
| Islamic Republic of Mauritania | 0.02(0.01,0.02) | 1.56(1.08,1.92) | 0.03(0.02,0.04) | 1.22(0.96,1.48) | | -0.78(-0.92,-0.63) | |
| Islamic Republic of Pakistan | 0.99(0.83,1.19) | 1.02(0.85,1.22) | 2.18(1.83,2.59) | 1.11(0.95,1.29) | | 0.23(0.21,0.25) | |
| Jamaica | 0.03(0.02,0.03) | 1.42(1.21,1.66) | 0.05(0.05,0.06) | 1.93(1.69,2.22) | | 1.28(1.18,1.39) | |
| Japan | 12.44(9.84,15.71) | 8.46(6.9,10.26) | 22.26(17.89,26.81) | 10.61(8.83,12.73) | | 0.85(0.74,0.97) | |
| Kingdom of Bahrain | 0.01(0.01,0.01) | 2.36(2,2.7) | 0.02(0.02,0.02) | 1.5(1.29,1.74) | | -1.97(-2.12,-1.81) | |
| Kingdom of Belgium | 0.25(0.18,0.39) | 1.62(1.21,2.55) | 0.66(0.58,0.75) | 2.87(2.49,3.32) | | 0.93(-0.51,2.39) | |
| Kingdom of Bhutan | 0(0,0.01) | 0.87(0.73,1.05) | 0.01(0.01,0.01) | 0.99(0.85,1.14) | | 0.42(0.41,0.43) | |
| Kingdom of Cambodia | 0.05(0.04,0.06) | 0.75(0.62,0.88) | 0.13(0.11,0.15) | 0.88(0.75,1.01) | | 0.6(0.54,0.66) | |
| Kingdom of Denmark | 0.18(0.13,0.22) | 2.32(1.7,2.78) | 0.32(0.28,0.36) | 2.7(2.36,3.1) | | 0.66(0.26,1.06) | |
| Kingdom of Eswatini | 0.01(0.01,0.01) | 1.16(0.99,1.36) | 0.01(0.01,0.01) | 1.25(1.09,1.42) | | 0.2(0.14,0.26) | |
| Kingdom of Lesotho | 0.02(0.01,0.02) | 1.09(0.92,1.3) | 0.02(0.01,0.02) | 1.19(1.03,1.35) | | 0.29(0.26,0.32) | |
| Kingdom of Morocco | 0.17(0.14,0.21) | 0.85(0.71,1.02) | 0.29(0.25,0.34) | 0.78(0.67,0.9) | | -0.34(-0.37,-0.31) | |
| Kingdom of Norway | 0.9(0.72,1.13) | 15.95(12.87,20.21) | 1.33(1.05,1.67) | 15.61(12.5,19.07) | | 0.21(-0.04,0.46) | |
| Kingdom of Saudi Arabia | 0.09(0.07,0.11) | 0.82(0.68,0.99) | 0.27(0.21,0.34) | 0.76(0.66,0.89) | | -0.38(-0.46,-0.3) | |
| Kingdom of Spain | 0.92(0.69,1.44) | 1.66(1.27,2.61) | 2.8(2.42,3.66) | 2.92(2.47,3.89) | | 2.07(1.81,2.34) | |
| Kingdom of Sweden | 0.53(0.42,0.65) | 3.94(3.21,4.77) | 1.04(0.83,1.26) | 5.55(4.55,6.69) | | 0.44(-0.14,1.02) | |
| Kingdom of Thailand | 0.27(0.22,0.33) | 0.61(0.5,0.74) | 0.64(0.52,0.77) | 0.65(0.55,0.77) | | 0.18(0.16,0.21) | |
| Kingdom of the Netherlands | 0.68(0.54,0.84) | 3.51(2.8,4.36) | 1.4(1.1,1.7) | 4.17(3.35,5.03) | | 0.46(0.29,0.64) | |
| Kingdom of Tonga | 0(0,0) | 1.53(1.31,1.8) | 0(0,0) | 1.79(1.56,2.06) | | 0.5(0.44,0.56) | |
| Kyrgyz Republic | 0.02(0.02,0.03) | 0.7(0.58,0.86) | 0.04(0.03,0.05) | 0.65(0.53,0.78) | | -0.34(-0.41,-0.27) | |
| Lao People's Democratic Republic | 0.02(0.02,0.03) | 0.8(0.67,0.93) | 0.05(0.04,0.06) | 0.87(0.73,1.02) | | 0.31(0.27,0.36) | |
| Lebanese Republic | 0.02(0.02,0.03) | 0.89(0.74,1.05) | 0.05(0.04,0.05) | 0.81(0.7,0.94) | | -0.28(-0.31,-0.25) | |
| Malaysia | 0.14(0.11,0.17) | 1.24(1,1.52) | 0.46(0.39,0.56) | 1.57(1.32,1.87) | | 0.83(0.8,0.86) | |
| Mongolia | 0.01(0.01,0.01) | 0.62(0.51,0.78) | 0.02(0.01,0.02) | 0.62(0.52,0.75) | | 0.02(-0.02,0.07) | |
| Montenegro | 0.03(0.03,0.04) | 5.08(4.15,6.11) | 0.04(0.04,0.05) | 5.02(4.08,5.99) | | -0.01(-0.02,0.01) | |
| New Zealand | 0.29(0.23,0.36) | 7.34(5.96,8.94) | 0.53(0.45,0.62) | 6.19(5.27,7.18) | | -1.75(-2.38,-1.11) | |
| North Macedonia | 0.1(0.08,0.13) | 5.05(4.08,6.26) | 0.16(0.13,0.2) | 5.04(4.15,6.18) | | 0.01(-0.02,0.04) | |
| Northern Mariana Islands | 0(0,0) | 2.66(2.23,3.2) | 0(0,0) | 2.96(2.56,3.43) | | 0.28(0.09,0.47) | |
| Palestine | 0.01(0.01,0.01) | 0.74(0.6,0.89) | 0.03(0.02,0.03) | 0.7(0.59,0.82) | | -0.14(-0.2,-0.09) | |
| People's Democratic Republic of Algeria | 0.16(0.14,0.2) | 0.88(0.74,1.05) | 0.34(0.28,0.41) | 0.79(0.67,0.93) | | -0.35(-0.38,-0.31) | |
| People's Republic of Bangladesh | 0.81(0.67,0.98) | 0.91(0.75,1.07) | 1.33(1.13,1.58) | 0.89(0.76,1.03) | | -0.11(-0.13,-0.09) | |
| People's Republic of China | 32.56(26.33,40.36) | 3.2(2.59,3.97) | 80.88(65.94,99.15) | 3.88(3.21,4.69) | | 0.99(0.8,1.18) | |
| Plurinational State of Bolivia | 0.1(0.08,0.12) | 2.13(1.73,2.62) | 0.24(0.2,0.29) | 2.35(1.93,2.86) | | 0.34(0.29,0.39) | |
| Portuguese Republic | 0.18(0.13,0.23) | 1.24(0.97,1.66) | 0.63(0.52,0.78) | 2.56(2.12,3.19) | | 2.21(2.03,2.39) | |
| Principality of Andorra | 0(0,0) | 3.19(2.53,4.19) | 0.01(0,0.01) | 3.92(3.1,5) | | 0.63(0.51,0.75) | |
| Principality of Monaco | 0(0,0) | 1.45(1.18,1.79) | 0(0,0) | 1.52(1.23,1.87) | | 0.13(0.1,0.17) | |
| Puerto Rico | 0.06(0.05,0.08) | 1.82(1.5,2.18) | 0.11(0.09,0.13) | 2.07(1.75,2.42) | | 0.4(0.36,0.44) | |
| Republic of Albania | 0.14(0.12,0.17) | 5.7(4.62,6.91) | 0.25(0.2,0.31) | 6.33(5.15,7.57) | | 0.39(0.35,0.43) | |
| Republic of Angola | 0.04(0.03,0.05) | 0.53(0.45,0.63) | 0.12(0.1,0.15) | 0.61(0.53,0.7) | | 0.5(0.47,0.54) | |
| Republic of Armenia | 0.02(0.02,0.03) | 0.73(0.6,0.92) | 0.03(0.03,0.04) | 0.8(0.67,0.97) | | 0.48(0.44,0.53) | |
| Republic of Austria | 0.78(0.7,0.86) | 7.44(6.71,8.21) | 1.97(1.69,2.28) | 12.53(10.73,14.62) | | 2.2(1.24,3.17) | |
| Republic of Azerbaijan | 0.04(0.03,0.05) | 0.66(0.54,0.8) | 0.08(0.07,0.1) | 0.74(0.62,0.89) | | 0.49(0.45,0.53) | |
| Republic of Belarus | 1.52(1.17,1.87) | 11.56(9.12,14.14) | 1.96(1.58,2.43) | 13.07(10.63,15.89) | | 0.42(0.39,0.44) | |
| Republic of Benin | 0.04(0.03,0.05) | 1.48(1.03,1.8) | 0.08(0.07,0.1) | 1.29(0.99,1.6) | | -0.36(-0.56,-0.16) | |
| Republic of Botswana | 0.01(0.01,0.02) | 1.16(1,1.36) | 0.02(0.02,0.03) | 1.3(1.14,1.48) | | 0.42(0.22,0.63) | |
| Republic of Bulgaria | 0.6(0.48,0.76) | 5.14(4.15,6.33) | 0.58(0.45,0.71) | 5.17(4.19,6.3) | | 0.02(0.01,0.03) | |
| Republic of Burundi | 0.02(0.02,0.02) | 0.52(0.44,0.62) | 0.04(0.03,0.05) | 0.47(0.41,0.55) | | -0.3(-0.32,-0.29) | |
| Republic of Cabo Verde | 0(0,0) | 1.28(0.94,1.57) | 0.01(0,0.01) | 1.18(0.94,1.42) | | -0.25(-0.43,-0.06) | |
| Republic of Cameroon | 0.08(0.06,0.1) | 1.58(1.11,1.94) | 0.2(0.16,0.24) | 1.36(1.06,1.63) | | -0.48(-0.65,-0.31) | |
| Republic of Chad | 0.05(0.04,0.06) | 1.48(1.01,1.81) | 0.1(0.08,0.12) | 1.11(0.84,1.32) | | -0.88(-1.08,-0.68) | |
| Republic of Chile | 0.44(0.36,0.54) | 4.08(3.32,5.03) | 1.79(1.5,2.07) | 7.31(6.17,8.42) | | 1.77(1.65,1.89) | |
| Republic of Colombia | 0.93(0.76,1.13) | 4.2(3.41,5.18) | 2.83(2.32,3.48) | 5.36(4.43,6.54) | | 1(0.91,1.1) | |
| Republic of Costa Rica | 0.09(0.08,0.11) | 4.41(3.64,5.41) | 0.28(0.23,0.34) | 5.17(4.28,6.24) | | 0.63(0.58,0.68) | |
| Republic of Côte d'Ivoire | 0.08(0.06,0.1) | 1.5(1.06,1.84) | 0.17(0.14,0.21) | 1.27(1,1.53) | | -0.57(-0.77,-0.37) | |
| Republic of Croatia | 0.4(0.31,0.48) | 6.5(5.24,7.77) | 0.83(0.69,1) | 10.9(9.31,12.99) | | 1.73(1.38,2.07) | |
| Republic of Cuba | 0.13(0.1,0.15) | 1.26(1.06,1.51) | 0.2(0.17,0.24) | 1.34(1.14,1.56) | | 0.27(0.23,0.32) | |
| Republic of Cyprus | 0.03(0.02,0.03) | 3.01(2.44,3.64) | 0.07(0.06,0.09) | 3.54(3.11,4.06) | | 0.77(0.19,1.35) | |
| Republic of Djibouti | 0(0,0) | 0.47(0.39,0.56) | 0(0,0) | 0.54(0.47,0.62) | | 0.52(0.5,0.53) | |
| Republic of Ecuador | 0.2(0.16,0.27) | 2.73(2.23,3.82) | 0.66(0.54,0.76) | 3.92(3.25,4.55) | | 1.42(1.07,1.77) | |
| Republic of El Salvador | 0.12(0.1,0.14) | 3.27(2.68,3.94) | 0.19(0.16,0.22) | 3.14(2.55,3.73) | | -0.17(-0.18,-0.16) | |
| Republic of Equatorial Guinea | 0(0,0) | 0.51(0.43,0.61) | 0.01(0,0.01) | 0.72(0.63,0.82) | | 1.35(1.28,1.42) | |
| Republic of Estonia | 0.24(0.19,0.3) | 11.8(9.38,14.77) | 0.3(0.24,0.36) | 14.67(11.87,18.08) | | 0.75(0.72,0.77) | |
| Republic of Fiji | 0.01(0.01,0.01) | 1.39(1.18,1.64) | 0.02(0.01,0.02) | 1.76(1.53,2.02) | | 0.93(0.86,1.01) | |
| Republic of Finland | 0.4(0.34,0.47) | 5.92(4.99,6.87) | 0.66(0.56,0.77) | 6.11(5.2,7.09) | | -0.52(-1.36,0.33) | |
| Republic of Ghana | 0.1(0.08,0.13) | 1.26(0.91,1.54) | 0.22(0.18,0.26) | 1.1(0.85,1.31) | | -0.38(-0.56,-0.19) | |
| Republic of Guatemala | 0.18(0.16,0.22) | 3.42(2.79,4.13) | 0.46(0.38,0.53) | 3.72(3.03,4.38) | | 0.31(0.3,0.32) | |
| Republic of Guinea | 0.05(0.04,0.07) | 1.44(0.99,1.75) | 0.09(0.07,0.1) | 1.22(0.93,1.46) | | -0.48(-0.69,-0.26) | |
| Republic of Guinea-Bissau | 0.01(0.01,0.01) | 1.58(1.12,1.9) | 0.01(0.01,0.01) | 1.33(1,1.6) | | -0.5(-0.7,-0.29) | |
| Republic of Guyana | 0.01(0.01,0.01) | 1.42(1.19,1.7) | 0.01(0.01,0.01) | 1.58(1.36,1.86) | | 0.38(0.35,0.4) | |
| Republic of Haiti | 0.09(0.08,0.11) | 1.72(1.44,2.05) | 0.19(0.16,0.23) | 1.85(1.58,2.17) | | 0.27(0.24,0.31) | |
| Republic of Honduras | 0.12(0.1,0.15) | 4.15(3.39,5.16) | 0.35(0.29,0.43) | 4.76(3.95,5.86) | | 0.51(0.48,0.53) | |
| Republic of Iceland | 0(0,0) | 1.26(0.95,1.76) | 0.01(0.01,0.01) | 1.7(1.41,2.02) | | 1.76(1.25,2.26) | |
| Republic of India | 11.98(9.96,14.44) | 1.5(1.26,1.77) | 24.27(20.11,29.21) | 1.82(1.55,2.15) | | 1.02(0.85,1.2) | |
| Republic of Indonesia | 0.92(0.75,1.11) | 0.68(0.56,0.82) | 2.33(1.93,2.81) | 0.89(0.75,1.05) | | 0.92(0.89,0.95) | |
| Republic of Iraq | 0.1(0.08,0.13) | 0.75(0.6,0.93) | 0.25(0.2,0.3) | 0.69(0.57,0.81) | | -0.2(-0.35,-0.06) | |
| Republic of Italy | 10.08(8.04,12.7) | 12.44(10.08,15.64) | 11.92(10.07,13.88) | 10.43(9,12.09) | | -0.93(-1.16,-0.71) | |
| Republic of Kazakhstan | 0.11(0.09,0.13) | 0.73(0.6,0.88) | 0.17(0.14,0.2) | 0.85(0.72,1) | | 0.5(0.48,0.53) | |
| Republic of Kenya | 0.07(0.05,0.09) | 0.5(0.41,0.6) | 0.17(0.14,0.2) | 0.59(0.5,0.69) | | 0.4(0.34,0.45) | |
| Republic of Kiribati | 0(0,0) | 1.42(1.2,1.7) | 0(0,0) | 1.54(1.34,1.81) | | 0.3(0.27,0.34) | |
| Republic of Korea | 1.93(1.59,2.37) | 5.46(4.52,6.69) | 6.16(4.96,7.42) | 7.21(6,8.55) | | 1.12(1.04,1.2) | |
| Republic of Latvia | 0.42(0.34,0.52) | 11.99(9.76,14.65) | 0.47(0.38,0.57) | 15.53(12.79,18.81) | | 1.33(0.65,2.01) | |
| Republic of Liberia | 0.02(0.02,0.02) | 1.39(0.99,1.69) | 0.03(0.02,0.04) | 1.16(0.87,1.4) | | -0.52(-0.71,-0.33) | |
| Republic of Lithuania | 0.57(0.46,0.7) | 12.73(10.34,15.54) | 0.69(0.57,0.84) | 15.22(12.66,18.34) | | 0.17(-0.43,0.77) | |
| Republic of Madagascar | 0.03(0.03,0.04) | 0.43(0.36,0.51) | 0.07(0.06,0.09) | 0.43(0.37,0.5) | | 0.06(0.04,0.08) | |
| Republic of Malawi | 0.03(0.02,0.04) | 0.48(0.41,0.58) | 0.05(0.04,0.07) | 0.5(0.43,0.58) | | 0.14(0.12,0.16) | |
| Republic of Maldives | 0(0,0) | 1.12(0.9,1.37) | 0.01(0,0.01) | 1.49(1.24,1.76) | | 0.96(0.8,1.12) | |
| Republic of Mali | 0.39(0.33,0.46) | 10.59(8.7,12.75) | 1.34(1.16,1.55) | 15.91(13.44,18.92) | | 1.29(0.9,1.68) | |
| Republic of Malta | 0.01(0,0.01) | 1.45(1.13,1.85) | 0.02(0.02,0.03) | 2.14(1.72,2.62) | | 1.67(1.15,2.2) | |
| Republic of Mauritius | 0.01(0,0.01) | 0.71(0.58,0.87) | 0.02(0.01,0.02) | 0.89(0.76,1.02) | | 0.85(0.76,0.94) | |
| Republic of Moldova | 0.43(0.34,0.54) | 9.13(7.26,11.37) | 0.46(0.37,0.58) | 8.51(6.79,10.53) | | -0.31(-0.34,-0.28) | |
| Republic of Mozambique | 0.04(0.03,0.05) | 0.46(0.38,0.55) | 0.09(0.07,0.11) | 0.51(0.44,0.58) | | 0.36(0.33,0.39) | |
| Republic of Namibia | 0.01(0.01,0.02) | 1.16(1.01,1.35) | 0.02(0.02,0.03) | 1.28(1.13,1.46) | | 0.31(0.29,0.33) | |
| Republic of Nauru | 0(0,0) | 1.89(1.6,2.23) | 0(0,0) | 2.07(1.82,2.36) | | 0.31(0.2,0.43) | |
| Republic of Nicaragua | 0.08(0.07,0.1) | 3.73(3.05,4.49) | 0.23(0.19,0.28) | 4.22(3.5,5.05) | | 0.44(0.41,0.47) | |
| Republic of Niue | 0(0,0) | 1.76(1.51,2.08) | 0(0,0) | 1.99(1.74,2.29) | | 0.39(0.32,0.46) | |
| Republic of Palau | 0(0,0) | 1.88(1.62,2.19) | 0(0,0) | 2.06(1.8,2.37) | | 0.25(0.2,0.29) | |
| Republic of Panama | 0.08(0.07,0.1) | 4.95(4.05,5.99) | 0.21(0.17,0.24) | 4.73(3.92,5.56) | | -0.32(-0.39,-0.24) | |
| Republic of Paraguay | 0.02(0.01,0.02) | 0.67(0.56,0.81) | 0.06(0.05,0.07) | 1(0.87,1.15) | | 1.6(1.5,1.7) | |
| Republic of Peru | 0.28(0.23,0.34) | 1.8(1.47,2.23) | 0.61(0.5,0.73) | 1.77(1.45,2.12) | | -0.17(-0.25,-0.09) | |
| Republic of Poland | 1.34(1.1,1.66) | 3.16(2.61,3.91) | 2.56(2.23,2.92) | 3.95(3.5,4.42) | | 0.3(-0.6,1.21) | |
| Republic of Rwanda | 0.02(0.02,0.03) | 0.52(0.44,0.61) | 0.04(0.03,0.05) | 0.54(0.47,0.61) | | 0.09(0.05,0.14) | |
| Republic of San Marino | 0(0,0) | 2.61(2.05,3.38) | 0(0,0) | 2.99(2.41,3.75) | | 0.43(0.38,0.48) | |
| Republic of Senegal | 0.06(0.05,0.07) | 1.47(1.04,1.8) | 0.11(0.09,0.13) | 1.27(0.99,1.55) | | -0.43(-0.62,-0.25) | |
| Republic of Serbia | 1.1(0.88,1.34) | 9.19(7.49,11.02) | 1.49(1.19,1.82) | 10.7(8.69,12.77) | | 1.1(0.61,1.58) | |
| Republic of Seychelles | 0(0,0) | 0.84(0.7,0.98) | 0(0,0) | 0.98(0.84,1.14) | | 0.51(0.49,0.53) | |
| Republic of Sierra Leone | 0.04(0.03,0.04) | 1.44(1.01,1.77) | 0.06(0.04,0.07) | 1.19(0.89,1.42) | | -0.56(-0.77,-0.36) | |
| Republic of Singapore | 0.19(0.15,0.23) | 7.77(6.32,9.51) | 0.62(0.5,0.78) | 8.11(6.68,9.87) | | 0.14(0.13,0.15) | |
| Republic of Slovenia | 0.21(0.17,0.25) | 8.99(7.33,10.58) | 0.26(0.21,0.32) | 7.53(6.14,9.05) | | -0.59(-1.28,0.11) | |
| Republic of South Africa | 0.42(0.36,0.5) | 1.41(1.19,1.66) | 0.72(0.62,0.84) | 1.47(1.27,1.7) | | 0.09(0.04,0.14) | |
| Republic of South Sudan | 0.02(0.01,0.02) | 0.48(0.41,0.57) | 0.03(0.02,0.03) | 0.46(0.4,0.53) | | -0.13(-0.16,-0.1) | |
| Republic of Sudan | 0.13(0.11,0.16) | 0.84(0.69,1.01) | 0.26(0.21,0.31) | 0.74(0.64,0.86) | | -0.4(-0.48,-0.32) | |
| Republic of Suriname | 0(0,0.01) | 1.49(1.24,1.78) | 0.01(0.01,0.01) | 1.79(1.52,2.12) | | 0.67(0.64,0.7) | |
| Republic of Tajikistan | 0.02(0.02,0.03) | 0.59(0.47,0.74) | 0.05(0.04,0.06) | 0.61(0.5,0.74) | | 0.21(0.15,0.27) | |
| Republic of the Congo | 0.01(0.01,0.01) | 0.57(0.49,0.67) | 0.02(0.02,0.03) | 0.62(0.54,0.71) | | 0.28(0.24,0.32) | |
| Republic of the Gambia | 0.01(0.01,0.01) | 1.51(1.06,1.86) | 0.02(0.01,0.02) | 1.32(1.01,1.58) | | -0.4(-0.57,-0.22) | |
| Republic of the Marshall Islands | 0(0,0) | 1.64(1.39,1.93) | 0(0,0) | 1.91(1.68,2.18) | | 0.51(0.44,0.58) | |
| Republic of the Niger | 0.05(0.04,0.07) | 1.46(1,1.8) | 0.14(0.11,0.17) | 1.13(0.83,1.35) | | -0.73(-0.89,-0.57) | |
| Republic of the Philippines | 0.33(0.26,0.39) | 0.79(0.65,0.95) | 1.04(0.87,1.25) | 1.09(0.92,1.28) | | 1.91(1.48,2.35) | |
| Republic of the Union of Myanmar | 0.22(0.18,0.26) | 0.75(0.62,0.88) | 0.46(0.38,0.54) | 0.86(0.74,1) | | 0.51(0.49,0.54) | |
| Republic of Trinidad and Tobago | 0.02(0.02,0.02) | 1.82(1.58,2.1) | 0.04(0.03,0.04) | 2.13(1.87,2.44) | | 0.64(0.6,0.68) | |
| Republic of Tunisia | 0.06(0.05,0.07) | 0.83(0.69,0.99) | 0.1(0.08,0.12) | 0.77(0.65,0.9) | | -0.26(-0.32,-0.21) | |
| Republic of Turkey | 1.31(1.1,1.56) | 2.75(2.34,3.25) | 2.89(2.47,3.38) | 3.17(2.74,3.67) | | 1.09(0.63,1.54) | |
| Republic of Uganda | 0.05(0.04,0.07) | 0.47(0.4,0.56) | 0.13(0.1,0.16) | 0.54(0.47,0.61) | | 0.5(0.47,0.52) | |
| Republic of Uzbekistan | 0.09(0.08,0.11) | 0.62(0.5,0.78) | 0.2(0.16,0.25) | 0.64(0.53,0.78) | | 0.13(0.03,0.22) | |
| Republic of Vanuatu | 0(0,0) | 1.62(1.37,1.93) | 0(0,0.01) | 1.79(1.57,2.06) | | 0.32(0.24,0.4) | |
| Republic of Yemen | 0.09(0.07,0.1) | 0.83(0.68,1.01) | 0.2(0.16,0.23) | 0.74(0.64,0.87) | | -0.36(-0.44,-0.27) | |
| Republic of Zambia | 0.02(0.02,0.03) | 0.52(0.44,0.62) | 0.06(0.05,0.07) | 0.57(0.5,0.65) | | 0.33(0.31,0.36) | |
| Republic of Zimbabwe | 0.12(0.09,0.14) | 1.43(1.2,1.67) | 0.18(0.15,0.22) | 1.5(1.26,1.75) | | 0.13(0.12,0.14) | |
| Romania | 2.69(2.16,3.41) | 9.71(7.9,12.08) | 3.04(2.4,3.83) | 10.22(8.33,12.45) | | 0.63(0.23,1.04) | |
| Russian Federation | 16.79(13.34,21.44) | 9.21(7.37,11.56) | 22.22(17.53,27.74) | 10.23(8.27,12.71) | | 0.35(0.32,0.38) | |
| Saint Kitts and Nevis | 0(0,0) | 1.12(0.93,1.34) | 0(0,0) | 1.2(1.03,1.41) | | 0.25(0.23,0.28) | |
| Saint Lucia | 0(0,0) | 1.74(1.45,2.07) | 0(0,0.01) | 2.41(2.07,2.86) | | 1.15(1.11,1.2) | |
| Saint Vincent and the Grenadines | 0(0,0) | 1.96(1.65,2.33) | 0(0,0) | 2.64(2.26,3.1) | | 1.07(0.95,1.19) | |
| Slovak Republic | 0.39(0.32,0.47) | 6.64(5.45,8.08) | 0.53(0.42,0.65) | 6.4(5.15,7.65) | | -0.76(-1.18,-0.34) | |
| Socialist Republic of Viet Nam | 0.38(0.31,0.46) | 0.78(0.64,0.96) | 1.07(0.88,1.26) | 1.02(0.86,1.19) | | 0.96(0.92,1) | |
| Solomon Islands | 0(0,0) | 1.6(1.35,1.91) | 0.01(0.01,0.01) | 1.73(1.49,2.01) | | 0.25(0.2,0.31) | |
| State of Eritrea | 0.01(0.01,0.01) | 0.44(0.36,0.53) | 0.02(0.01,0.02) | 0.48(0.42,0.56) | | 0.32(0.27,0.37) | |
| State of Israel | 0.19(0.14,0.24) | 3.85(2.97,4.78) | 0.57(0.47,0.67) | 4.67(3.89,5.53) | | 0.66(0.39,0.92) | |
| State of Kuwait | 0.01(0.01,0.01) | 0.92(0.77,1.09) | 0.04(0.03,0.05) | 0.81(0.69,0.94) | | -0.54(-0.61,-0.47) | |
| State of Libya | 0.03(0.02,0.03) | 0.88(0.73,1.04) | 0.06(0.05,0.07) | 0.81(0.69,0.94) | | -0.29(-0.36,-0.22) | |
| State of Qatar | 0(0,0) | 0.87(0.72,1.04) | 0.02(0.02,0.03) | 0.86(0.72,1.05) | | 0.02(-0.03,0.06) | |
| Sultanate of Oman | 0.01(0.01,0.02) | 0.88(0.72,1.04) | 0.03(0.03,0.04) | 0.87(0.73,1) | | 0(-0.09,0.09) | |
| Swiss Confederation | 0.41(0.35,0.47) | 4.2(3.6,4.83) | 0.75(0.64,0.86) | 4.61(3.88,5.29) | | 0.04(-0.48,0.56) | |
| Syrian Arab Republic | 0.07(0.06,0.09) | 0.82(0.68,0.98) | 0.12(0.1,0.15) | 0.83(0.71,0.96) | | 0(-0.08,0.07) | |
| Taiwan (Province of China) | 0.43(0.35,0.51) | 2.39(1.97,2.83) | 1.01(0.86,1.16) | 2.58(2.24,2.92) | | 0.27(0.26,0.28) | |
| Togolese Republic | 0.02(0.02,0.03) | 1.52(1.05,1.87) | 0.05(0.04,0.07) | 1.37(1.04,1.67) | | -0.27(-0.46,-0.08) | |
| Tokelau | 0(0,0) | 1.61(1.38,1.88) | 0(0,0) | 1.79(1.57,2.06) | | 0.33(0.28,0.38) | |
| Turkmenistan | 0.02(0.01,0.02) | 0.67(0.55,0.83) | 0.03(0.03,0.04) | 0.7(0.59,0.85) | | 0.17(0.16,0.19) | |
| Tuvalu | 0(0,0) | 1.64(1.39,1.92) | 0(0,0) | 1.9(1.67,2.18) | | 0.49(0.42,0.55) | |
| Ukraine | 5.57(4.43,7.22) | 8.17(6.59,10.3) | 5.8(4.52,7.39) | 8.65(6.86,10.76) | | 0.18(0.16,0.19) | |
| Union of the Comoros | 0(0,0) | 0.47(0.4,0.55) | 0(0,0) | 0.51(0.45,0.58) | | 0.25(0.24,0.25) | |
| United Arab Emirates | 0.01(0.01,0.02) | 1.14(0.96,1.35) | 0.11(0.08,0.14) | 1.01(0.87,1.18) | | -0.55(-0.67,-0.42) | |
| United Kingdom of Great Britain and Northern Ireland | 7.06(5.47,8.86) | 8.49(6.7,10.46) | 12.86(10.38,15.74) | 11.1(9.09,13.61) | | -0.21(-0.53,0.11) | |
| United Mexican States | 3.33(2.8,3.96) | 5.45(4.49,6.59) | 7.93(6.61,9.6) | 6.14(5.16,7.45) | | 0.3(0.13,0.46) | |
| United Republic of Tanzania | 0.08(0.06,0.1) | 0.49(0.41,0.58) | 0.19(0.16,0.24) | 0.55(0.49,0.63) | | 0.46(0.42,0.49) | |
| United States of America | 23.7(18.75,29.54) | 7.62(6.12,9.35) | 51.96(43.29,62.28) | 9.73(8.28,11.4) | | 0.85(0.54,1.16) | |
| United States Virgin Islands | 0(0,0) | 1.97(1.68,2.32) | 0(0,0) | 2.62(2.29,3.01) | | 1.06(0.96,1.16) | |

Incidence cases: number of cases / thousands; ASIR: age-standardized incidence rate per 100,000 persons;

Abbreviations: ASR, age-standardized rate; SDI, socio-demographic index; GBD, global burden of diseases, injuries, and risk factors study; EAPC, estimated annual percentage change; CI, confidence interval; MDS/MPN: myelodysplastic syndromes /myeloproliferative neoplasms.

**Table S3 The case number and ASR of deaths of MDS/MPN in 1990 and 2021 by 204 countries, with EAPC from 1990 to 2021**

|  | **1990** | | **2021** | | **1990-2021** |
| --- | --- | --- | --- | --- | --- |
| **location** | **Deaths cases (95% CI)** | **ASDR(95% CI)** | **Deaths cases (95% CI)** | **ASDR (95% CI)** | **EAPCs(95% CI)** |
| American Samoa | 0(0,0) | 0.15(0.05,0.25) | 0(0,0) | 0.19(0.09,0.33) | 1.75(0.85,2.66) |
| Antigua and Barbuda | 0(0,0) | 0.06(0.05,0.08) | 0(0,0) | 0.22(0.2,0.25) | 5.08(4.59,5.57) |
| Arab Republic of Egypt | 0.01(0,0.01) | 0.03(0.01,0.05) | 0.02(0.01,0.04) | 0.04(0.03,0.08) | 1.05(0.97,1.14) |
| Argentine Republic | 0.16(0.14,0.2) | 0.52(0.44,0.65) | 0.49(0.43,0.55) | 0.86(0.75,0.95) | 2.32(2.09,2.56) |
| Australia | 0.34(0.27,0.44) | 1.78(1.45,2.3) | 0.84(0.71,0.95) | 1.6(1.37,1.8) | -0.39(-0.49,-0.29) |
| Barbados | 0(0,0) | 0.36(0.31,0.41) | 0(0,0) | 0.55(0.43,0.67) | 2.03(1.81,2.25) |
| Belize | 0(0,0) | 0.09(0.06,0.12) | 0(0,0) | 0.34(0.29,0.4) | 5.26(4.53,5.99) |
| Bermuda | 0(0,0) | 0.63(0.5,0.81) | 0(0,0) | 1.44(1.2,1.77) | 3.51(3.07,3.95) |
| Bolivarian Republic of Venezuela | 0.05(0.04,0.06) | 0.46(0.39,0.54) | 0.33(0.26,0.42) | 1.19(0.92,1.5) | 3.82(3.52,4.12) |
| Bosnia and Herzegovina | 0.01(0,0.02) | 0.27(0.13,0.5) | 0.03(0.01,0.04) | 0.41(0.23,0.68) | 1.64(1.53,1.76) |
| Brunei Darussalam | 0(0,0) | 1.38(0.69,2.43) | 0(0,0.01) | 1.52(1,2.42) | 0.84(0.64,1.04) |
| Burkina Faso | 0(0,0) | 0.01(0,0.02) | 0(0,0) | 0.01(0.01,0.03) | 2.59(2.29,2.9) |
| Canada | 0.51(0.42,0.64) | 1.59(1.31,1.99) | 1.17(1.03,1.29) | 1.43(1.26,1.57) | -0.38(-0.49,-0.27) |
| Central African Republic | 0(0,0) | 0.01(0,0.02) | 0(0,0) | 0.01(0.01,0.02) | 0.19(0.12,0.27) |
| Commonwealth of Dominica | 0(0,0) | 0.5(0.3,0.95) | 0(0,0) | 0.76(0.54,1.29) | 1.5(1.37,1.62) |
| Commonwealth of the Bahamas | 0(0,0) | 0.49(0.39,0.64) | 0(0,0) | 0.9(0.74,1.1) | 2.52(2.28,2.77) |
| Cook Islands | 0(0,0) | 0.04(0.01,0.08) | 0(0,0) | 0.06(0.02,0.09) | 0.84(0.71,0.98) |
| Czech Republic | 0.05(0.04,0.07) | 0.36(0.29,0.47) | 0.3(0.25,0.35) | 1.28(1.06,1.5) | 4.83(4.13,5.53) |
| Democratic People's Republic of Korea | 0.02(0.01,0.05) | 0.12(0.06,0.31) | 0.04(0.02,0.11) | 0.13(0.06,0.35) | 0.59(0.43,0.74) |
| Democratic Republic of Sao Tome and Principe | 0(0,0) | 0.01(0.01,0.02) | 0(0,0) | 0.02(0.01,0.03) | 3.01(2.57,3.45) |
| Democratic Republic of the Congo | 0(0,0) | 0.01(0.01,0.02) | 0(0,0.01) | 0.01(0,0.02) | 0.43(0.03,0.84) |
| Democratic Republic of Timor-Leste | 0(0,0) | 0.1(0.04,0.3) | 0(0,0) | 0.15(0.08,0.43) | 1.57(1.47,1.66) |
| Democratic Socialist Republic of Sri Lanka | 0.01(0.01,0.03) | 0.16(0.08,0.36) | 0.05(0.03,0.1) | 0.2(0.12,0.41) | 1.1(0.96,1.24) |
| Dominican Republic | 0.01(0,0.02) | 0.18(0.1,0.48) | 0.03(0.02,0.06) | 0.33(0.2,0.61) | 2.67(2.31,3.03) |
| Eastern Republic of Uruguay | 0.03(0.03,0.03) | 0.73(0.64,0.84) | 0.09(0.07,0.1) | 1.41(1.21,1.61) | 2.57(2.4,2.75) |
| Federal Democratic Republic of Ethiopia | 0(0,0) | 0.01(0,0.02) | 0.01(0,0.01) | 0.01(0.01,0.02) | 1.21(0.88,1.53) |
| Federal Democratic Republic of Nepal | 0(0,0.01) | 0.06(0.01,0.15) | 0.02(0.01,0.04) | 0.11(0.04,0.22) | 2.01(1.89,2.14) |
| Federal Republic of Germany | 1.62(1.32,2.06) | 1.19(0.97,1.49) | 3.48(2.95,3.9) | 1.47(1.27,1.63) | 1.04(0.87,1.2) |
| Federal Republic of Nigeria | 0(0,0.01) | 0.01(0.01,0.02) | 0.01(0.01,0.02) | 0.02(0.01,0.03) | 2.79(2.31,3.28) |
| Federal Republic of Somalia | 0(0,0) | 0.01(0,0.01) | 0(0,0) | 0.01(0,0.01) | -0.22(-0.29,-0.16) |
| Federated States of Micronesia | 0(0,0) | 0.04(0.01,0.07) | 0(0,0) | 0.04(0.01,0.08) | 0.36(0.31,0.41) |
| Federative Republic of Brazil | 0.27(0.24,0.31) | 0.32(0.28,0.37) | 1.63(1.45,1.77) | 0.68(0.6,0.74) | 3.16(2.94,3.37) |
| French Republic | 1.4(1.14,1.81) | 1.55(1.26,2) | 2.74(2.28,3.18) | 1.46(1.22,1.68) | -0.31(-0.44,-0.18) |
| Gabonese Republic | 0(0,0) | 0.02(0.01,0.04) | 0(0,0) | 0.04(0.02,0.05) | 1.23(1.09,1.37) |
| Georgia | 0(0,0) | 0(0,0) | 0.01(0.01,0.01) | 0.2(0.16,0.25) | 26.43(23.68,29.25) |
| Grand Duchy of Luxembourg | 0(0,0) | 0.64(0.53,0.81) | 0.01(0.01,0.01) | 0.91(0.77,1.05) | 1.73(1.48,1.97) |
| Greenland | 0(0,0) | 0.3(0.11,0.39) | 0(0,0) | 0.56(0.13,0.78) | 3.08(2.47,3.69) |
| Grenada | 0(0,0) | 0.47(0.28,0.65) | 0(0,0) | 1.21(1.03,1.38) | 3.49(3.23,3.74) |
| Guam | 0(0,0) | 0.15(0.04,0.21) | 0(0,0) | 0.07(0.03,0.15) | -1.81(-2.45,-1.16) |
| Hashemite Kingdom of Jordan | 0(0,0) | 0.05(0.02,0.12) | 0(0,0.01) | 0.07(0.04,0.13) | 2.39(1.88,2.92) |
| Hellenic Republic | 0.16(0.11,0.25) | 1.05(0.74,1.66) | 0.44(0.37,0.49) | 1.34(1.15,1.49) | 0.57(0.42,0.72) |
| Hungary | 0.1(0.08,0.13) | 0.71(0.57,0.9) | 0.29(0.24,0.34) | 1.36(1.13,1.6) | 2.48(2.07,2.9) |
| Independent State of Papua New Guinea | 0(0,0) | 0.03(0,0.06) | 0(0,0) | 0.03(0.01,0.06) | 0.36(0.28,0.45) |
| Independent State of Samoa | 0(0,0) | 0.04(0.01,0.08) | 0(0,0) | 0.05(0.02,0.08) | 0.58(0.54,0.63) |
| Ireland | 0.05(0.04,0.08) | 1.28(0.89,1.95) | 0.1(0.08,0.12) | 1.17(0.93,1.39) | -0.04(-0.19,0.11) |
| Islamic Republic of Afghanistan | 0(0,0.01) | 0.03(0.01,0.12) | 0(0,0.01) | 0.03(0.01,0.12) | 0.91(0.63,1.19) |
| Islamic Republic of Iran | 0.01(0,0.02) | 0.04(0.02,0.08) | 0.03(0.02,0.06) | 0.05(0.03,0.09) | 0.56(0.43,0.69) |
| Islamic Republic of Mauritania | 0(0,0) | 0.02(0.01,0.03) | 0(0,0) | 0.02(0.01,0.03) | 1.28(0.94,1.62) |
| Islamic Republic of Pakistan | 0.05(0.02,0.11) | 0.1(0.03,0.23) | 0.16(0.09,0.3) | 0.15(0.08,0.29) | 1.25(1.16,1.34) |
| Jamaica | 0.01(0.01,0.01) | 0.39(0.31,0.46) | 0.02(0.02,0.03) | 0.81(0.6,1.02) | 2.95(2.63,3.27) |
| Japan | 1.12(0.99,1.29) | 0.69(0.6,0.79) | 6.12(5.02,6.84) | 1.27(1.09,1.4) | 2.15(2.04,2.26) |
| Kingdom of Bahrain | 0(0,0) | 0.58(0.34,1.21) | 0(0,0.01) | 0.79(0.38,1.14) | 1.11(0.87,1.35) |
| Kingdom of Belgium | 0.18(0.15,0.23) | 1.12(0.92,1.4) | 0.39(0.32,0.44) | 1.35(1.14,1.53) | 0.82(0.7,0.94) |
| Kingdom of Bhutan | 0(0,0) | 0.07(0.02,0.16) | 0(0,0) | 0.15(0.06,0.27) | 2.52(2.46,2.58) |
| Kingdom of Cambodia | 0(0,0.01) | 0.12(0.05,0.34) | 0.02(0.01,0.05) | 0.22(0.12,0.49) | 2.34(2.19,2.5) |
| Kingdom of Denmark | 0.08(0.07,0.08) | 0.85(0.76,0.94) | 0.23(0.19,0.26) | 1.68(1.42,1.91) | 2.16(1.69,2.64) |
| Kingdom of Eswatini | 0(0,0) | 0.3(0.15,0.74) | 0(0,0) | 0.38(0.21,0.88) | 1.03(0.86,1.2) |
| Kingdom of Lesotho | 0(0,0) | 0.19(0.08,0.52) | 0(0,0.01) | 0.3(0.17,0.68) | 1.9(1.75,2.05) |
| Kingdom of Morocco | 0(0,0.02) | 0.03(0.01,0.13) | 0.01(0.01,0.05) | 0.04(0.02,0.16) | 1.39(1.23,1.54) |
| Kingdom of Norway | 0.07(0.06,0.09) | 0.97(0.84,1.14) | 0.12(0.1,0.13) | 1.01(0.88,1.11) | -0.03(-0.17,0.11) |
| Kingdom of Saudi Arabia | 0(0,0.01) | 0.05(0.02,0.11) | 0.01(0.01,0.02) | 0.08(0.04,0.14) | 1.49(1.05,1.94) |
| Kingdom of Spain | 0.68(0.55,0.89) | 1.24(1.01,1.62) | 1.97(1.63,2.26) | 1.54(1.3,1.76) | 0.83(0.71,0.95) |
| Kingdom of Sweden | 0.07(0.06,0.07) | 0.4(0.36,0.43) | 0.31(0.27,0.35) | 1.17(1,1.31) | 2.16(1.36,2.98) |
| Kingdom of Thailand | 0.02(0.01,0.06) | 0.07(0.04,0.2) | 0.18(0.1,0.31) | 0.17(0.1,0.29) | 2.75(2.42,3.08) |
| Kingdom of the Netherlands | 0.26(0.21,0.3) | 1.23(1.03,1.43) | 0.59(0.5,0.67) | 1.48(1.26,1.66) | 0.53(0.35,0.72) |
| Kingdom of Tonga | 0(0,0) | 0.03(0.01,0.06) | 0(0,0) | 0.04(0.01,0.07) | 0.58(0.52,0.63) |
| Kyrgyz Republic | 0(0,0) | 0(0,0) | 0.01(0.01,0.01) | 0.12(0.1,0.15) | 21.5(19.1,23.94) |
| Lao People's Democratic Republic | 0(0,0.01) | 0.11(0.04,0.32) | 0.01(0,0.02) | 0.2(0.11,0.48) | 2.23(2.16,2.3) |
| Lebanese Republic | 0.01(0,0.01) | 0.35(0.17,0.77) | 0.03(0.02,0.07) | 0.49(0.27,1.04) | 1.65(1.42,1.89) |
| Malaysia | 0.05(0.02,0.09) | 0.55(0.27,1.04) | 0.24(0.16,0.41) | 0.97(0.64,1.61) | 2.23(1.94,2.53) |
| Mongolia | 0(0,0) | 0.03(0.02,0.09) | 0(0,0) | 0.09(0.05,0.14) | 3.97(3.4,4.54) |
| Montenegro | 0(0,0) | 0(0,0) | 0(0,0) | 0(0,0) | 1.55(1.18,1.93) |
| New Zealand | 0.07(0.06,0.1) | 1.88(1.52,2.48) | 0.15(0.13,0.18) | 1.64(1.37,1.88) | -0.52(-0.63,-0.41) |
| North Macedonia | 0(0,0) | 0.07(0.03,0.12) | 0(0,0) | 0.09(0.05,0.14) | 0.87(0.72,1.01) |
| Northern Mariana Islands | 0(0,0) | 0.46(0.08,0.83) | 0(0,0) | 0.19(0.09,0.37) | -3.78(-4.15,-3.42) |
| Palestine | 0(0,0) | 0.09(0.04,0.15) | 0(0,0) | 0.12(0.06,0.17) | 0.8(0.58,1.02) |
| People's Democratic Republic of Algeria | 0(0,0.02) | 0.04(0.02,0.15) | 0.02(0.01,0.06) | 0.05(0.02,0.19) | 1.26(1.09,1.42) |
| People's Republic of Bangladesh | 0.04(0.01,0.09) | 0.08(0.02,0.2) | 0.16(0.07,0.34) | 0.13(0.05,0.28) | 1.45(1.36,1.54) |
| People's Republic of China | 1.25(0.62,2.63) | 0.16(0.08,0.34) | 4.74(2.8,8.78) | 0.25(0.15,0.45) | 1.68(1.54,1.82) |
| Plurinational State of Bolivia | 0.01(0,0.01) | 0.2(0.11,0.39) | 0.02(0.01,0.04) | 0.27(0.17,0.5) | 1.12(1.05,1.19) |
| Portuguese Republic | 0.02(0.01,0.02) | 0.13(0.11,0.15) | 0.49(0.41,0.54) | 1.59(1.37,1.76) | 4.34(2.27,6.46) |
| Principality of Andorra | 0(0,0) | 0.9(0.43,1.84) | 0(0,0) | 1.02(0.49,1.64) | 0.76(0.52,1) |
| Principality of Monaco | 0(0,0) | 0.05(0.03,0.08) | 0(0,0) | 0.07(0.04,0.11) | 1.02(0.99,1.05) |
| Puerto Rico | 0.02(0.02,0.03) | 0.69(0.58,0.86) | 0.07(0.06,0.08) | 0.88(0.71,1.04) | 1.08(0.87,1.29) |
| Republic of Albania | 0(0,0.01) | 0.19(0.07,0.32) | 0.01(0,0.02) | 0.28(0.11,0.48) | 1.85(1.66,2.05) |
| Republic of Angola | 0(0,0) | 0.01(0,0.02) | 0(0,0) | 0.02(0.01,0.04) | 2.19(2.05,2.33) |
| Republic of Armenia | 0(0,0) | 0.01(0.01,0.01) | 0.01(0.01,0.01) | 0.18(0.14,0.23) | 12.49(10.29,14.73) |
| Republic of Austria | 0.17(0.13,0.24) | 1.37(1.05,1.86) | 0.37(0.32,0.42) | 1.71(1.46,1.91) | 1.06(0.96,1.17) |
| Republic of Azerbaijan | 0(0,0) | 0.02(0.01,0.04) | 0(0,0) | 0.02(0.01,0.04) | 1.39(1.05,1.74) |
| Republic of Belarus | 0.06(0.03,0.08) | 0.44(0.28,0.61) | 0.12(0.1,0.15) | 0.77(0.61,0.94) | 1.74(1.5,1.98) |
| Republic of Benin | 0(0,0) | 0.01(0,0.01) | 0(0,0) | 0.02(0.01,0.03) | 2.84(2.57,3.11) |
| Republic of Botswana | 0(0,0) | 0.32(0.16,0.82) | 0(0,0.01) | 0.38(0.21,0.95) | 0.66(0.48,0.83) |
| Republic of Bulgaria | 0.01(0.01,0.01) | 0.11(0.08,0.14) | 0.02(0.02,0.03) | 0.15(0.12,0.19) | 1.24(1.1,1.39) |
| Republic of Burundi | 0(0,0) | 0.01(0,0.02) | 0(0,0) | 0.01(0,0.02) | -0.25(-0.41,-0.1) |
| Republic of Cabo Verde | 0(0,0) | 0.07(0.03,0.13) | 0(0,0) | 0.15(0.06,0.22) | 2.36(2.2,2.51) |
| Republic of Cameroon | 0(0,0) | 0.01(0.01,0.02) | 0(0,0) | 0.02(0.01,0.03) | 1.86(1.6,2.12) |
| Republic of Chad | 0(0,0) | 0(0,0.01) | 0(0,0) | 0.01(0,0.02) | 2.89(2.57,3.21) |
| Republic of Chile | 0.07(0.05,0.08) | 0.7(0.58,0.89) | 0.28(0.24,0.31) | 1.06(0.93,1.18) | 1.93(1.74,2.12) |
| Republic of Colombia | 0.08(0.07,0.1) | 0.4(0.34,0.5) | 0.53(0.43,0.64) | 0.98(0.8,1.19) | 3.44(3.18,3.7) |
| Republic of Costa Rica | 0.01(0.01,0.01) | 0.55(0.44,0.65) | 0.07(0.06,0.08) | 1.24(1.06,1.43) | 3.01(2.86,3.17) |
| Republic of Côte d'Ivoire | 0(0,0) | 0.01(0,0.02) | 0(0,0) | 0.02(0.01,0.03) | 2.45(2.29,2.62) |
| Republic of Croatia | 0.03(0.02,0.03) | 0.48(0.41,0.58) | 0.17(0.14,0.21) | 1.7(1.37,2.05) | 5.2(4.47,5.95) |
| Republic of Cuba | 0.02(0.02,0.02) | 0.2(0.17,0.23) | 0.07(0.06,0.08) | 0.36(0.31,0.42) | 2.44(2.09,2.79) |
| Republic of Cyprus | 0.01(0.01,0.03) | 2.78(1.17,6.07) | 0.03(0.02,0.06) | 1.51(0.83,2.84) | -1.92(-2.28,-1.57) |
| Republic of Djibouti | 0(0,0) | 0.01(0.01,0.02) | 0(0,0) | 0.02(0.01,0.04) | 1.08(1.05,1.11) |
| Republic of Ecuador | 0.01(0.01,0.01) | 0.15(0.13,0.18) | 0.09(0.07,0.11) | 0.57(0.46,0.71) | 4.55(3.5,5.6) |
| Republic of El Salvador | 0(0,0.01) | 0.07(0.04,0.16) | 0.01(0,0.01) | 0.11(0.06,0.2) | 1.69(1.56,1.82) |
| Republic of Equatorial Guinea | 0(0,0) | 0.01(0.01,0.02) | 0(0,0) | 0.04(0.02,0.06) | 3.61(3.36,3.85) |
| Republic of Estonia | 0.01(0.01,0.01) | 0.36(0.29,0.44) | 0.04(0.03,0.05) | 1.35(1.12,1.6) | 4.77(4.5,5.03) |
| Republic of Fiji | 0(0,0) | 0.05(0.02,0.14) | 0(0,0) | 0.1(0.05,0.18) | 3.49(2.73,4.26) |
| Republic of Finland | 0.07(0.06,0.09) | 1(0.86,1.19) | 0.19(0.16,0.21) | 1.22(1.03,1.38) | 0.92(0.78,1.05) |
| Republic of Ghana | 0(0,0) | 0.01(0,0.02) | 0(0,0) | 0(0,0) | -14.56(-17.6,-11.41) |
| Republic of Guatemala | 0.01(0,0.01) | 0.16(0.1,0.22) | 0.03(0.03,0.04) | 0.27(0.23,0.32) | 2.05(1.86,2.24) |
| Republic of Guinea | 0(0,0) | 0.01(0,0.01) | 0(0,0) | 0.01(0.01,0.02) | 2.99(2.82,3.16) |
| Republic of Guinea-Bissau | 0(0,0) | 0.01(0,0.02) | 0(0,0) | 0.02(0.01,0.03) | 2.85(2.67,3.04) |
| Republic of Guyana | 0(0,0) | 0.16(0.12,0.22) | 0(0,0) | 0.34(0.24,0.46) | 2.98(2.81,3.15) |
| Republic of Haiti | 0.01(0,0.02) | 0.24(0.1,0.51) | 0.02(0.01,0.04) | 0.3(0.14,0.58) | 0.95(0.85,1.05) |
| Republic of Honduras | 0.01(0,0.02) | 0.38(0.15,0.75) | 0.04(0.02,0.07) | 0.65(0.34,1.13) | 1.96(1.85,2.06) |
| Republic of Iceland | 0(0,0) | 0.48(0.41,0.55) | 0.01(0,0.01) | 0.89(0.74,1) | 2.75(2.35,3.16) |
| Republic of India | 0.29(0.11,0.58) | 0.07(0.03,0.16) | 1.4(0.86,2.52) | 0.13(0.08,0.24) | 1.96(1.76,2.16) |
| Republic of Indonesia | 0.11(0.07,0.28) | 0.12(0.07,0.33) | 0.46(0.28,1.06) | 0.24(0.14,0.54) | 2.23(2.14,2.32) |
| Republic of Iraq | 0(0,0) | 0.02(0.01,0.05) | 0.01(0,0.01) | 0.03(0.02,0.07) | 1.18(1.03,1.34) |
| Republic of Italy | 0.42(0.38,0.44) | 0.46(0.42,0.48) | 3.44(2.88,3.87) | 1.82(1.56,2.03) | 4.51(3.84,5.19) |
| Republic of Kazakhstan | 0.02(0.01,0.02) | 0.13(0.08,0.18) | 0.04(0.03,0.05) | 0.21(0.16,0.27) | 1.12(0.84,1.41) |
| Republic of Kenya | 0(0,0) | 0.01(0.01,0.02) | 0(0,0.01) | 0.02(0.01,0.03) | 2.05(1.93,2.17) |
| Republic of Kiribati | 0(0,0) | 0.02(0.01,0.04) | 0(0,0) | 0.02(0.01,0.04) | -0.38(-0.74,-0.01) |
| Republic of Korea | 0.13(0.08,0.32) | 0.54(0.32,1.29) | 0.87(0.44,1.5) | 0.94(0.48,1.63) | 2.42(2.24,2.61) |
| Republic of Latvia | 0.01(0.01,0.01) | 0.19(0.16,0.22) | 0.07(0.06,0.08) | 1.69(1.44,1.96) | 8.14(7.21,9.08) |
| Republic of Liberia | 0(0,0) | 0.01(0,0.01) | 0(0,0) | 0.01(0,0.02) | 2.24(1.73,2.76) |
| Republic of Lithuania | 0.02(0.02,0.03) | 0.55(0.46,0.63) | 0.09(0.07,0.1) | 1.43(1.19,1.65) | 3.57(3.19,3.95) |
| Republic of Madagascar | 0(0,0) | 0.01(0,0.02) | 0(0,0) | 0.01(0.01,0.02) | 0.58(0.41,0.75) |
| Republic of Malawi | 0(0,0) | 0.01(0,0.02) | 0(0,0) | 0.02(0.01,0.03) | 1.55(1.48,1.62) |
| Republic of Maldives | 0(0,0) | 0.39(0.15,0.9) | 0(0,0) | 0.58(0.34,0.95) | 1.31(1.13,1.49) |
| Republic of Mali | 0(0,0) | 0.01(0,0.01) | 0(0,0) | 0.01(0.01,0.02) | 2.67(2.29,3.07) |
| Republic of Malta | 0(0,0) | 0.78(0.66,0.95) | 0.02(0.01,0.02) | 1.42(1.18,1.65) | 2.21(1.72,2.7) |
| Republic of Mauritius | 0(0,0) | 0.18(0.12,0.23) | 0.01(0.01,0.01) | 0.58(0.52,0.63) | 3.48(2.56,4.42) |
| Republic of Moldova | 0(0,0) | 0.01(0.01,0.01) | 0.01(0,0.01) | 0.1(0.08,0.11) | 8.62(7.67,9.57) |
| Republic of Mozambique | 0(0,0) | 0.01(0,0.02) | 0(0,0) | 0.02(0.01,0.03) | 2.16(1.97,2.36) |
| Republic of Namibia | 0(0,0) | 0.32(0.17,0.84) | 0(0,0.01) | 0.43(0.24,1) | 1.02(0.98,1.05) |
| Republic of Nauru | 0(0,0) | 0.06(0.01,0.1) | 0(0,0) | 0.06(0.02,0.09) | -0.33(-0.62,-0.03) |
| Republic of Nicaragua | 0.01(0,0.01) | 0.25(0.13,0.53) | 0.02(0.01,0.03) | 0.36(0.21,0.59) | 1.84(1.61,2.08) |
| Republic of Niue | 0(0,0) | 0.05(0.02,0.07) | 0(0,0) | 0.06(0.02,0.09) | 0.68(0.57,0.78) |
| Republic of Palau | 0(0,0) | 0(0,0.01) | 0(0,0) | 0(0,0.01) | 1.06(0.84,1.28) |
| Republic of Panama | 0.01(0.01,0.01) | 0.58(0.49,0.68) | 0.04(0.03,0.06) | 1.02(0.76,1.28) | 2.22(2.06,2.39) |
| Republic of Paraguay | 0.01(0,0.01) | 0.23(0.13,0.47) | 0.02(0.01,0.04) | 0.44(0.25,0.73) | 2.75(2.59,2.92) |
| Republic of Peru | 0.02(0.01,0.03) | 0.14(0.07,0.26) | 0.06(0.03,0.1) | 0.17(0.09,0.31) | 0.05(-0.58,0.67) |
| Republic of Poland | 0.31(0.25,0.39) | 0.72(0.57,0.91) | 1.14(1,1.27) | 1.49(1.31,1.66) | 2.52(2.43,2.61) |
| Republic of Rwanda | 0(0,0) | 0.02(0.01,0.03) | 0(0,0) | 0.02(0.01,0.04) | 0.85(0.55,1.15) |
| Republic of San Marino | 0(0,0) | 0.84(0.49,1.58) | 0(0,0) | 0.55(0.29,1.1) | -0.27(-0.76,0.22) |
| Republic of Senegal | 0(0,0) | 0.01(0,0.02) | 0(0,0) | 0.02(0.01,0.03) | 2.98(2.64,3.31) |
| Republic of Serbia | 0.05(0.03,0.11) | 0.59(0.33,1.2) | 0.12(0.07,0.22) | 0.69(0.4,1.27) | 0.95(0.72,1.18) |
| Republic of Seychelles | 0(0,0) | 0.19(0.11,0.47) | 0(0,0) | 0.24(0.13,0.52) | 1.07(0.91,1.22) |
| Republic of Sierra Leone | 0(0,0) | 0.01(0,0.01) | 0(0,0) | 0.01(0.01,0.02) | 2.81(2.38,3.24) |
| Republic of Singapore | 0.01(0.01,0.02) | 0.48(0.29,0.82) | 0.04(0.04,0.05) | 0.53(0.46,0.61) | 0.23(0.05,0.41) |
| Republic of Slovenia | 0.02(0.01,0.02) | 0.7(0.58,0.9) | 0.05(0.04,0.06) | 1.01(0.87,1.17) | 1.39(1.07,1.71) |
| Republic of South Africa | 0.1(0.05,0.17) | 0.53(0.26,0.9) | 0.25(0.15,0.45) | 0.61(0.38,1.06) | 0.55(0.43,0.66) |
| Republic of South Sudan | 0(0,0) | 0.01(0,0.02) | 0(0,0) | 0.01(0,0.02) | 1.06(0.98,1.14) |
| Republic of Sudan | 0(0,0.01) | 0.02(0.01,0.11) | 0.01(0,0.03) | 0.03(0.01,0.14) | 1.4(1.3,1.5) |
| Republic of Suriname | 0(0,0) | 0.21(0.14,0.45) | 0(0,0) | 0.36(0.23,0.61) | 2.89(2.49,3.28) |
| Republic of Tajikistan | 0(0,0) | 0.01(0.01,0.05) | 0(0,0) | 0.03(0.01,0.06) | 3.12(2.74,3.5) |
| Republic of the Congo | 0(0,0) | 0.02(0.02,0.03) | 0(0,0) | 0.03(0.02,0.04) | 0.66(0.57,0.74) |
| Republic of the Gambia | 0(0,0) | 0.01(0,0.02) | 0(0,0) | 0.02(0.01,0.03) | 2.76(2.53,3) |
| Republic of the Marshall Islands | 0(0,0) | 0.03(0.01,0.06) | 0(0,0) | 0.04(0.01,0.07) | 0.54(0.45,0.62) |
| Republic of the Niger | 0(0,0) | 0(0,0.01) | 0(0,0) | 0.01(0,0.02) | 2.43(2.01,2.85) |
| Republic of the Philippines | 0.04(0.03,0.08) | 0.17(0.09,0.34) | 0.22(0.14,0.34) | 0.31(0.2,0.47) | 2.22(1.92,2.52) |
| Republic of the Union of Myanmar | 0.02(0.01,0.06) | 0.1(0.05,0.29) | 0.08(0.05,0.18) | 0.19(0.12,0.42) | 2.25(2.15,2.34) |
| Republic of Trinidad and Tobago | 0.01(0.01,0.01) | 0.8(0.66,0.99) | 0.02(0.02,0.03) | 1.14(0.85,1.45) | 1.46(1.32,1.61) |
| Republic of Tunisia | 0(0,0.01) | 0.04(0.01,0.15) | 0.01(0,0.02) | 0.04(0.02,0.17) | 0.76(0.7,0.83) |
| Republic of Turkey | 0.19(0.09,0.42) | 0.61(0.28,1.35) | 0.7(0.41,1.38) | 0.81(0.48,1.59) | 0.9(0.72,1.09) |
| Republic of Uganda | 0(0,0) | 0.01(0,0.01) | 0(0,0) | 0.02(0.01,0.03) | 2.65(2.55,2.76) |
| Republic of Uzbekistan | 0(0,0) | 0.01(0.01,0.02) | 0.01(0.01,0.01) | 0.04(0.03,0.05) | 3.01(2.66,3.36) |
| Republic of Vanuatu | 0(0,0) | 0.03(0,0.07) | 0(0,0) | 0.03(0.01,0.06) | -0.02(-0.07,0.02) |
| Republic of Yemen | 0(0,0.01) | 0.02(0.01,0.11) | 0(0,0.02) | 0.03(0.01,0.13) | 1.26(1.05,1.47) |
| Republic of Zambia | 0(0,0) | 0.01(0.01,0.02) | 0(0,0) | 0.02(0.01,0.04) | 1.86(1.61,2.11) |
| Republic of Zimbabwe | 0.02(0.01,0.04) | 0.61(0.29,1.1) | 0.04(0.02,0.07) | 0.67(0.42,1.18) | 0.39(0.14,0.64) |
| Romania | 0.03(0.03,0.04) | 0.12(0.1,0.15) | 0.1(0.09,0.12) | 0.26(0.22,0.31) | 2.83(2.63,3.02) |
| Russian Federation | 0.21(0.18,0.25) | 0.12(0.1,0.14) | 0.83(0.74,0.92) | 0.35(0.31,0.38) | 3.8(3.28,4.31) |
| Saint Kitts and Nevis | 0(0,0) | 0.07(0.05,0.09) | 0(0,0) | 0.19(0.15,0.22) | 4.33(3.92,4.75) |
| Saint Lucia | 0(0,0) | 0.31(0.25,0.4) | 0(0,0) | 0.89(0.72,1.08) | 3.93(3.55,4.31) |
| Saint Vincent and the Grenadines | 0(0,0) | 0.54(0.42,0.68) | 0(0,0) | 1.07(0.9,1.25) | 2.63(2.35,2.91) |
| Slovak Republic | 0.01(0,0.01) | 0.1(0.06,0.23) | 0.02(0.01,0.03) | 0.19(0.12,0.31) | 2.72(2.35,3.1) |
| Socialist Republic of Viet Nam | 0.05(0.02,0.14) | 0.12(0.06,0.35) | 0.21(0.13,0.49) | 0.24(0.15,0.55) | 2.37(2.27,2.47) |
| Solomon Islands | 0(0,0) | 0.03(0,0.07) | 0(0,0) | 0.03(0.01,0.07) | 0.04(-0.07,0.15) |
| State of Eritrea | 0(0,0) | 0.01(0,0.02) | 0(0,0) | 0.02(0.01,0.04) | 1.72(1.48,1.97) |
| State of Israel | 0.06(0.05,0.07) | 1.19(1.02,1.42) | 0.2(0.17,0.23) | 1.44(1.21,1.63) | 0.75(0.54,0.95) |
| State of Kuwait | 0(0,0) | 0.06(0.05,0.07) | 0(0,0.01) | 0.18(0.14,0.22) | 5.54(4.31,6.77) |
| State of Libya | 0(0,0) | 0.05(0.02,0.19) | 0(0,0.01) | 0.08(0.03,0.29) | 2.01(1.71,2.32) |
| State of Qatar | 0(0,0) | 0.36(0.1,0.75) | 0(0,0.01) | 0.41(0.18,0.84) | 1.34(0.89,1.79) |
| Sultanate of Oman | 0(0,0) | 0.05(0.02,0.15) | 0(0,0) | 0.09(0.02,0.14) | 2.79(2.42,3.17) |
| Swiss Confederation | 0.13(0.11,0.15) | 1.11(0.96,1.29) | 0.32(0.26,0.36) | 1.41(1.19,1.61) | 1.14(0.86,1.41) |
| Syrian Arab Republic | 0.01(0.01,0.03) | 0.25(0.1,0.55) | 0.03(0.01,0.06) | 0.26(0.13,0.54) | 0.14(0.01,0.27) |
| Taiwan (Province of China) | 0.05(0.03,0.08) | 0.33(0.19,0.52) | 0.17(0.15,0.19) | 0.42(0.36,0.47) | 0.6(0.37,0.84) |
| Togolese Republic | 0(0,0) | 0.01(0,0.02) | 0(0,0) | 0.02(0.01,0.04) | 2.42(2.12,2.74) |
| Tokelau | 0(0,0) | 0.04(0.01,0.08) | 0(0,0) | 0.08(0.02,0.13) | 0.85(0.43,1.27) |
| Turkmenistan | 0(0,0) | 0.02(0.02,0.03) | 0(0,0) | 0.05(0.03,0.07) | 2.75(2.46,3.04) |
| Tuvalu | 0(0,0) | 0.03(0.01,0.06) | 0(0,0) | 0.05(0.01,0.08) | 0.99(0.89,1.08) |
| Ukraine | 0.08(0.06,0.1) | 0.11(0.09,0.14) | 0.12(0.09,0.17) | 0.16(0.12,0.22) | 1.21(1.03,1.4) |
| Union of the Comoros | 0(0,0) | 0.01(0,0.02) | 0(0,0) | 0.02(0.01,0.03) | 1.11(1.03,1.19) |
| United Arab Emirates | 0(0,0.01) | 1(0.37,1.83) | 0.02(0.01,0.05) | 1.24(0.7,2.02) | 2.64(1.99,3.28) |
| United Kingdom of Great Britain and Northern Ireland | 1.27(1.06,1.59) | 1.32(1.1,1.65) | 2.26(1.97,2.43) | 1.46(1.29,1.57) | 0.23(0.05,0.41) |
| United Mexican States | 0.26(0.22,0.33) | 0.54(0.46,0.66) | 0.92(0.8,1.04) | 0.76(0.66,0.86) | 1.28(1.06,1.49) |
| United Republic of Tanzania | 0(0,0) | 0.01(0,0.02) | 0(0,0.01) | 0.02(0.01,0.03) | 1.63(1.51,1.75) |
| United States of America | 5.2(4.51,5.98) | 1.53(1.33,1.76) | 10.13(8.8,11.04) | 1.59(1.4,1.73) | 0.15(0.05,0.25) |
| United States Virgin Islands | 0(0,0) | 0.63(0.3,1.5) | 0(0,0) | 0.5(0.23,1.12) | -0.67(-0.99,-0.35) |

Deaths cases: number of cases / thousands; ASDR: age-standardized deaths rate per 100,000 persons;

Abbreviations: ASR, age-standardized rate; SDI, socio-demographic index; GBD, global burden of diseases, injuries, and risk factors study; EAPC, estimated annual percentage change; CI, confidence interval; MDS/MPN: myelodysplastic syndromes /myeloproliferative neoplasms.

**Table S4 The case number and ASR of DALYs of MDS/MPN in 1990 and 2021 by 204 countries, with EAPC from 1990 to 2021**

|  | **1990** | | **2021** | | **1990-2021** |
| --- | --- | --- | --- | --- | --- |
| **location** | **Dalys cases (95% CI)** | **ASR of DALYs (95% CI)** | **Dalys cases (95% CI)** | **ASR of DALYs(95% CI)** | **EAPCs(95% CI)** |
| American Samoa | 0(0,0) | 5.08(2.53,10.26) | 0(0,0.01) | 7.01(3.72,12.34) | 1.79(1.17,2.4) |
| Antigua and Barbuda | 0(0,0) | 2.49(1.98,3.08) | 0.01(0.01,0.01) | 5.98(5.4,6.77) | 3.67(3.34,4) |
| Arab Republic of Egypt | 0.47(0.33,0.67) | 1.31(0.88,1.86) | 1.11(0.8,1.58) | 1.5(1.07,2.24) | 0.49(0.45,0.53) |
| Argentine Republic | 4.12(3.51,5.02) | 12.71(10.84,15.49) | 10.55(9.25,11.69) | 19.28(16.94,21.33) | 2.01(1.77,2.24) |
| Australia | 6.23(5.12,7.92) | 31.93(26.28,40.61) | 13.03(11.48,14.36) | 27.03(24.14,29.67) | -0.63(-0.75,-0.51) |
| Barbados | 0.03(0.02,0.03) | 9.44(8.23,10.95) | 0.06(0.05,0.07) | 13.29(10.52,16.34) | 1.65(1.45,1.85) |
| Belize | 0.01(0,0.01) | 3.48(2.54,4.42) | 0.03(0.03,0.04) | 9.8(8.38,11.32) | 4.1(3.58,4.61) |
| Bermuda | 0.01(0.01,0.01) | 15.06(11.92,19.03) | 0.04(0.03,0.05) | 31.92(27.03,38.55) | 3.22(2.82,3.61) |
| Bolivarian Republic of Venezuela | 2.2(1.88,2.57) | 16.14(13.69,18.85) | 10.44(8.2,13.05) | 37.05(29.35,46.41) | 3.36(3.12,3.59) |
| Bosnia and Herzegovina | 0.4(0.27,0.63) | 10(6.45,15.41) | 0.74(0.5,1.1) | 12.62(8.62,18.76) | 1.04(0.96,1.11) |
| Brunei Darussalam | 0.05(0.03,0.09) | 37.3(22.91,63.12) | 0.13(0.1,0.22) | 38.61(27.24,60.52) | 0.5(0.37,0.64) |
| Burkina Faso | 0.03(0.02,0.04) | 0.46(0.28,0.68) | 0.06(0.04,0.09) | 0.51(0.32,0.73) | 0.46(0.4,0.53) |
| Canada | 10.32(8.57,12.77) | 32.05(26.68,39.65) | 19.89(17.77,21.95) | 27.34(24.48,30.34) | -0.55(-0.64,-0.45) |
| Central African Republic | 0.01(0,0.01) | 0.43(0.25,0.66) | 0.01(0.01,0.02) | 0.42(0.24,0.65) | -0.06(-0.12,0) |
| Commonwealth of Dominica | 0.01(0.01,0.02) | 12.81(8.09,23.11) | 0.01(0.01,0.03) | 19.96(14.44,33.5) | 1.65(1.53,1.77) |
| Commonwealth of the Bahamas | 0.03(0.02,0.04) | 14.29(11.66,18.35) | 0.09(0.08,0.12) | 23.97(19.69,29.55) | 2.17(1.94,2.41) |
| Cook Islands | 0(0,0) | 2.33(1.17,3.53) | 0(0,0) | 3.18(1.45,4.78) | 0.81(0.66,0.97) |
| Czech Republic | 1.47(1.18,1.85) | 10.82(8.72,13.65) | 6(5.19,6.95) | 27.91(24.21,32.29) | 3.65(3.08,4.22) |
| Democratic People's Republic of Korea | 0.97(0.61,2.11) | 5.28(3.35,11.25) | 1.7(0.97,3.62) | 5.59(3.17,12.26) | 0.41(0.3,0.53) |
| Democratic Republic of Sao Tome and Principe | 0(0,0) | 0.54(0.35,0.77) | 0(0,0) | 0.66(0.45,0.91) | 0.94(0.8,1.08) |
| Democratic Republic of the Congo | 0.1(0.07,0.15) | 0.44(0.28,0.64) | 0.22(0.13,0.33) | 0.44(0.23,0.69) | 0.02(-0.21,0.25) |
| Democratic Republic of Timor-Leste | 0.01(0.01,0.03) | 2.67(1.3,7.39) | 0.04(0.02,0.09) | 3.89(2.29,9.65) | 1.3(1.17,1.43) |
| Democratic Socialist Republic of Sri Lanka | 0.43(0.26,0.87) | 3.74(2.2,7.74) | 1.2(0.7,2.31) | 4.62(2.76,8.89) | 0.89(0.78,0.99) |
| Dominican Republic | 0.33(0.23,0.64) | 5.86(3.93,12.63) | 1.03(0.65,1.69) | 9.93(6.29,16.44) | 2.33(2.09,2.57) |
| Eastern Republic of Uruguay | 0.66(0.58,0.75) | 17.29(15.34,19.78) | 1.63(1.42,1.85) | 30.37(26.64,34.4) | 2.18(2.03,2.32) |
| Federal Democratic Republic of Ethiopia | 0.11(0.07,0.17) | 0.35(0.19,0.58) | 0.26(0.16,0.38) | 0.42(0.25,0.62) | 0.52(0.33,0.71) |
| Federal Democratic Republic of Nepal | 0.18(0.08,0.38) | 1.63(0.62,3.43) | 0.64(0.3,1.17) | 2.66(1.19,4.82) | 1.76(1.66,1.86) |
| Federal Republic of Germany | 28.66(23.53,35.88) | 22.18(18.31,27.64) | 53.06(46.37,58.62) | 25.69(23.01,28.06) | 0.81(0.67,0.94) |
| Federal Republic of Nigeria | 0.26(0.17,0.37) | 0.47(0.32,0.65) | 0.64(0.44,0.89) | 0.61(0.42,0.81) | 1.07(0.88,1.25) |
| Federal Republic of Somalia | 0.01(0.01,0.02) | 0.23(0.11,0.41) | 0.02(0.01,0.04) | 0.21(0.1,0.41) | -0.32(-0.35,-0.29) |
| Federated States of Micronesia | 0(0,0) | 2.11(1.02,3.5) | 0(0,0) | 2.42(1.19,3.88) | 0.51(0.46,0.56) |
| Federative Republic of Brazil | 8.61(7.68,10.1) | 8.09(7.28,9.41) | 38.36(35.05,41.07) | 15.86(14.53,16.98) | 2.81(2.6,3.01) |
| French Republic | 22.95(19.12,29.23) | 27.01(22.58,34.25) | 39.47(33.45,45.12) | 25.49(21.91,28.77) | -0.3(-0.42,-0.18) |
| Gabonese Republic | 0(0,0.01) | 0.7(0.42,0.99) | 0.01(0.01,0.01) | 0.92(0.6,1.22) | 0.8(0.69,0.91) |
| Georgia | 0.03(0.02,0.04) | 0.48(0.27,0.71) | 0.32(0.26,0.39) | 6.7(5.46,8.12) | 11.37(9.86,12.9) |
| Grand Duchy of Luxembourg | 0.07(0.06,0.09) | 13.39(11.16,16.62) | 0.19(0.16,0.21) | 17.02(14.72,19.24) | 1.15(0.86,1.43) |
| Greenland | 0(0,0) | 8.41(5.61,10.72) | 0.01(0,0.01) | 12.23(5.98,16.12) | 1.81(1.46,2.17) |
| Grenada | 0.01(0.01,0.01) | 13.1(8.52,17.86) | 0.03(0.03,0.04) | 29.45(25.34,33.72) | 3.07(2.89,3.24) |
| Guam | 0.01(0,0.01) | 5.51(2.15,9.59) | 0.01(0,0.01) | 4.95(2.08,8.14) | 0.21(-0.37,0.78) |
| Hashemite Kingdom of Jordan | 0.03(0.01,0.06) | 1.25(0.72,2.94) | 0.15(0.09,0.26) | 1.81(1.05,3.12) | 2.06(1.59,2.53) |
| Hellenic Republic | 3.25(2.36,4.7) | 21.45(15.69,31.07) | 9.09(7.64,10.73) | 35.14(29.14,42.53) | 2.22(1.94,2.5) |
| Hungary | 3.02(2.46,3.74) | 21.04(17.16,26.08) | 6.48(5.48,7.6) | 34.45(29.16,40.84) | 1.85(1.54,2.16) |
| Independent State of Papua New Guinea | 0.05(0.02,0.08) | 1.71(0.81,2.68) | 0.17(0.07,0.27) | 2(0.93,3.06) | 0.5(0.46,0.55) |
| Independent State of Samoa | 0(0,0) | 2.28(1.11,3.71) | 0.01(0,0.01) | 2.72(1.37,4.17) | 0.64(0.57,0.72) |
| Ireland | 0.98(0.68,1.46) | 23.53(16.5,34.98) | 1.73(1.4,2.03) | 21.23(17.12,24.86) | -0.16(-0.3,-0.03) |
| Islamic Republic of Afghanistan | 0.11(0.06,0.27) | 1.31(0.71,3.41) | 0.26(0.16,0.6) | 1.38(0.77,3.51) | 0.34(0.2,0.49) |
| Islamic Republic of Iran | 0.76(0.46,1.07) | 1.9(1.2,2.87) | 1.45(0.98,2.16) | 1.8(1.22,2.72) | -0.17(-0.45,0.11) |
| Islamic Republic of Mauritania | 0.01(0,0.01) | 0.6(0.39,0.87) | 0.02(0.01,0.02) | 0.65(0.43,0.9) | 0.28(0.16,0.4) |
| Islamic Republic of Pakistan | 1.91(1.04,3.45) | 2.76(1.4,5.34) | 5.71(3.96,9.63) | 3.9(2.57,6.95) | 1.03(0.96,1.1) |
| Jamaica | 0.22(0.18,0.26) | 10.7(8.66,12.69) | 0.59(0.45,0.75) | 20.04(15.22,25.34) | 2.52(2.22,2.82) |
| Japan | 35.94(30.02,42.84) | 23.1(19.28,27.55) | 108.78(94.7,122.36) | 32.43(28.22,36.75) | 1.09(0.95,1.23) |
| Kingdom of Bahrain | 0.03(0.02,0.06) | 13.08(7.92,26.87) | 0.15(0.07,0.25) | 16.23(8.02,24.99) | 0.8(0.59,1.01) |
| Kingdom of Belgium | 3.03(2.55,3.76) | 19.47(16.42,24.09) | 5.84(4.97,6.59) | 23.59(20.57,26.3) | 0.74(0.63,0.84) |
| Kingdom of Bhutan | 0.01(0,0.01) | 2.19(1.05,4.34) | 0.02(0.01,0.04) | 3.72(1.91,6.66) | 1.81(1.76,1.86) |
| Kingdom of Cambodia | 0.18(0.09,0.45) | 3.13(1.58,8.3) | 0.69(0.4,1.62) | 5.31(3.09,12.03) | 1.97(1.84,2.1) |
| Kingdom of Denmark | 1.35(1.22,1.5) | 16.03(14.59,17.7) | 3.52(3.04,3.96) | 27.88(24.27,31.21) | 1.75(1.25,2.25) |
| Kingdom of Eswatini | 0.03(0.01,0.06) | 7.18(3.98,17.07) | 0.06(0.03,0.14) | 9.13(5.14,21.38) | 0.92(0.76,1.09) |
| Kingdom of Lesotho | 0.05(0.02,0.11) | 4.78(2.37,11.96) | 0.09(0.05,0.2) | 7.2(4.15,16.83) | 1.71(1.57,1.85) |
| Kingdom of Morocco | 0.28(0.17,0.64) | 1.43(0.81,3.6) | 0.59(0.34,1.45) | 1.67(0.97,4.28) | 0.65(0.58,0.72) |
| Kingdom of Norway | 1.96(1.62,2.33) | 28.6(23.41,34.27) | 2.74(2.3,3.27) | 27.11(22.65,32.5) | -0.19(-0.27,-0.12) |
| Kingdom of Saudi Arabia | 0.15(0.09,0.24) | 1.77(1.05,2.96) | 0.59(0.37,0.84) | 2.26(1.4,3.48) | 0.96(0.66,1.26) |
| Kingdom of Spain | 11.8(9.71,15.33) | 21.85(17.98,28.45) | 27.68(23.69,31.21) | 25.68(22.49,28.78) | 0.58(0.47,0.68) |
| Kingdom of Sweden | 1.43(1.27,1.62) | 9.34(8.31,10.57) | 4.97(4.33,5.56) | 21.16(18.56,23.58) | 1.53(0.82,2.26) |
| Kingdom of Thailand | 0.8(0.5,1.77) | 2.02(1.24,4.66) | 4.12(2.39,7.02) | 4.11(2.39,6.82) | 2.26(1.99,2.54) |
| Kingdom of the Netherlands | 4.59(3.92,5.28) | 22.5(19.36,25.87) | 9.29(8.15,10.27) | 25.26(22.43,27.85) | 0.37(0.22,0.51) |
| Kingdom of Tonga | 0(0,0) | 1.94(0.93,3.01) | 0(0,0) | 2.35(1.14,3.59) | 0.65(0.62,0.68) |
| Kyrgyz Republic | 0.02(0.01,0.03) | 0.56(0.34,0.85) | 0.27(0.22,0.32) | 4.5(3.64,5.4) | 8.2(6.87,9.54) |
| Lao People's Democratic Republic | 0.07(0.04,0.19) | 2.95(1.45,7.81) | 0.26(0.15,0.56) | 4.97(2.91,11.18) | 1.87(1.81,1.93) |
| Lebanese Republic | 0.2(0.1,0.4) | 8.48(4.46,17.44) | 0.64(0.37,1.3) | 10.61(6.12,21.3) | 1.2(1.01,1.39) |
| Malaysia | 1.29(0.7,2.39) | 12.58(6.72,23.31) | 5.67(3.7,9.77) | 20.34(13.45,34.84) | 1.82(1.61,2.02) |
| Mongolia | 0.03(0.02,0.07) | 1.7(1,3.72) | 0.11(0.06,0.17) | 3.61(2.17,5.51) | 3.05(2.62,3.48) |
| Montenegro | 0.03(0.02,0.04) | 4.44(2.68,6.59) | 0.04(0.02,0.06) | 4.35(2.67,6.41) | -0.02(-0.04,0) |
| New Zealand | 1.36(1.09,1.81) | 34.7(27.77,45.93) | 2.45(2.13,2.79) | 27.78(24.19,31.5) | -0.97(-1.12,-0.83) |
| North Macedonia | 0.11(0.07,0.15) | 5.77(3.89,8.06) | 0.18(0.12,0.25) | 5.92(4.04,8.13) | 0.14(0.1,0.17) |
| Northern Mariana Islands | 0(0,0.01) | 13.59(3.34,23) | 0(0,0.01) | 7.78(3.82,12.33) | -2.19(-2.46,-1.93) |
| Palestine | 0.03(0.02,0.05) | 2.68(1.35,3.93) | 0.1(0.06,0.13) | 3.05(1.88,4.34) | 0.56(0.37,0.74) |
| People's Democratic Republic of Algeria | 0.3(0.18,0.65) | 1.64(0.95,3.99) | 0.7(0.42,1.78) | 1.81(1.06,4.82) | 0.46(0.39,0.53) |
| People's Republic of Bangladesh | 1.48(0.75,2.82) | 2.37(1.12,4.95) | 4.7(2.42,8.77) | 3.3(1.68,6.3) | 1.03(0.99,1.07) |
| People's Republic of China | 73.41(48.26,124.01) | 7.5(4.95,12.69) | 184.69(128.1,288.7) | 9.77(6.77,15.59) | 1.01(0.9,1.11) |
| Plurinational State of Bolivia | 0.32(0.22,0.55) | 6.79(4.48,11.72) | 0.82(0.56,1.42) | 8.27(5.67,14.38) | 0.68(0.62,0.74) |
| Portuguese Republic | 0.4(0.33,0.47) | 3.16(2.61,3.67) | 7.15(6.26,7.85) | 27.24(24.05,29.69) | 3.74(1.92,5.59) |
| Principality of Andorra | 0.01(0,0.02) | 17.45(9.13,33.07) | 0.03(0.01,0.04) | 18.4(9.67,28.06) | 0.52(0.32,0.72) |
| Principality of Monaco | 0(0,0) | 1.76(1.17,2.5) | 0(0,0) | 2.07(1.41,2.87) | 0.51(0.49,0.53) |
| Puerto Rico | 0.61(0.52,0.74) | 16.76(14.34,20.46) | 1.29(1.07,1.53) | 21.26(17.65,24.86) | 1.06(0.86,1.26) |
| Republic of Albania | 0.19(0.12,0.27) | 8.55(5.41,12.12) | 0.43(0.25,0.62) | 10.26(6.23,14.83) | 0.91(0.81,1) |
| Republic of Angola | 0.03(0.02,0.04) | 0.45(0.25,0.64) | 0.11(0.08,0.16) | 0.68(0.44,1) | 1.4(1.3,1.49) |
| Republic of Armenia | 0.03(0.02,0.04) | 0.9(0.63,1.25) | 0.23(0.18,0.29) | 6.15(4.86,7.7) | 8.19(6.69,9.71) |
| Republic of Austria | 3.39(2.68,4.47) | 28.64(22.69,37.63) | 6.71(5.85,7.63) | 35.16(31,39.97) | 1.11(0.84,1.39) |
| Republic of Azerbaijan | 0.07(0.04,0.14) | 1.14(0.65,2.23) | 0.13(0.07,0.24) | 1.28(0.7,2.31) | 0.94(0.73,1.15) |
| Republic of Belarus | 2.46(1.74,3.22) | 19.3(13.74,25.43) | 4.23(3.39,5.23) | 27.36(21.96,33.51) | 1.12(0.95,1.29) |
| Republic of Benin | 0.01(0.01,0.02) | 0.47(0.28,0.66) | 0.04(0.03,0.05) | 0.56(0.37,0.78) | 0.66(0.58,0.74) |
| Republic of Botswana | 0.05(0.03,0.12) | 7.7(3.98,18.59) | 0.14(0.08,0.37) | 8.82(4.92,22.65) | 0.55(0.37,0.74) |
| Republic of Bulgaria | 0.75(0.53,1) | 6.55(4.69,8.76) | 0.97(0.73,1.24) | 7.51(5.63,9.86) | 0.43(0.38,0.48) |
| Republic of Burundi | 0.01(0.01,0.02) | 0.36(0.19,0.59) | 0.02(0.01,0.04) | 0.33(0.16,0.56) | -0.4(-0.49,-0.32) |
| Republic of Cabo Verde | 0(0,0.01) | 1.41(0.72,2.3) | 0.01(0.01,0.01) | 2.37(1.27,3.35) | 1.72(1.6,1.83) |
| Republic of Cameroon | 0.03(0.02,0.04) | 0.56(0.35,0.78) | 0.09(0.06,0.13) | 0.65(0.44,0.9) | 0.55(0.46,0.65) |
| Republic of Chad | 0.02(0.01,0.02) | 0.41(0.24,0.62) | 0.04(0.02,0.06) | 0.43(0.27,0.63) | 0.24(0.16,0.31) |
| Republic of Chile | 1.84(1.52,2.35) | 17.53(14.53,22.29) | 6.5(5.71,7.27) | 26.22(23,29.22) | 1.72(1.6,1.84) |
| Republic of Colombia | 3.89(3.28,4.75) | 15.39(12.92,18.72) | 15.14(12.69,18.09) | 28.87(24.19,34.6) | 2.57(2.35,2.79) |
| Republic of Costa Rica | 0.36(0.3,0.43) | 16.78(13.79,19.79) | 1.83(1.59,2.1) | 34.97(30.47,40.05) | 2.65(2.51,2.8) |
| Republic of Côte d'Ivoire | 0.03(0.02,0.04) | 0.49(0.29,0.69) | 0.08(0.05,0.11) | 0.59(0.41,0.79) | 0.7(0.63,0.77) |
| Republic of Croatia | 0.83(0.68,1) | 14.56(11.96,17.55) | 3.39(2.83,4.04) | 37.6(31.47,44.84) | 3.75(3.17,4.33) |
| Republic of Cuba | 0.6(0.5,0.71) | 5.7(4.82,6.73) | 1.58(1.36,1.84) | 9.08(7.81,10.58) | 1.97(1.69,2.26) |
| Republic of Cyprus | 0.24(0.12,0.51) | 39.58(17.72,84.44) | 0.5(0.27,0.98) | 24.86(14,47.79) | -1.4(-1.62,-1.19) |
| Republic of Djibouti | 0(0,0) | 0.44(0.24,0.64) | 0(0,0.01) | 0.55(0.34,0.83) | 0.72(0.68,0.75) |
| Republic of Ecuador | 0.46(0.38,0.55) | 6.22(5.14,7.69) | 2.62(2.13,3.19) | 15.7(12.79,19.06) | 3.06(2.25,3.88) |
| Republic of El Salvador | 0.18(0.13,0.29) | 4.67(3.2,7.49) | 0.34(0.23,0.51) | 5.4(3.68,8.11) | 0.66(0.6,0.73) |
| Republic of Equatorial Guinea | 0(0,0) | 0.46(0.28,0.67) | 0.01(0,0.01) | 0.91(0.53,1.36) | 2.49(2.33,2.66) |
| Republic of Estonia | 0.36(0.28,0.46) | 18.14(14.03,23.05) | 1.01(0.84,1.19) | 39.87(33.29,47.17) | 2.84(2.69,2.99) |
| Republic of Fiji | 0.01(0.01,0.04) | 2.46(1.39,5.94) | 0.03(0.02,0.06) | 4.19(2.29,7.08) | 2.55(2.09,3.01) |
| Republic of Finland | 1.5(1.29,1.77) | 20.74(17.85,24.4) | 3.21(2.81,3.6) | 24.22(21.46,27.21) | 0.57(0.3,0.84) |
| Republic of Ghana | 0.04(0.03,0.06) | 0.51(0.35,0.72) | 0.08(0.05,0.12) | 0.32(0.2,0.49) | -2.25(-2.75,-1.75) |
| Republic of Guatemala | 0.45(0.3,0.69) | 7.27(5.09,10.16) | 1.37(1.15,1.64) | 10.65(8.94,12.62) | 1.52(1.37,1.67) |
| Republic of Guinea | 0.02(0.01,0.03) | 0.43(0.25,0.63) | 0.04(0.02,0.05) | 0.51(0.34,0.73) | 0.61(0.54,0.68) |
| Republic of Guinea-Bissau | 0(0,0) | 0.48(0.3,0.7) | 0.01(0,0.01) | 0.6(0.41,0.85) | 0.79(0.72,0.86) |
| Republic of Guyana | 0.03(0.02,0.04) | 5.43(3.99,6.95) | 0.07(0.05,0.1) | 10.34(7.32,13.82) | 2.66(2.5,2.83) |
| Republic of Haiti | 0.38(0.22,0.83) | 7.63(4.24,15.06) | 0.85(0.52,1.6) | 8.93(5.27,16.88) | 0.76(0.68,0.84) |
| Republic of Honduras | 0.45(0.25,0.8) | 13.72(7.6,24.58) | 1.44(0.86,2.49) | 19.52(11.57,32.98) | 1.27(1.22,1.32) |
| Republic of Iceland | 0.03(0.02,0.03) | 8.77(7.72,10.09) | 0.09(0.08,0.1) | 15.02(13.14,16.77) | 2.41(2.02,2.8) |
| Republic of India | 17.91(11.52,28.76) | 3.03(1.85,5) | 56.13(39.54,85.91) | 4.47(3.12,6.86) | 1.55(1.4,1.7) |
| Republic of Indonesia | 3.94(2.68,8.95) | 3.32(2.21,8.03) | 14.02(9.18,29.48) | 5.81(3.77,12.34) | 1.85(1.77,1.92) |
| Republic of Iraq | 0.14(0.09,0.2) | 1.19(0.72,1.79) | 0.4(0.26,0.59) | 1.33(0.88,2.02) | 0.48(0.43,0.53) |
| Republic of Italy | 15.47(12.58,19.4) | 17.96(14.61,22.5) | 55.8(48.84,62.03) | 36.13(31.96,40.43) | 2.19(1.8,2.59) |
| Republic of Kazakhstan | 0.8(0.57,1.16) | 5.28(3.76,7.54) | 1.47(1.14,1.8) | 7.65(5.95,9.37) | 0.82(0.55,1.09) |
| Republic of Kenya | 0.05(0.03,0.07) | 0.4(0.25,0.55) | 0.16(0.1,0.22) | 0.57(0.34,0.81) | 1.18(1.12,1.23) |
| Republic of Kiribati | 0(0,0) | 1.35(0.91,2.06) | 0(0,0) | 1.4(0.91,2.16) | 0.09(-0.02,0.19) |
| Republic of Korea | 5.5(3.66,10.41) | 16.8(11.31,33.21) | 20.99(12.62,34.24) | 24.4(14.72,39.46) | 1.65(1.52,1.78) |
| Republic of Latvia | 0.49(0.35,0.64) | 13.88(10.11,18.33) | 1.82(1.56,2.09) | 49.44(42.37,57.04) | 4.87(4.25,5.5) |
| Republic of Liberia | 0.01(0,0.01) | 0.45(0.28,0.65) | 0.01(0.01,0.02) | 0.47(0.29,0.69) | 0.35(0.24,0.46) |
| Republic of Lithuania | 1.02(0.83,1.25) | 23.3(18.93,28.43) | 2.31(1.95,2.72) | 43.69(36.92,51.19) | 2.07(1.92,2.21) |
| Republic of Madagascar | 0.03(0.02,0.04) | 0.37(0.23,0.54) | 0.07(0.04,0.09) | 0.41(0.25,0.58) | 0.27(0.16,0.38) |
| Republic of Malawi | 0.02(0.01,0.03) | 0.35(0.19,0.55) | 0.05(0.03,0.07) | 0.45(0.22,0.74) | 0.88(0.83,0.92) |
| Republic of Maldives | 0.01(0,0.02) | 8.82(4.04,19.49) | 0.05(0.03,0.1) | 12.63(7.66,22.48) | 1.24(1.06,1.41) |
| Republic of Mali | 0.06(0.04,0.1) | 1.26(0.81,1.89) | 0.2(0.12,0.31) | 1.69(1.08,2.48) | 0.97(0.73,1.21) |
| Republic of Malta | 0.06(0.05,0.07) | 14(12.01,17.03) | 0.25(0.21,0.29) | 25.04(21.24,29.04) | 2.11(1.63,2.6) |
| Republic of Mauritius | 0.03(0.02,0.04) | 4.3(3.12,5.65) | 0.23(0.21,0.25) | 13.21(12.1,14.33) | 3.25(2.37,4.13) |
| Republic of Moldova | 0.34(0.21,0.5) | 7.66(4.65,11.19) | 0.53(0.38,0.73) | 9.32(6.67,12.75) | 0.75(0.71,0.8) |
| Republic of Mozambique | 0.03(0.02,0.04) | 0.33(0.17,0.55) | 0.07(0.04,0.12) | 0.46(0.23,0.78) | 1.3(1.18,1.43) |
| Republic of Namibia | 0.06(0.03,0.13) | 7.59(4.16,18.24) | 0.15(0.08,0.34) | 9.7(5.66,22.45) | 0.85(0.81,0.88) |
| Republic of Nauru | 0(0,0) | 2.79(1.27,4.11) | 0(0,0) | 2.91(1.6,4.35) | 0.21(0,0.42) |
| Republic of Nicaragua | 0.3(0.2,0.53) | 10.46(7.06,18.6) | 0.73(0.49,1.15) | 13.14(8.91,20.63) | 1.24(1.08,1.39) |
| Republic of Niue | 0(0,0) | 2.5(1.24,3.68) | 0(0,0) | 3.33(1.54,5.01) | 0.74(0.64,0.85) |
| Republic of Palau | 0(0,0) | 1.11(0.69,1.63) | 0(0,0) | 1.2(0.75,1.79) | 0.25(0.21,0.29) |
| Republic of Panama | 0.36(0.3,0.42) | 19.11(16.1,22.46) | 1.32(1.06,1.62) | 30.7(24.54,37.6) | 1.88(1.72,2.03) |
| Republic of Paraguay | 0.16(0.1,0.32) | 5.69(3.42,11.7) | 0.61(0.36,1.04) | 10.18(6.03,17.38) | 2.42(2.28,2.56) |
| Republic of Peru | 0.83(0.54,1.27) | 5.15(3.3,7.94) | 1.85(1.2,3.15) | 5.42(3.51,9.18) | -0.17(-0.61,0.28) |
| Republic of Poland | 7.55(6.1,9.51) | 17.49(14.17,22.01) | 21.47(19.18,23.77) | 30.29(27.14,33.47) | 1.77(1.61,1.93) |
| Republic of Rwanda | 0.02(0.01,0.03) | 0.47(0.28,0.77) | 0.04(0.02,0.06) | 0.54(0.3,0.86) | 0.31(0.1,0.52) |
| Republic of San Marino | 0.01(0,0.01) | 15.13(9.51,27.44) | 0.01(0.01,0.02) | 10.95(6.36,20.84) | -0.13(-0.53,0.28) |
| Republic of Senegal | 0.02(0.01,0.03) | 0.48(0.3,0.7) | 0.05(0.04,0.08) | 0.61(0.41,0.84) | 0.81(0.73,0.9) |
| Republic of Serbia | 1.92(1.34,3.06) | 18.65(13.26,30.62) | 3.46(2.46,5.41) | 21.71(15.57,33.46) | 0.93(0.7,1.16) |
| Republic of Seychelles | 0(0,0.01) | 4.89(3.11,11.17) | 0.01(0,0.01) | 5.68(3.49,11.5) | 0.87(0.74,0.99) |
| Republic of Sierra Leone | 0.01(0.01,0.02) | 0.44(0.27,0.65) | 0.02(0.02,0.03) | 0.49(0.32,0.7) | 0.54(0.45,0.64) |
| Republic of Singapore | 0.39(0.25,0.59) | 16.49(10.61,24.91) | 1.36(1.11,1.64) | 17.76(14.54,21.59) | 0.17(0.08,0.27) |
| Republic of Slovenia | 0.5(0.41,0.61) | 20.66(16.91,25.46) | 1.05(0.91,1.22) | 24.18(20.93,28.5) | 0.63(0.26,0.99) |
| Republic of South Africa | 2.91(1.66,4.57) | 12.36(6.62,19.96) | 6.49(4.1,11.97) | 13.67(8.64,24.79) | 0.44(0.33,0.55) |
| Republic of South Sudan | 0.01(0.01,0.02) | 0.32(0.16,0.49) | 0.02(0.01,0.03) | 0.39(0.21,0.62) | 0.63(0.55,0.7) |
| Republic of Sudan | 0.19(0.1,0.41) | 1.27(0.68,3.23) | 0.45(0.26,1.13) | 1.47(0.82,3.97) | 0.65(0.57,0.74) |
| Republic of Suriname | 0.02(0.01,0.04) | 6.51(4.56,12.22) | 0.06(0.04,0.1) | 10.87(7.37,16.92) | 2.52(2.2,2.85) |
| Republic of Tajikistan | 0.04(0.02,0.1) | 0.98(0.54,2.26) | 0.14(0.08,0.26) | 1.64(0.95,2.91) | 2.08(1.87,2.3) |
| Republic of the Congo | 0.01(0.01,0.01) | 0.68(0.51,0.93) | 0.03(0.02,0.03) | 0.77(0.53,1.02) | 0.44(0.37,0.52) |
| Republic of the Gambia | 0(0,0) | 0.49(0.29,0.71) | 0.01(0.01,0.01) | 0.62(0.41,0.88) | 0.89(0.81,0.97) |
| Republic of the Marshall Islands | 0(0,0) | 1.99(0.99,3.1) | 0(0,0) | 2.39(1.22,3.75) | 0.57(0.55,0.59) |
| Republic of the Niger | 0.02(0.01,0.03) | 0.4(0.24,0.59) | 0.05(0.03,0.08) | 0.41(0.24,0.62) | 0.2(0.14,0.27) |
| Republic of the Philippines | 1.64(1.04,2.73) | 4.33(2.7,7.71) | 6.75(4.36,9.61) | 7.71(5.03,11.15) | 2.19(1.91,2.47) |
| Republic of the Union of Myanmar | 0.79(0.46,1.91) | 2.93(1.63,7.2) | 2.41(1.59,4.88) | 4.87(3.24,9.97) | 1.86(1.78,1.94) |
| Republic of Trinidad and Tobago | 0.2(0.16,0.24) | 20.75(17.17,25.31) | 0.52(0.39,0.68) | 30.21(22.32,39.1) | 1.49(1.38,1.59) |
| Republic of Tunisia | 0.1(0.06,0.25) | 1.58(0.89,4.11) | 0.22(0.12,0.59) | 1.71(0.96,4.65) | 0.3(0.26,0.34) |
| Republic of Turkey | 6.71(3.91,12.76) | 16.43(9.27,32.05) | 17.58(11.11,32.83) | 19.74(12.51,36.71) | 0.73(0.64,0.82) |
| Republic of Uganda | 0.03(0.02,0.04) | 0.28(0.15,0.43) | 0.1(0.06,0.15) | 0.47(0.27,0.72) | 1.71(1.64,1.78) |
| Republic of Uzbekistan | 0.18(0.12,0.26) | 1.03(0.68,1.42) | 0.58(0.46,0.72) | 1.83(1.43,2.29) | 1.8(1.58,2.01) |
| Republic of Vanuatu | 0(0,0) | 1.85(0.84,3.02) | 0.01(0,0.01) | 2.06(1.04,3.13) | 0.37(0.32,0.42) |
| Republic of Yemen | 0.11(0.06,0.25) | 1.21(0.63,3.24) | 0.29(0.16,0.7) | 1.31(0.71,3.69) | 0.43(0.27,0.58) |
| Republic of Zambia | 0.02(0.01,0.03) | 0.44(0.27,0.65) | 0.06(0.04,0.08) | 0.61(0.4,0.88) | 1.18(1,1.35) |
| Republic of Zimbabwe | 0.62(0.32,1.08) | 13.36(6.57,24.09) | 1.29(0.77,2.11) | 15.72(9.56,26.5) | 0.58(0.33,0.82) |
| Romania | 2.95(2.06,4.1) | 11.03(7.8,15.2) | 4.79(3.73,6.28) | 14.12(10.84,18.66) | 1.19(0.88,1.49) |
| Russian Federation | 18.76(13.22,25.52) | 10.55(7.51,14.25) | 37.14(29.47,46.43) | 16.31(13.02,20.3) | 1.56(1.36,1.77) |
| Saint Kitts and Nevis | 0(0,0) | 2.5(1.88,3.34) | 0(0,0) | 4.84(4,5.8) | 2.75(2.51,2.99) |
| Saint Lucia | 0.01(0.01,0.01) | 8.54(6.98,10.59) | 0.05(0.04,0.06) | 22.5(18.42,27.11) | 3.76(3.41,4.11) |
| Saint Vincent and the Grenadines | 0.01(0.01,0.02) | 14.73(11.77,18.49) | 0.04(0.03,0.04) | 26.84(22.61,31.64) | 2.32(2.1,2.54) |
| Slovak Republic | 0.45(0.3,0.67) | 7.61(5.07,11.39) | 0.81(0.57,1.13) | 9.1(6.43,12.6) | 0.37(0.13,0.62) |
| Socialist Republic of Viet Nam | 1.51(0.82,4.08) | 3.28(1.77,8.87) | 5.64(3.49,12.75) | 5.79(3.64,13.08) | 2.02(1.95,2.1) |
| Solomon Islands | 0(0,0.01) | 1.85(0.86,3.21) | 0.01(0,0.02) | 2.1(1.02,3.45) | 0.39(0.32,0.47) |
| State of Eritrea | 0.01(0,0.01) | 0.37(0.21,0.61) | 0.02(0.01,0.03) | 0.54(0.33,0.83) | 1.14(0.97,1.32) |
| State of Israel | 1.07(0.92,1.27) | 21.83(18.94,25.77) | 3.26(2.83,3.66) | 25.29(22.15,28.36) | 0.62(0.44,0.79) |
| State of Kuwait | 0.02(0.02,0.02) | 2.08(1.74,2.48) | 0.13(0.11,0.16) | 4.26(3.5,5.15) | 3.65(2.82,4.49) |
| State of Libya | 0.05(0.03,0.13) | 1.95(1.1,5.15) | 0.15(0.08,0.39) | 2.62(1.39,7.2) | 1.23(1.04,1.42) |
| State of Qatar | 0.01(0,0.03) | 7.87(2.59,15.7) | 0.12(0.05,0.26) | 9.37(4.18,19.68) | 1.45(1.07,1.84) |
| Sultanate of Oman | 0.02(0.01,0.04) | 1.8(0.95,4.23) | 0.07(0.03,0.11) | 2.49(0.93,3.73) | 1.79(1.48,2.1) |
| Swiss Confederation | 2.28(2,2.65) | 21.14(18.51,24.6) | 4.87(4.18,5.47) | 25.07(21.89,27.94) | 0.81(0.49,1.13) |
| Syrian Arab Republic | 0.46(0.23,0.86) | 6.21(2.88,12.19) | 0.79(0.44,1.44) | 6.21(3.44,11.78) | 0.01(-0.09,0.1) |
| Taiwan (Province of China) | 1.77(1.09,2.69) | 10.26(6.28,15.5) | 4.36(3.76,4.96) | 11.74(10.19,13.26) | 0.35(0.13,0.56) |
| Togolese Republic | 0.01(0.01,0.01) | 0.5(0.33,0.71) | 0.03(0.02,0.04) | 0.61(0.37,0.93) | 0.7(0.63,0.77) |
| Tokelau | 0(0,0) | 2.18(1.1,3.34) | 0(0,0) | 5.15(1.81,8.86) | 1.34(0.72,1.97) |
| Turkmenistan | 0.04(0.03,0.06) | 1.41(1.05,1.84) | 0.11(0.08,0.15) | 2.3(1.63,3.05) | 1.85(1.67,2.04) |
| Tuvalu | 0(0,0) | 1.94(0.93,3.16) | 0(0,0) | 2.55(1.23,4.07) | 0.87(0.83,0.91) |
| Ukraine | 6.75(4.78,9.13) | 9.79(6.97,13.22) | 8.27(5.98,11.1) | 11.63(8.51,15.18) | 0.56(0.48,0.63) |
| Union of the Comoros | 0(0,0) | 0.43(0.23,0.65) | 0(0,0) | 0.56(0.34,0.83) | 0.64(0.55,0.74) |
| United Arab Emirates | 0.17(0.07,0.37) | 24.06(9.35,44.33) | 0.87(0.49,1.69) | 24.85(14.44,41.34) | 1.6(1.1,2.1) |
| United Kingdom of Great Britain and Northern Ireland | 25.03(20.7,30.7) | 27.26(22.54,33.36) | 40.06(36.06,44.45) | 29.23(26.3,32.56) | -0.08(-0.26,0.09) |
| United Mexican States | 13.37(10.65,17.21) | 20.16(16.57,25.15) | 31.09(27.23,35.4) | 24.59(21.56,27.96) | 0.74(0.53,0.95) |
| United Republic of Tanzania | 0.06(0.04,0.1) | 0.4(0.21,0.64) | 0.19(0.12,0.27) | 0.56(0.33,0.82) | 1.14(1.04,1.23) |
| United States of America | 103.01(90.88,118.74) | 31.86(28.09,36.78) | 187.85(169.2,206.77) | 32.31(29.08,35.6) | 0.04(-0.01,0.1) |
| United States Virgin Islands | 0.01(0.01,0.03) | 16.59(8.76,37) | 0.02(0.01,0.04) | 15.58(7.44,33.5) | 0.14(-0.03,0.32) |

DALYs cases: number of cases / thousands; ASR of DALYs: age-standardised DALYs rate per 100,000 persons;

Abbreviations: DALYs, disability-adjusted life-years; ASR, age-standardized rate; SDI, socio-demographic index; GBD, global burden of diseases, injuries, and risk factors study; EAPC, estimated annual percentage change; CI, confidence interval; MDS/MPN: myelodysplastic syndromes /myeloproliferative neoplasms.


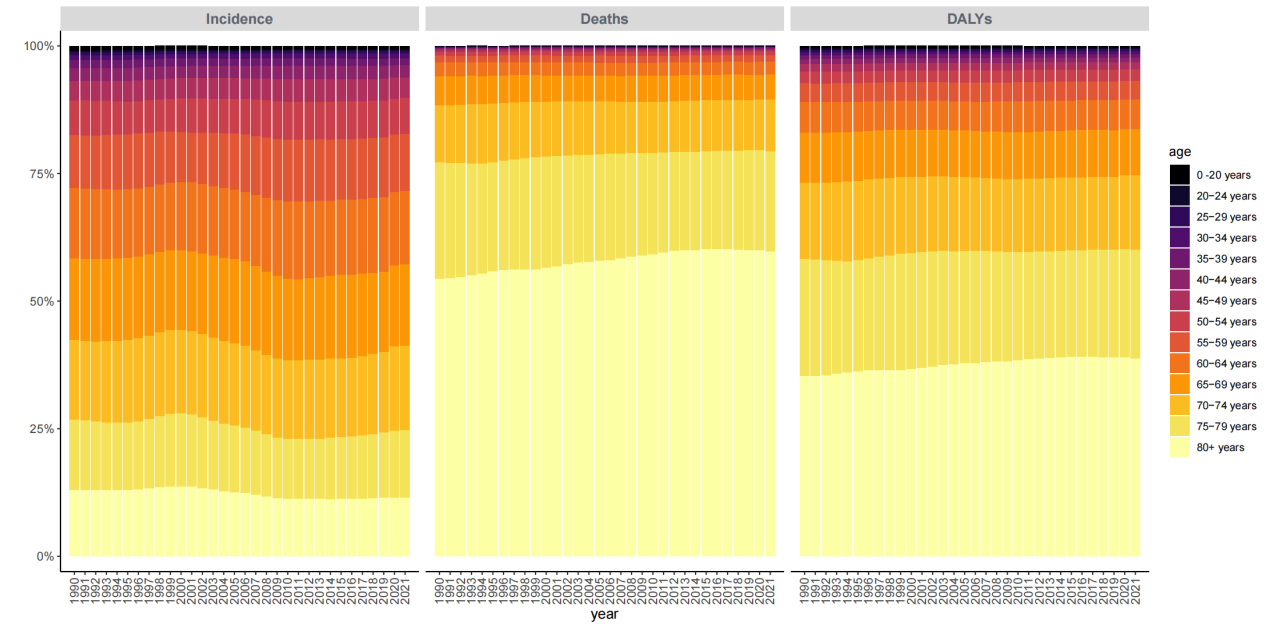


Figure S1 The percentage in ASR of incidence, deaths and DALYs by age, 1990-2021;

Abbreviations: ASR, age-standardized rate per 100,000 persons; DALYs, disability-adjusted life-years.

**Table S5 The case number and ASR of MDS/MPN in 1990 and 2021 in China, with EAPC from 1990 to 2021**

| **measure** | **sex** | **1990** |  | **2021** |  | **1990-2021** |
| --- | --- | --- | --- | --- | --- | --- |
|  |  | **Number cases (95% CI)** | **ASR(95% CI)** | **Number cases (95% CI)** | **ASR(95% CI)** | **EAPCs(95% CI)** |
| Incidence | Both | 32.56(26.33,40.36) | 3.2(2.59,3.97) | 80.88(65.94,99.15) | 3.88(3.21,4.69) | 0.99(0.8,1.18) |
| Incidence | Women | 12.35(9.86,15.21) | 2.45(1.95,3.03) | 28.26(22.8,34.75) | 2.83(2.32,3.39) | 0.71(0.57,0.85) |
| Incidence | Men | 20.21(16.47,25.24) | 3.95(3.2,4.93) | 52.62(42.14,64.93) | 4.95(4.05,6.03) | 1.17(0.95,1.39) |
| Deaths | Both | 1.25(0.62,2.63) | 0.16(0.08,0.34) | 4.74(2.8,8.78) | 0.25(0.15,0.45) | 1.68(1.54,1.82) |
| Deaths | Women | 0.49(0.33,0.86) | 0.11(0.08,0.2) | 1.92(1.29,2.47) | 0.19(0.13,0.24) | 1.72(1.54,1.9) |
| Deaths | Men | 0.76(0.23,2.22) | 0.22(0.06,0.66) | 2.82(0.93,6.95) | 0.33(0.11,0.79) | 1.58(1.43,1.72) |
| DALYs | Both | 73.41(48.26,124.01) | 7.5(4.95,12.69) | 184.69(128.1,288.7) | 9.77(6.77,15.59) | 1.01(0.9,1.11) |
| DALYs | Women | 30.15(22.39,46.51) | 6.13(4.53,9.38) | 74.72(57.14,94.55) | 7.73(6.03,9.71) | 0.82(0.69,0.95) |
| DALYs | Men | 43.26(22.37,92.81) | 9.13(4.82,20.19) | 109.97(58.58,212.12) | 12.03(6.34,23.69) | 1.12(1.01,1.22) |

Abbreviations: ASR, age-standardized rate per 100,000 persons; DALYs, disability-adjusted life-years.

**Table S6 RR of MDS/MPN incidence for both sexes due to age, period, and birth cohort effects**

| **Incidence** | **Women** | | **Men** | |
| --- | --- | --- | --- | --- |
|  | **RR (95%CI)** | **P** | **RR (95%CI)** | **P** |
| **Age(years)**  0-4 | 0.82(0.82~0.83) | <0.001 | 1.35(1.34~1.36) | <0.001 |
| 5-9 | 0.39(0.39~0.39) | <0.001 | 0.54(0.54~0.54) | <0.001 |
| 10-14 | 0.2(0.19~0.2) | <0.001 | 0.17(0.16~0.17) | <0.001 |
| 15-19 | 0.2(0.19~0.2) | <0.001 | 0.14(0.14~0.14) | <0.001 |
| 20-24 | 0.23(0.23~0.23) | <0.001 | 0.16(0.15~0.16) | <0.001 |
| 25-29 | 0.29(0.29~0.3) | <0.001 | 0.2(0.2~0.2) | <0.001 |
| 30-34 | 0.45(0.45~0.45) | <0.001 | 0.31(0.31~0.32) | <0.001 |
| 35-39 | 0.68(0.68~0.69) | <0.001 | 0.48(0.48~0.48) | <0.001 |
| 40-44 | 1.08(1.07~1.09) | <0.001 | 0.78(0.77~0.78) | <0.001 |
| 45-49 | 1.64(1.63~1.65) | <0.001 | 1.19(1.19~1.2) | <0.001 |
| 50-54 | 2.24(2.23~2.25) | <0.001 | 2.14(2.13~2.15) | <0.001 |
| 55-59 | 2.89(2.88~2.91) | <0.001 | 3.59(3.58~3.6) | <0.001 |
| 60-64 | 3.28(3.27~3.29) | <0.001 | 4.38(4.36~4.39) | <0.001 |
| 65-69 | 3.49(3.48~3.51) | <0.001 | 4.65(4.63~4.66) | <0.001 |
| 70-74 | 3.26(3.25~3.28) | <0.001 | 4.27(4.25~4.29) | <0.001 |
| 75-79 | 2.44(2.43~2.45) | <0.001 | 3.14(3.13~3.16) | <0.001 |
| 80-84 | 1.99(1.98~2) | <0.001 | 2.54(2.53~2.56) | <0.001 |
| 85-89 | 1.92(1.9~1.94) | <0.001 | 2.47(2.45~2.49) | <0.001 |
| **Period**  1992-1996 | 0.81(0.8~0.81) | <0.001 | 0.79(0.79~0.8) | <0.001 |
| 1997-2001 | 0.89(0.89~0.9) | <0.001 | 0.88(0.88~0.88) | <0.001 |
| 2002-2006 | 0.97(0.96~0.97) | <0.001 | 0.96(0.96~0.96) | <0.001 |
| 2007-2011 | 1.07(1.07~1.07) | <0.001 | 1.09(1.09~1.09) | <0.001 |
| 2012-2016 | 1.14(1.13~1.14) | <0.001 | 1.15(1.15~1.15) | <0.001 |
| 2017-2021 | 1.18(1.18~1.18) | <0.001 | 1.18(1.18~1.19) | <0.001 |
| **Birth cohort**  1907-1911 | 2.53(2.47~2.6) | <0.001 | 2.73(2.67~2.8) | <0.001 |
| 1912-1916 | 2.33(2.3~2.36) | <0.001 | 2.53(2.5~2.57) | <0.001 |
| 1917-1921 | 2.13(2.11~2.15) | <0.001 | 2.27(2.24~2.29) | <0.001 |
| 1922-1926 | 1.87(1.85~1.88) | <0.001 | 1.99(1.98~2.01) | <0.001 |
| 1927-1931 | 1.66(1.65~1.67) | <0.001 | 1.77(1.76~1.78) | <0.001 |
| 1932-1936 | 1.49(1.48~1.5) | <0.001 | 1.57(1.56~1.57) | <0.001 |
| 1937-1941 | 1.38(1.37~1.38) | <0.001 | 1.45(1.44~1.45) | <0.001 |
| 1942-1946 | 1.29(1.28~1.3) | <0.001 | 1.33(1.33~1.34) | <0.001 |
| 1947-1951 | 1.22(1.22~1.23) | <0.001 | 1.28(1.27~1.29) | <0.001 |
| 1952-1956 | 1.16(1.15~1.17) | <0.001 | 1.23(1.22~1.23) | <0.001 |
| 1957-1961 | 1.12(1.11~1.12) | <0.001 | 1.16(1.16~1.17) | <0.001 |
| 1962-1966 | 1.06(1.05~1.07) | <0.001 | 1.08(1.07~1.08) | <0.001 |
| 1967-1971 | 1.01(1~1.01) | 0.02 | 1(0.99~1) | 0.33 |
| 1972-1976 | 0.93(0.93~0.94) | <0.001 | 0.92(0.91~0.93) | <0.001 |
| 1977-1981 | 0.84(0.84~0.85) | <0.001 | 0.84(0.84~0.85) | <0.001 |
| 1982-1986 | 0.78(0.78~0.79) | <0.001 | 0.79(0.78~0.79) | <0.001 |
| 1987-1991 | 0.73(0.72~0.74) | <0.001 | 0.72(0.71~0.72) | <0.001 |
| 1992-1996 | 0.65(0.64~0.65) | <0.001 | 0.62(0.61~0.62) | <0.001 |
| 1997-2001 | 0.56(0.55~0.57) | <0.001 | 0.53(0.52~0.53) | <0.001 |
| 2002-2006 | 0.49(0.48~0.49) | <0.001 | 0.44(0.44~0.45) | <0.001 |
| 2007-2011 | 0.42(0.42~0.43) | <0.001 | 0.37(0.37~0.37) | <0.001 |
| 2012-2016 | 0.41(0.41~0.42) | <0.001 | 0.36(0.36~0.36) | <0.001 |
| 2017-2021 | 0.42(0.41~0.42) | <0.001 | 0.36(0.36~0.37) | <0.001 |

Abbreviations: DALYs, disability-adjusted life-years; RR, relative risk; CI, confidence interval; MDS/MPN: myelodysplastic syndromes /myeloproliferative neoplasms.

**Table S7 RR of MDS/MPN deaths for both sexes due to age, period, and birth cohort effects**

| **Deaths** | **Women** | | **Men** | |
| --- | --- | --- | --- | --- |
|  | **RR (95%CI)** | **P** | **RR (95%CI)** | **P** |
| **Age(years)**  0-4 | 0.6(0.57~0.62) | <0.001 | 0.52(0.5~0.54) | <0.001 |
| 5-9 | 0.17(0.16~0.18) | <0.001 | 0.16(0.15~0.17) | <0.001 |
| 10-14 | 0.16(0.15~0.17) | <0.001 | 0.14(0.13~0.15) | <0.001 |
| 15-19 | 0.18(0.17~0.19) | <0.001 | 0.16(0.15~0.17) | <0.001 |
| 20-24 | 0.18(0.17~0.19) | <0.001 | 0.16(0.16~0.17) | <0.001 |
| 25-29 | 0.2(0.19~0.2) | <0.001 | 0.2(0.19~0.2) | <0.001 |
| 30-34 | 0.22(0.21~0.23) | <0.001 | 0.24(0.23~0.25) | <0.001 |
| 35-39 | 0.32(0.31~0.34) | <0.001 | 0.27(0.26~0.28) | <0.001 |
| 40-44 | 0.42(0.4~0.43) | <0.001 | 0.39(0.38~0.4) | <0.001 |
| 45-49 | 0.6(0.58~0.62) | <0.001 | 0.54(0.52~0.55) | <0.001 |
| 50-54 | 0.93(0.91~0.96) | <0.001 | 0.89(0.87~0.91) | <0.001 |
| 55-59 | 1.41(1.38~1.44) | <0.001 | 1.48(1.45~1.5) | <0.001 |
| 60-64 | 2.21(2.17~2.25) | <0.001 | 2.51(2.47~2.54) | <0.001 |
| 65-69 | 3.82(3.76~3.87) | <0.001 | 4.39(4.34~4.44) | <0.001 |
| 70-74 | 6.81(6.72~6.9) | <0.001 | 7.79(7.7~7.87) | <0.001 |
| 75-79 | 11.69(11.53~11.86) | <0.001 | 13.44(13.28~13.59) | <0.001 |
| 80-84 | 19.63(19.32~19.95) | <0.001 | 22.58(22.27~22.9) | <0.001 |
| 85-89 | 33.1(32.46~33.75) | <0.001 | 36.98(36.36~37.61) | <0.001 |
| **Period**  1992-1996 | 0.68(0.67~0.69) | <0.001 | 0.68(0.67~0.68) | <0.001 |
| 1997-2001 | 0.8(0.79~0.8) | <0.001 | 0.79(0.78~0.8) | <0.001 |
| 2002-2006 | 0.92(0.91~0.93) | <0.001 | 0.92(0.91~0.92) | <0.001 |
| 2007-2011 | 1.08(1.07~1.09) | <0.001 | 1.07(1.07~1.08) | <0.001 |
| 2012-2016 | 1.28(1.27~1.29) | <0.001 | 1.28(1.27~1.29) | <0.001 |
| 2017-2021 | 1.46(1.44~1.48) | <0.001 | 1.49(1.47~1.5) | <0.001 |
| **Birth cohort**  1907-1911 | 4.02(3.89~4.16) | <0.001 | 4.01(3.89~4.14) | <0.001 |
| 1912-1916 | 3.76(3.66~3.85) | <0.001 | 3.86(3.78~3.95) | <0.001 |
| 1917-1921 | 3.28(3.21~3.34) | <0.001 | 3.42(3.36~3.48) | <0.001 |
| 1922-1926 | 2.99(2.94~3.04) | <0.001 | 3.18(3.14~3.23) | <0.001 |
| 1927-1931 | 2.54(2.51~2.58) | <0.001 | 2.77(2.74~2.8) | <0.001 |
| 1932-1936 | 2.14(2.12~2.16) | <0.001 | 2.36(2.34~2.38) | <0.001 |
| 1937-1941 | 1.81(1.79~1.83) | <0.001 | 2(1.98~2.01) | <0.001 |
| 1942-1946 | 1.61(1.59~1.64) | <0.001 | 1.76(1.74~1.78) | <0.001 |
| 1947-1951 | 1.43(1.4~1.46) | <0.001 | 1.54(1.52~1.56) | <0.001 |
| 1952-1956 | 1.27(1.25~1.3) | <0.001 | 1.33(1.31~1.36) | <0.001 |
| 1957-1961 | 1.15(1.12~1.19) | <0.001 | 1.17(1.14~1.2) | <0.001 |
| 1962-1966 | 1.02(0.99~1.05) | 0.26 | 1.01(0.99~1.04) | 0.31 |
| 1967-1971 | 0.9(0.86~0.93) | <0.001 | 0.88(0.85~0.91) | <0.001 |
| 1972-1976 | 0.8(0.76~0.83) | <0.001 | 0.77(0.75~0.8) | <0.001 |
| 1977-1981 | 0.71(0.68~0.75) | <0.001 | 0.68(0.65~0.71) | <0.001 |
| 1982-1986 | 0.65(0.62~0.68) | <0.001 | 0.6(0.58~0.63) | <0.001 |
| 1987-1991 | 0.59(0.56~0.63) | <0.001 | 0.54(0.52~0.57) | <0.001 |
| 1992-1996 | 0.53(0.51~0.56) | <0.001 | 0.47(0.45~0.49) | <0.001 |
| 1997-2001 | 0.45(0.42~0.47) | <0.001 | 0.39(0.38~0.41) | <0.001 |
| 2002-2006 | 0.36(0.34~0.39) | <0.001 | 0.33(0.31~0.35) | <0.001 |
| 2007-2011 | 0.29(0.27~0.31) | <0.001 | 0.28(0.26~0.29) | <0.001 |
| 2012-2016 | 0.23(0.21~0.25) | <0.001 | 0.23(0.21~0.24) | <0.001 |
| 2017-2021 | 0.18(0.16~0.19) | <0.001 | 0.18(0.17~0.2) | <0.001 |

Abbreviations: DALYs, disability-adjusted life-years; RR, relative risk; CI, confidence interval; MDS/MPN: myelodysplastic syndromes /myeloproliferative neoplasms.

Table S8 RR of MDS/MPN DALYs for both sexes due to age, period, and birth cohort effects

| **Dalys** | **Women** | | **Men** | |
| --- | --- | --- | --- | --- |
|  | **RR (95%CI)** | **P** | **RR (95%CI)** | **P** |
| **Age(years)**  0-4 | 0.77(0.77~0.77) | <0.001 | 0.69(0.69~0.69) | <0.001 |
| 5-9 | 0.27(0.27~0.27) | <0.001 | 0.28(0.28~0.28) | <0.001 |
| 10-14 | 0.27(0.27~0.27) | <0.001 | 0.26(0.26~0.26) | <0.001 |
| 15-19 | 0.28(0.28~0.28) | <0.001 | 0.27(0.27~0.27) | <0.001 |
| 20-24 | 0.28(0.28~0.28) | <0.001 | 0.27(0.27~0.27) | <0.001 |
| 25-29 | 0.3(0.3~0.3) | <0.001 | 0.29(0.29~0.29) | <0.001 |
| 30-34 | 0.32(0.32~0.33) | <0.001 | 0.33(0.33~0.34) | <0.001 |
| 35-39 | 0.44(0.44~0.44) | <0.001 | 0.36(0.36~0.36) | <0.001 |
| 40-44 | 0.55(0.55~0.55) | <0.001 | 0.48(0.48~0.48) | <0.001 |
| 45-49 | 0.75(0.74~0.75) | <0.001 | 0.63(0.62~0.63) | <0.001 |
| 50-54 | 1.06(1.06~1.07) | <0.001 | 0.94(0.94~0.94) | <0.001 |
| 55-59 | 1.46(1.46~1.46) | <0.001 | 1.44(1.44~1.45) | <0.001 |
| 60-64 | 2(1.99~2) | <0.001 | 2.18(2.17~2.18) | <0.001 |
| 65-69 | 2.83(2.83~2.84) | <0.001 | 3.22(3.22~3.23) | <0.001 |
| 70-74 | 4.04(4.04~4.05) | <0.001 | 4.67(4.66~4.68) | <0.001 |
| 75-79 | 5.46(5.45~5.47) | <0.001 | 6.4(6.39~6.41) | <0.001 |
| 80-84 | 7.12(7.1~7.14) | <0.001 | 8.42(8.4~8.44) | <0.001 |
| 85-89 | 9.53(9.5~9.55) | <0.001 | 11.03(11~11.06) | <0.001 |
| **Period**  1992-1996 | 0.77(0.76~0.77) | <0.001 | 0.76(0.76~0.76) | <0.001 |
| 1997-2001 | 0.85(0.85~0.85) | <0.001 | 0.85(0.85~0.85) | <0.001 |
| 2002-2006 | 0.94(0.94~0.94) | <0.001 | 0.94(0.94~0.94) | <0.001 |
| 2007-2011 | 1.06(1.06~1.06) | <0.001 | 1.06(1.06~1.06) | <0.001 |
| 2012-2016 | 1.19(1.18~1.19) | <0.001 | 1.19(1.19~1.19) | <0.001 |
| 2017-2021 | 1.29(1.29~1.29) | <0.001 | 1.31(1.3~1.31) | <0.001 |
| **Birth cohort**  1907-1911 | 2.56(2.54~2.57) | <0.001 | 2.51(2.5~2.53) | <0.001 |
| 1912-1916 | 2.5(2.49~2.51) | <0.001 | 2.53(2.52~2.54) | <0.001 |
| 1917-1921 | 2.28(2.27~2.29) | <0.001 | 2.33(2.33~2.34) | <0.001 |
| 1922-1926 | 2.18(2.17~2.18) | <0.001 | 2.26(2.25~2.27) | <0.001 |
| 1927-1931 | 1.95(1.94~1.95) | <0.001 | 2.06(2.06~2.07) | <0.001 |
| 1932-1936 | 1.72(1.71~1.72) | <0.001 | 1.85(1.84~1.85) | <0.001 |
| 1937-1941 | 1.54(1.53~1.54) | <0.001 | 1.66(1.65~1.66) | <0.001 |
| 1942-1946 | 1.43(1.42~1.43) | <0.001 | 1.53(1.52~1.53) | <0.001 |
| 1947-1951 | 1.33(1.33~1.33) | <0.001 | 1.41(1.41~1.42) | <0.001 |
| 1952-1956 | 1.24(1.24~1.25) | <0.001 | 1.29(1.29~1.29) | <0.001 |
| 1957-1961 | 1.16(1.16~1.17) | <0.001 | 1.18(1.18~1.18) | <0.001 |
| 1962-1966 | 1.06(1.06~1.07) | <0.001 | 1.06(1.06~1.07) | <0.001 |
| 1967-1971 | 0.97(0.96~0.97) | <0.001 | 0.96(0.95~0.96) | <0.001 |
| 1972-1976 | 0.88(0.88~0.88) | <0.001 | 0.87(0.87~0.88) | <0.001 |
| 1977-1981 | 0.8(0.8~0.81) | <0.001 | 0.78(0.78~0.79) | <0.001 |
| 1982-1986 | 0.75(0.74~0.75) | <0.001 | 0.71(0.71~0.71) | <0.001 |
| 1987-1991 | 0.69(0.69~0.7) | <0.001 | 0.65(0.65~0.65) | <0.001 |
| 1992-1996 | 0.63(0.63~0.63) | <0.001 | 0.57(0.57~0.58) | <0.001 |
| 1997-2001 | 0.55(0.55~0.55) | <0.001 | 0.5(0.5~0.5) | <0.001 |
| 2002-2006 | 0.47(0.47~0.48) | <0.001 | 0.44(0.43~0.44) | <0.001 |
| 2007-2011 | 0.4(0.4~0.4) | <0.001 | 0.38(0.38~0.38) | <0.001 |
| 2012-2016 | 0.33(0.33~0.34) | <0.001 | 0.33(0.33~0.33) | <0.001 |
| 2017-2021 | 0.27(0.27~0.27) | <0.001 | 0.28(0.28~0.29) | <0.001 |

Abbreviations: DALYs, disability-adjusted life-years; RR, relative risk; CI, confidence interval; MDS/MPN: myelodysplastic syndromes /myeloproliferative neoplasms.

Table S9 Changes in DALYs number of MDS/MPN according to aging, population growth and epidemiological change from 1990 to 2021 at global level by SDI quintiles.

| **variable** | **Aging** | **Population growth** | **Epidemiological change** | **Overall difference** |
| --- | --- | --- | --- | --- |
| **Global** | 8.6(36.39%) | 10.07(42.61%) | 4.96(21%) | 23.63 |
| **High SDI** | 4.86(44.91%) | 2.94(27.17%) | 3.02(27.91%) | 10.81 |
| **High-middle SDI** | 2.44(38.88%) | 1.17(18.73%) | 2.65(42.38%) | 6.26 |
| **Middle SDI** | 1.47(31.73%) | 1.45(31.3%) | 1.72(36.97%) | 4.65 |
| **Low-middle SDI** | 0.29(17.57%) | 0.71(43.06%) | 0.65(39.37%) | 1.65 |
| **Low SDI** | 0(1.97%) | 0.18(78.68%) | 0.04(19.35%) | 0.23 |

Numbers outside of parentheses represent the change in the number of DALYs for the disease from 1990 to 2021 due to specific factors (e.g., aging, population growth, and epidemiologic changes). Percentages in parentheses represent the proportionate contribution of that factor to the overall change.

Abbreviations: DALYs, disability-adjusted life-years; MDS/MPN: myelodysplastic syndromes /myeloproliferative neoplasms; Aging, ageing of the population.

Table S10 The predicted case number and ASR of incidence, deaths, and DALYs of MDS/MPN from 2020 to 2045 globally

|  | **Incidence** | | **Deaths** | | **DALYs** | |
| --- | --- | --- | --- | --- | --- | --- |
| **Year** | **Case number** | **ASR** | **Case number** | **ASR** | **Case number** | **ASR** |
| 2022 | 352231 | 3.99 | 47692 | 0.49 | 1176399 | 12.78 |
| 2023 | 358070 | 3.97 | 48667 | 0.49 | 1196023 | 12.66 |
| 2024 | 363790 | 3.94 | 49733 | 0.49 | 1216229 | 12.53 |
| 2025 | 369403 | 3.92 | 50889 | 0.48 | 1237182 | 12.40 |
| 2026 | 374916 | 3.90 | 52116 | 0.48 | 1259138 | 12.28 |
| 2027 | 380294 | 3.88 | 53377 | 0.47 | 1281350 | 12.16 |
| 2028 | 385506 | 3.85 | 54692 | 0.47 | 1303609 | 12.03 |
| 2029 | 390568 | 3.83 | 56090 | 0.47 | 1326172 | 11.90 |
| 2030 | 395502 | 3.80 | 57559 | 0.46 | 1349199 | 11.77 |
| 2031 | 400350 | 3.77 | 59078 | 0.46 | 1373046 | 11.65 |
| 2032 | 405106 | 3.75 | 60608 | 0.45 | 1396918 | 11.52 |
| 2033 | 409761 | 3.72 | 62212 | 0.45 | 1420964 | 11.40 |
| 2034 | 414331 | 3.69 | 63927 | 0.45 | 1445498 | 11.26 |
| 2035 | 418797 | 3.66 | 65696 | 0.44 | 1470233 | 11.13 |
| 2036 | 423160 | 3.63 | 67456 | 0.44 | 1495027 | 11.00 |
| 2037 | 427379 | 3.60 | 69116 | 0.44 | 1518484 | 10.87 |
| 2038 | 431420 | 3.57 | 70686 | 0.43 | 1540314 | 10.74 |
| 2039 | 435330 | 3.54 | 72240 | 0.43 | 1561258 | 10.61 |
| 2040 | 439136 | 3.51 | 73800 | 0.42 | 1581944 | 10.47 |
| 2041 | 442890 | 3.47 | 75395 | 0.42 | 1603337 | 10.34 |
| 2042 | 446588 | 3.44 | 77021 | 0.42 | 1625023 | 10.21 |
| 2043 | 450213 | 3.41 | 78666 | 0.41 | 1646593 | 10.07 |
| 2044 | 453788 | 3.38 | 80338 | 0.41 | 1668015 | 9.94 |
| 2045 | 457320 | 3.34 | 82047 | 0.41 | 1689518 | 9.81 |

Abbreviations: ASR, age-standardized rate; DALYs, disability-adjusted life-years; MDS/MPN: myelodysplastic syndromes /myeloproliferative neoplasms.
